# Deciphering the Dynamic Balance Between Solvation Strength and Polysulfides Reaction Heterogeneity in Practical Lithium‐Sulfur Batteries

**DOI:** 10.1002/advs.75640

**Published:** 2026-05-08

**Authors:** Huidong Dai, Pranathi Garlapati, Srinidi Badhrinathan, Luisa Gomes, Tongtai Ji, Dominik Wierzbicki, Yonghua Du, Neville Pavri, Sanjeev Mukerjee, Gaind P. Pandey

**Affiliations:** ^1^ Giner Inc. Newton Massachusetts United States; ^2^ Department of Chemistry and Chemical Biology Northeastern University Boston Massachusetts United States; ^3^ Department of Mechanical Engineering Northeastern University Boston Massachusetts United States; ^4^ National Synchrotron Light Source II Brookhaven National Laboratory Upton New York United States; ^5^ Halocarbon, LLC North Augusta South Carolina United States

**Keywords:** Li–S batteries, polysulfides reaction heterogeneity, practical pouch cell, solid–electrolyte interphase, solvation structure

## Abstract

Achieving stable interfacial chemistry in lithium–sulfur batteries under practical conditions remains a key barrier to commercialization. Here, we demonstrate that interfacial dynamics can be effectively regulated by coupling solvation‐power control with intrinsic heterogeneity of sulfur redox chemistry through the introduction of a weakly solvating fluorinated cosolvent, LIB 1200ET (1200ET). Compared with conventional fluorinated ethers, 1200ET efficiently shifts Li^+^ solvation environment toward a more non‐coordinated configuration at low volume fractions, enabling substantial solvation modulation without significantly impairing sulfur redox kinetics. This solvation transition weakens Li^+^–solvent interactions while strengthening Li^+^–anion and Li^+^–lithium polysulfide (LPS) coordination, suppressing LPS solubility and promoting reconstruction of solid–electrolyte interphase (SEI). Regulated LPS chemistry, together with 1200ET, leads to formation of a S^4+^‐rich, LiF‐reinforced SEI with enhanced ionic conductivity and mechanical robustness. Spatially resolved sulfur K‐edge X‐ray absorption spectroscopy on pouch cells reveals pronounced current‐density‐dependent chemical heterogeneity, distinguishing kinetically dominated and solvation‐controlled regions. Under practical conditions (3.7 mg cm^−2^ sulfur loading, E/S = 6 µL mg^−1^), a single‐layer pouch cell delivers 527 mAh g^−1^ over 200 cycles at C/3, while an Ah‐level multilayer pouch cell achieves an energy density of 358 Wh kg^−1^. These results establish non‐coordinating cosolvent‐driven solvation engineering as a scalable strategy for practical Li–S batteries.

## Graphical abstract

A weakly solvating fluorinated cosolvent (1200ET) enables precise solvation‐power regulation in Li–S batteries, decoupling interfacial stabilization from sulfur redox kinetics. This approach suppresses polysulfide dissolution while preserving reaction kinetics, leading to a stable Li metal interface and high‐energy multilayer pouch cells, revealing a new pathway for electrolyte design.

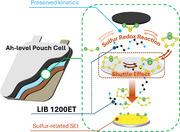

## 1 Introduction

Lithium‐sulfur (Li‐S) batteries are widely regarded as one of the most promising candidates for next‐generation energy storage owing to their exceptionally high theoretical energy density (2600 Wh kg^−1^) and the natural abundance and low cost of sulfur [1, 2]. Despite these intrinsic advantages, the Li–S chemistry is fundamentally encumbered by sluggish multistep sulfur redox reaction (SRR) kinetics, parasitic reactions at the lithium metal anode, and the persistent “shuttle effect” of lithium polysulfides (LPSs), all of which engender intricate interfacial instabilities and ion‐transport heterogeneities [3]. Although these chemical and mechanistic limitations already present formidable obstacles at the material level, their impact is further amplified when Li–S systems are transitioned toward practical, manufacturable cell formats. A major reason for this technological gap lies in the disparity between laboratory‐scale testing and industrially relevant conditions. Most academic studies employ small coin cells with low sulfur loading, excess electrolyte, and thick lithium foil. Such conditions fail to replicate the electrode homogeneity, tortuosity, electrolyte distribution, and current density variations encountered in pouch‐cell configurations [4, 5]. Under industrially relevant conditions such as high areal capacities, lean electrolyte reservoirs, thin lithium anodes, and mechanically confined pouch‐cell architectures, these chemistry‐driven phenomena manifest as pronounced spatial gradients in current density and ion transport that ultimately govern long‐term cycling behavior [4, 6]. Consequently, bridging the persistent divide between fundamental Li–S insights and practical device viability requires rigorous evaluation under realistic operating conditions that capture both the inherent chemical complexity of the Li–S system and the stringent engineering demands of large‐format cells.

At the core of Li‐S battery degradation lies the highly dynamic chemistry of LPSs. During cycling, long‐chain LPSs dissolve from the sulfur cathode and migrate through the electrolyte toward the lithium anode [7, 8]. Driven by concentration gradients, these species participate in parasitic redox reactions with the metallic lithium surface, destabilizing the solid‐electrolyte interphase (SEI) [9]. The resulting SEI becomes compositionally heterogeneous and mechanically fragile [10, 11]. As the lithium metal deforms during repeated plating and stripping, cracks form in the SEI, exposing fresh lithium to further parasitic reactions [12, 13]. The resulting self‐amplifying feedback loop accelerates dendritic deposition, the formation of electrochemically inactive “dead lithium,” rapid electrolyte depletion, and a sharp decline in Coulombic efficiency (CE) [14]. The well‐known shuttle effect further maintains a persistent flux of reactive species to the anode, exacerbating instability—an issue that becomes markedly more severe in pouch‐cell architectures, where nonuniform current distribution, electrolyte stratification, and local transport bottlenecks intensify regional chemical heterogeneity [15, 16]. Consequently, under practical high‐loading, lean‐electrolyte, thin‐anode conditions, the lithium interface experiences far greater electrochemical and mechanical stresses than the sulfur cathode. As a result, the lithium metal anode frequently becomes the earliest point of catastrophic failure in Li–S pouch cells—failing well before the sulfur cathode exhibits significant degradation—ultimately dictating the service life and reliability of the entire system.

Numerous strategies have been explored to mitigate these issues: (i) designing sulfur hosts that physically confine LPSs, (ii) incorporating interlayers or functional separators that adsorb LPSs, (iii) forming protective lithium alloys or artificial SEIs, and (iv) tailoring the electrolyte to regulate LPSs solubility [17, 18, 19, 20, 21]. Among these, electrolyte regulation has emerged as a particularly effective and versatile approach because it directly alters solvation thermodynamics and reaction pathways while simultaneously tuning the E/S ratio—a critical factor for achieving high energy density. In recent years, fluorinated weakly solvating ethers (WSEs) offer a powerful strategy to regulate Li^+^ solvation and suppress polysulfide reactivity in Li–S batteries [22, 23, 24]. However, typical WSEs such as 1,1,2,2‐tetrafluoroethyl‐2,2,3,3‐tetrafluoropropyl ether (TTE) retain residual coordinating capability and therefore must be incorporated at high volume fractions to meaningfully alter the solvation structure. This reliance on large diluent content necessarily displaces strongly coordinating ethers like DME, often at the cost of SRR kinetics [25, 26, 27].

Moreover, such WSEs can also lower the solubility of Li^+^ and long‐chain LPSs while increase viscosity [25, 28]. The elevated viscosity in turn restricts electrolyte infiltration and uniform wetting, particularly when using high‐sulfur‐loading cathodes [29]. Under extreme conditions, these transport limitations may shift the SRR toward a slower, quasi–solid‐state conversion pathway that requires elevated temperature to proceed efficiently [30]. These observations highlight a broader issue: existing WSE systems rely on large amounts of fluorinated diluent to achieve weak‐solvation behavior, but such high diluent content often imposes transport and kinetic penalties. Consequently, a deeper mechanistic understanding of how controlled modulation of solvating power—particularly under practical pouch‐cell conditions—governs polysulfide speciation, solubility, and interfacial chemistry is urgently needed.

In this work, we introduce a commercial fluorinated cosolvent, LIB 1200ET (from Halocarbon LLC, North Augusta, SC; denoted as 1200ET hereafter), a commercially sourced fluorinated ether cosolvent, as an efficient regulator of electrolyte solvation power in Li–S batteries. The molecular structure of LIB 1200ET is disclosed in the relevant Halocarbon patent (U.S. Patent No. US 12381258 B2) [31], while the material itself is commercially available from Halocarbon. We systematically examine its influence on SRR kinetics, SEI chemistry, and interfacial stability under practical conditions. Unlike conventional WSEs, which typically require high volume fractions to perturb the solvation environment, 1200ET exhibits an unusually high solvation‐tuning capability: even at limited concentrations, it drives the Li^+^ solvation structure toward a more non‐coordinating configuration. This enables modulation of Li^+^ coordination and LPSs speciation without displacing coordinating solvents such as DME, thereby maintaining largely intact SRR kinetics. To validate this design principle, we varied the 1200ET content and quantified its effects on Li^+^ solvation behavior, SRR, and interfacial chemistry using Raman, NMR, spatially resolved sulfur K‐edge X‐ray absorption spectroscopy (XAS) and electrochemical analyses. These measurements reveal that appropriately weakened solvation effectively regulates LPSs, promoting the formation of a uniform, S^4+^‐rich, ionically conductive SEI. The finely balanced 1200ET‐modified solvation environment produces a chemically homogeneous and mechanically robust SEI that resists cracking and significantly prolongs cycling life. Importantly, conducting these investigations directly in pouch cells enabled us to resolve current‐induced heterogeneity—variations in local current density and SEI composition—that remain obscured in coin‐cell formats. The dense cathode architecture and lean electrolyte conditions further magnified the subtle but essential chemical effects of the cosolvent, ultimately showing that 1200ET‐containing electrolytes promote improved interfacial uniformity even under spatially non‐uniform electrochemical environments. These findings highlight both the effectiveness of 1200ET as a solvation‐structure modulator and the necessity of probing Li–S electrochemistry under practical pouch‐cell conditions where realistic gradients reveal mechanistic insights otherwise hidden.

Under these optimized conditions, a single‐layer pouch (SLP) cell with a sulfur loading of 3.7 mg cm^−2^ and an E/S ratio of 6 µL mg^−1^ delivered a stable reversible capacity of 527 mAh g^−1^ over 200 cycles at C/3, maintaining an average CE of 95.41%. These results confirm that the cosolvent not only stabilizes the SEI but also sustains efficient sulfur utilization and reversible cycling under lean‐electrolyte conditions. Furthermore, this electrolyte design was successfully extended to an Ah‐level multilayer pouch (MLP) cell with a sulfur loading of 3.5 mg cm^−2^, an E/S ratio of 4 µL mg^−1^ and a 40 µm lithium anode. The MLP configuration sustained a nominal capacity of 1.53 Ah over more than 50 cycles at C/3, demonstrating robust electrochemical stability across multiple stacked layers. The comparable performance between SLP and MLP formats highlights that the incorporation of 1200ET is scalable and directly applicable to practical Li‐S battery assemblies.

Taken together, these findings redefine the role of LPSs in SEI formation, illustrating that with appropriately tuned solvation chemistry, their reactivity can be harnessed to construct a more stable and uniform SEI. They further demonstrate that realistic pouch‐cell configurations are indispensable for unveiling the spatially resolved interfacial phenomena critical to practical Li–S systems. Overall, this work bridges the gap between laboratory‐scale insights and industrial implementation, offering both mechanistic understanding and practical design principles to advance the commercialization of high‐energy‐density Li‐S batteries.

## 2 Data and Discussion

### 2.1 Solvation‐Power Regulation on Li‐Ions and LPSs

The solvating ability of 1200ET was first evaluated using Raman spectroscopy, which differentiates solvent‐separated ion pairs (SSIP) from contact ion pairs (CIP) and aggregated ion pairs (AGG). The compositional ratio between SSIP and CIP / AGG serves as a sensitive indicator of the solvation environment, reflecting the relative coordination of Li^+^ with solvent molecules or anions [25, 32, 33, 34, 35].

**FIGURE 1:**
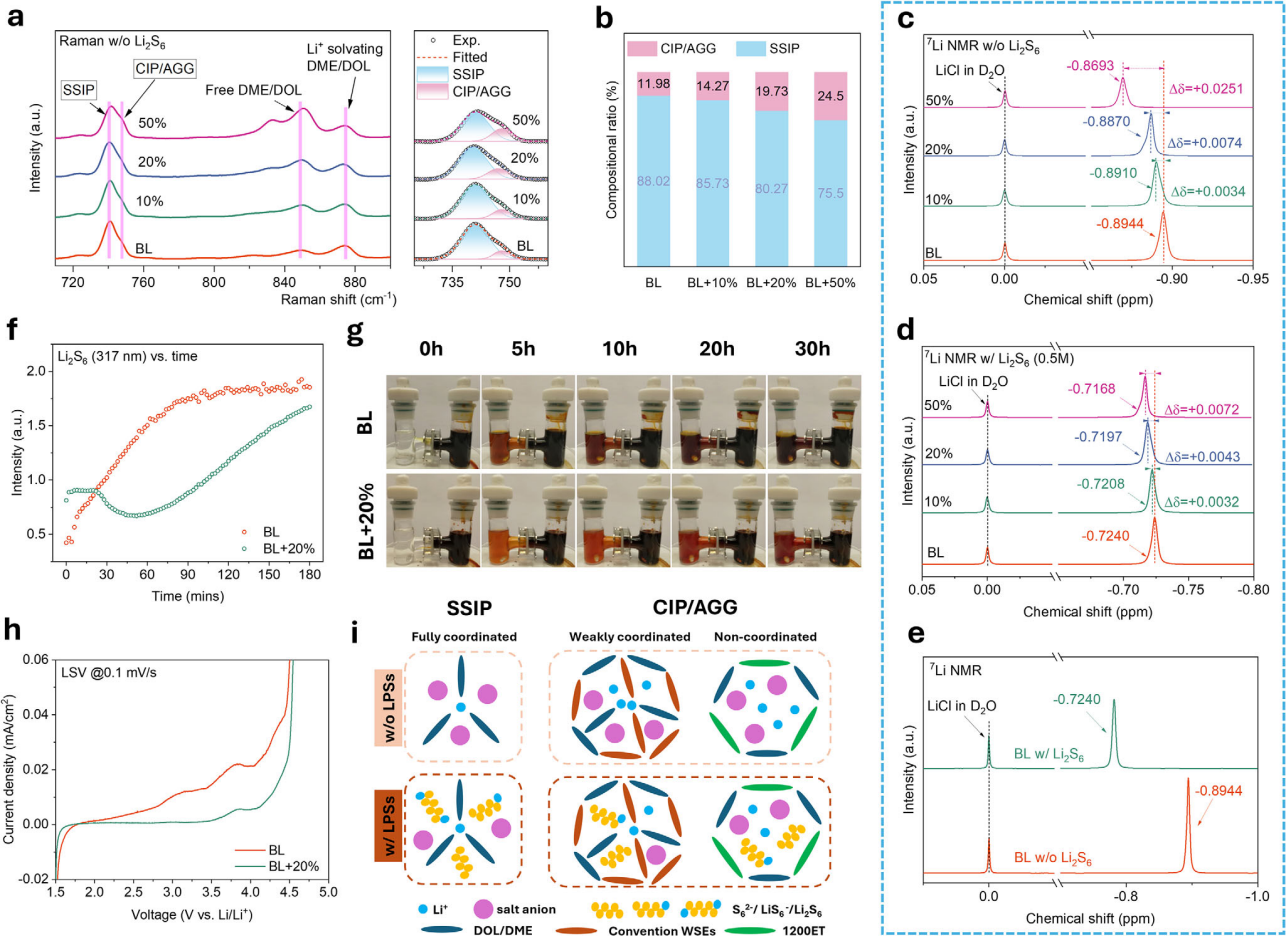
(a) Raman spectra of the baseline electrolyte with varying amounts of 1200ET, along with Gaussian deconvolution of SSIP and CIP / AGG components. (b) Corresponding SSIP and CIP / AGG ratios extracted from the deconvolution. (c‐e) ^7^Li NMR of (c) BL containing different amounts of 1200ET without Li_2_S_6_ and (c) with Li_2_S_6_, and (e) comparison of ^7^Li resonance for BL and BL+20%. (f) Time‐dependent UV‐vis showing the characteristic Li_2_S_6_ absorption band over 180 mins. (g) Time‐lapse photographs showing the diffusion of Li_2_S_6_ across the H‐cell. (h) LSV of the BL and BL+20% up to 4.5 V. (i) Schematic illustration of different Li^+^ solvation structures from fully coordinated to non‐coordinated environments with or without LPSs.

To comprehensively examine the specific role of 1200ET, we added it in four different volume ratios (0, 10, 20 and 50 vol.%, hereafter referred to as BL, BL+10%, BL+20%, and BL+50%) to a baseline electrolyte (BL) composed of 1 M lithium bis(trifluoromethanesulfonyl)imide (LiTFSI) and 0.2 M lithium nitrate (LiNO_3_), dissolved in a 1:1 mixture of 1,3‐dioxolane (DOL) and dimethoxyethane (DME). Figure 1a displays the Raman spectra and Gaussian deconvolution, while Figure 1b summarizes the corresponding SSIP and CIP / AGG fractions (a wider range is provided in Figure S1). As the 1200ET concentration increases, the CIP / AGG fraction rises from 11.98% to 24.5%, indicating a progressive shift toward anion‐rich Li^+^ coordination. This behavior arises from the distinct molecular characteristics of 1200ET. Whereas conventional fluorinated cosolvents such as TTE retain measurable coordinating ability and therefore require high volume fractions to influence Li^+^ solvation, 1200ET exhibits an intrinsically much weaker tendency to interact with Li^+^. As a result, even limited amounts of 1200ET can shift the solvation environment toward a more non‐coordinating configuration. When incorporated into the electrolyte, 1200ET replaces a portion of the coordinating solvent without contributing additional Li^+^–solvent interactions, effectively reducing the overall donor strength of the medium and promoting stronger Li^+^–anion association—consistent with the observed increase in CIP / AGG species. This interpretation is further supported by the ^7^Li NMR results discussed below, which reveal systematic de‐shielding of Li^+^ with increasing 1200ET content, indicative of progressively anion‐rich coordination environments [23, 36, 37].

To further probe the influence of 1200ET on Li^+^ coordination, we conducted ^7^Li NMR using the same 1200ET ratios as in Raman (Figure 1c). After calibrating the spectra with the internal inert reference of LiCl in D_2_O, the ^7^Li chemical shift clearly showed a downfield shift trend with increasing 1200ET concentration, with differential chemical shifts of 0.0034, 0.0074, and 0.0251 ppm for BL+10%, BL+20%, and BL+50%, respectively. This trend is consistent with the Raman results, the electron‐withdrawing CF_3_ groups decrease solvent donicity, causing Li^+^ to experience less shielding and coordinate more strongly with anions [18]. In such environments, Li^+^ resides in increasingly electron‐deficient, anion‐rich configurations, giving rise to the observed deshielding [38].

We next examined the solvent structure in the presence of Li_2_S_6_ to better approximate the working conditions of Li–S batteries. At a higher Li_2_S_6_ concentration (0.5 M), which approaches the upper solubility limit of polysulfides in ether‐based electrolytes, Raman analysis becomes obscured by aggregate‐induced scattering (Figure S2), precluding reliable SSIP / CIP / AGG deconvolution. However, ^7^Li NMR provides clear insights into the Li^+^ coordination environment (Figure 1d). The differential chemical shifts for BL+10%, BL+20%, and BL+50% were 0.0032, 0.0043, and 0.0072 ppm, respectively, exhibiting a monotonic but attenuated increase with 1200ET content. Notably, all values are downfield‐shifted relative to their Li_2_S_6_‐free counterparts (Figure 1e; Figure S3a–c), indicating enhanced deshielding of Li^+^. This is because Li_2_S_6_ is a relatively soft, highly polarizable anion (LiS_6_^−^ / S_6_^2−^) that can form strong contact interactions with Li^+^ [39]. In a solvent environment already biased toward CIP / AGG, Li^+^ will preferentially associate with polysulfide anions (Li^+^···LiS_6_^−^ / Li^+^···S_6_^2−^) rather than being solvated. Therefore, direct coordination to sulfur atoms (or to mixed Li–TFSI–S clusters) reduces local electron density at Li compared to coordination to solvent oxygen further promotes the formation of larger mixed ionic aggregates, which synergistically produce a further downfield shift. Importantly, the relatively weak dependence of the chemical shift on 1200ET concentration under these LPS‐rich conditions suggests that once Li^+^ coordination is dominated by polysulfide species, the local coordination environment becomes comparatively less sensitive to further modulation of solvent donicity.

Interestingly, at a lower Li_2_S_6_ concentration condition (0.25 M), the system exhibits a distinct non‐monotonic behavior at high cosolvent content (Figure S4). Specifically, in BL+50%, the ^7^Li resonance exhibits a smaller downfield displacement than BL+10% and BL+20%, while still remaining more deshielded than the Li_2_S_6_‐free electrolyte. This non‐monotonic trend suggests at excessively high 1200ET content, the extremely weakly solvating environment induces polysulfide precipitation or colloid formation (aggregated S_x_ species). Those colloidal species bind Li^+^ in a different, often less NMR‐visible way (or shield Li^+^ from the fast‐exchanging pool), so the solution‐phase Li^+^ population contributing to the detected ^7^Li resonance is less dominated by strongly Li‐S coordination than at 10–20%, resulting in a smaller net downfield shift. As a result, the apparent chemical shift is reduced despite the intrinsically stronger Li–S interaction. The contrasting trends between 0.25 M and 0.5 M Li_2_S_6_ highlight a regime‐dependent interplay between solvation modulation and aggregation effects: while the former dominates under LPS‐rich conditions, yielding a monotonic response, the latter becomes significant near the solubility threshold, giving rise to non‐monotonic behavior. Collectively, these observations reinforce that 1200ET primarily regulates the bulk solvating environment and Li^+^ association behavior, while the LPS‐dominated coordination structure exhibits a buffered response under highly concentrated conditions.

This observation in ^7^Li NMR is consistent with the Raman observations, which show an increasing fraction of CIP / AGG as cosolvent content rises, implying tighter ionic association and reduced availability of free or weakly bound polysulfide anions. This phenomenon further underscores the necessity of regulating the concentration of weakly solvating cosolvents, especially in the presence of LPSs, as these can dramatically alter the solvation structure compared to when pure Li^+^ are present. As the concentration of weakly solvating cosolvent increases, Li^+^ becomes more coordinated to salt anions (TFSI^−^) and polysulfide anions (LiS_6_^−^ / S_6_^2−^), thereby reducing the effective concentration of freely solvated LPSs.

Ultraviolet–visible (UV–vis) spectroscopy of saturated Li_2_S_6_ solutions (Figure 1f) further supports this conclusion. After calibrating the absorbance using concentration standards (Figure S5), the spectra show that the BL exhibits a continuously increasing Li_2_S_6_ peak at 317 nm, consistent with ongoing dissolution. In contrast, the BL+20% electrolyte displays a distinct non‐monotonic evolution: an initial decrease in peak intensity followed by a gradual recovery to a lower steady‐state value compared to BL. The initial decrease is attributed to reduced polysulfide solubility induced by the weakly coordinating nature of 1200ET. Specifically, the weakened Li^+^–solvent interaction promotes Li^+^ association with polysulfide anions, thereby decreasing the population of freely dissolved Li_2_S_6_ species. This shift in solvation environment likely induces aggregation or colloidal formation of polysulfides, reducing the concentration of optically active, solvated Li_2_S_6_ and leading to a diminished absorption signal.

We note that such non‐monotonic behavior could, in principle, also be associated with redistribution or disproportionation among different LPS species. However, several observations suggest that aggregation / solubility effects dominate in this system. First, the overall spectral features do not show the emergence of distinct new absorption peaks typically associated with significant redistribution into shorter‐chain polysulfides. Second, the BL+20% electrolyte exhibits a markedly elevated spectral baseline in the 600–700 nm region (0.03–0.025 vs 0.017–0.012 for BL), which is characteristic of enhanced light scattering from colloidal aggregates or suspended particles rather than changes in molecular speciation (Figure S6a,b). Finally, the gradual recovery of absorbance over time can be attributed to partial re‐dissolution or dynamic equilibration of these aggregates, rather than a shift toward a fundamentally different polysulfide distribution.

Taken together, these results indicate that the observed spectral evolution primarily reflects a solubility–aggregation equilibrium governed by the weakened solvating environment introduced by 1200ET, rather than a dominant disproportionation pathway. This behavior is consistent with the overall mechanism proposed in this work, where solvation‐power regulation suppresses the availability of freely dissolved polysulfides and promotes their controlled association within the electrolyte.

At a well‐regulated concentration of the weakly solvating cosolvent (20%), such solubility changes were hardly noticeable to the naked eye. As shown in Figure S6c,d, the saturated Li_2_S_6_ solution exhibited little to no visible color change over 48 h. To more sensitively probe this behavior, we conducted a polysulfide diffusion test using an H‐cell configuration (Figure 1g). Each chamber was loaded with a mixture of 5 mL of 0.2 M Li_2_S_6_ and 5 mL of electrolyte (BL or BL+20%), with the two compartments separated by a polypropylene (PP) membrane. Both the time‐lapse snapshot (delayed‐photograph) shown in the figure and the full accelerated time‐lapse video provided in the Supporting Information (SI, Videos S1 and S2; 30‐h experiment condensed to 3 min) clearly demonstrate that the color evolution in BL+20% proceeds significantly more slowly than in BL. This retardation in visual diffusion behavior is consistent with a lower intrinsic solubility of LPSs in the cosolvent‐regulated electrolyte.

The weakly solvating cosolvent also demonstrates superior electrochemical stability, as shown by the linear sweep voltammetry (LSV) in Figure 1h. BL+20% exhibits no significant oxidation peak up to 3.6 V, whereas BL shows continuous oxidation from the start with sharp peaks at 3.2 and 3.7 V (expanded scan in Figure S7). Although higher CIP / AGG composition can promote a more stable SEI with a lower desolvation barrier, it simultaneously reduces bulk ionic conductivity—especially for BL+50% (Figure S8) [25, 26]. These observations highlight the necessity of precisely regulating solvating power, since overly weak solvation can suppress Li^+^ mobility and slow SRR kinetics.

In conclusion, the UV‐vis, Raman, and ^7^Li NMR experiments collectively demonstrate that the solvation structure significantly influences the solubility of Li_2_S_6_ in the electrolyte. Increasing the weakly solvating cosolvents can reduce the solubility of LPSs by weakening Li^+^–solvent interactions, promoting ion pairing with salt anions, and favoring the formation of larger ion aggregates. Raman spectra indicate a shift toward CIP / AGG, while ^7^Li NMR reveals a corresponding downfield shift, reflecting tighter coordination of Li^+^ with anionic species (Figure 1i). This solubility shift has profound implications for the electrolyte's performance, as it could directly affect the formation and stability of the SEI. These findings also underscore the necessity of carefully regulating cosolvent concentration, as overly weakly solvating can lead to exceedingly sluggish SRR kinetics. The dynamic equilibrium between solvation strength and polysulfide reaction is therefore crucial for optimizing the electrolyte composition and ensuring long‐term stability in Li‐S batteries.

### 2.2 Sulfur Redox Reaction (SRR) Kinetics Evaluation

Weakly solvating cosolvents are generally acknowledged to enhance lithium metal stability but simultaneously suppress the redox kinetics of cathodic LPSs. This trade‐off often leads to increased polarization, diminished discharge capacity, and reduced energy density [25, 26, 40]. Consequently, it is essential to assess their influence on cathodic kinetics with exceptional rigor before practical implementation, particularly under realistic high‐loading and lean‐electrolyte conditions.

**FIGURE 2:**
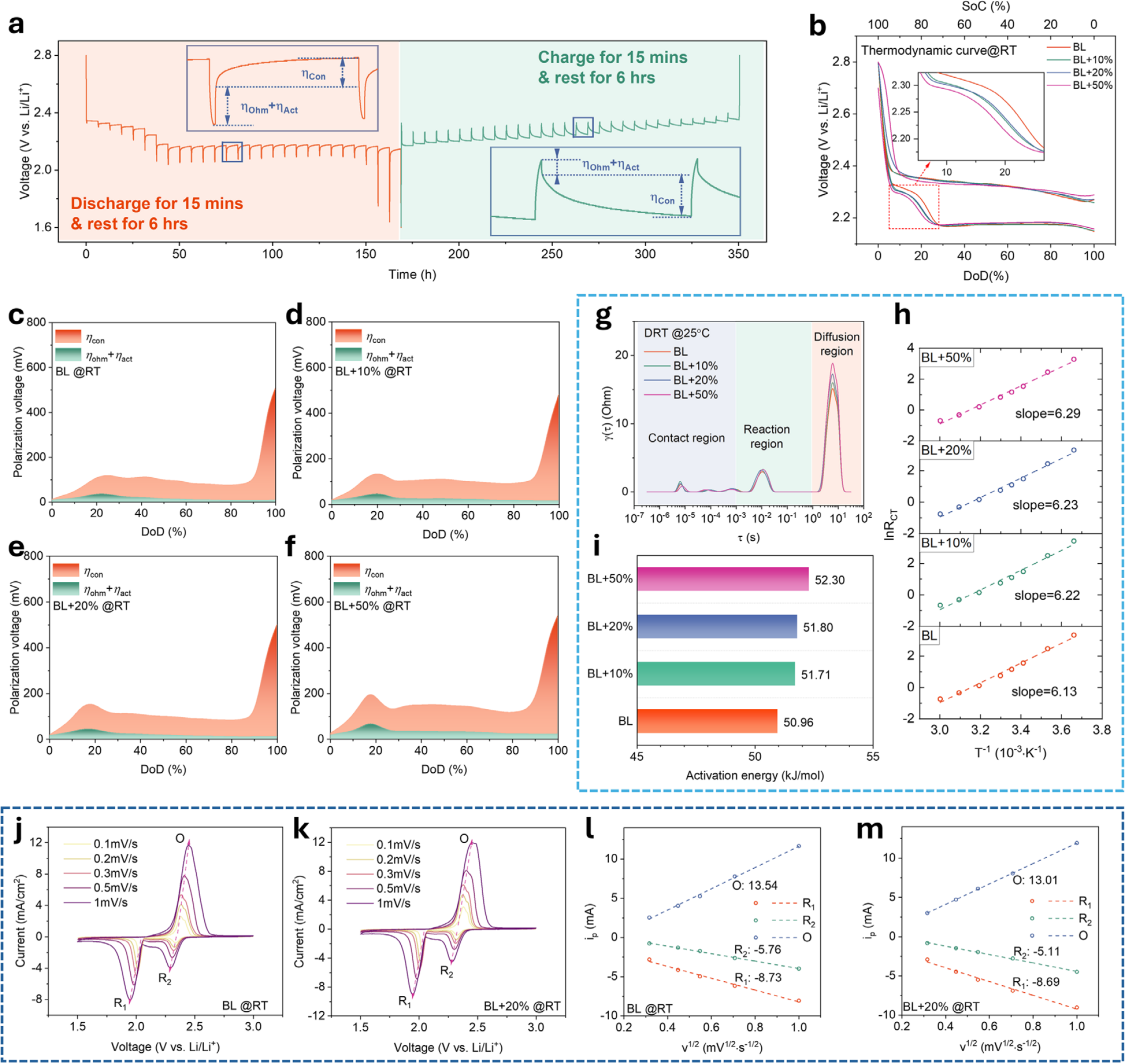
(a) Schematic illustration of polarization decoupling in GITT, shown using BL as an example. (b) Thermodynamic discharge and charge profiles of BL and electrolytes containing different amounts of 1200ET. (c–f) Decoupled specific polarization of BL and various 1200ET concentrations plotted as a function of DoD. (g) DRT analysis of Li_2_S_6_ symmetric cells at RT, and (h) Arrhenius fitting results and (i) corresponding activation energy barriers for charge‐transfer processes in electrolytes with different 1200ET fractions. (j–k) CV curves at multiple scan rates for (j) BL and (k) BL+20%. (l–m) Randles–Sevcik plots for (l) BL and (m) BL+20% for bulk electrolyte diffusion.

To elucidate this effect, the galvanostatic intermittent titration technique (GITT) was employed to probe the cathodic polarization behavior, which effectively captures kinetic limitations arising from weakened solvation [26, 41, 42, 43]. The total polarization (*η*_total_) can be deconvoluted into three primary contributions (Figure 2a): ohmic polarization (*η*_ohm_), activation polarization (*η*_act_), and concentration polarization (*η*_con_). Specifically, *η*_ohm_ arises from ionic and electronic resistance within electrodes and electrolyte; *η*_act_ originates from interfacial charge‐transfer barriers; and *η*_con_ reflects mass‐transport resistance, primarily governed by the diffusion of active species from bulk electrolyte to the electrode surface. Both *η*_ohm_ and *η*_act_ scale directly with current density, as described by Ohm's Law and the Butler–Volmer relation, and therefore vanish immediately once the current is interrupted. In contrast, *η*_con_ evolves with time according to the Nernst–Planck equation and relaxes more slowly after the current ceases.

In many practical Li–S systems, achieving a fully stabilized open circuit voltage (OCV) during GITT rest periods can be challenging, as the relaxation toward true thermodynamic equilibrium is often slower than expected. With this in mind, the present study employed an intentionally rigorous GITT protocol: 15‐min discharge/charge followed by 6‐h relaxation periods, to ensure that residual concentration gradients were minimized. These measurements were carried out under realistic operating conditions, including high sulfur loading (3.8 mg cm^−2^) and lean electrolyte (E/S = 8 µL mg^−1^). Implementing such long relaxation times necessitates exceptional consistency in electrode fabrication and cell assembly but allows for the extraction of reliable kinetic and thermodynamic parameters.

Cathodic polarization behavior was systematically analyzed as a function of the 1200ET cosolvent content. As shown in Figure 2b, increasing the proportion of 1200ET results in a pronounced decline in the thermodynamic discharge voltage between 5–30% depth of discharge (DoD), reflecting elevated overpotentials associated with sluggish LPSs reaction kinetics and reduced solubility. This regime corresponds to the liquid‐to‐solid (or quasi‐solid) transition of LPSs, during which a higher activation barrier is encountered due to constrained solvation dynamics. The full GITT voltage curve and thermodynamic curves are provided in Figure S9.

Detailed polarization profiles (Figure 2c–f) reveal that the total polarization increases progressively with the 1200ET ratio, reaching maxima near DoD ≈ 20% at 119, 133, 160, and 214 mV for BL, BL+10%, BL+20%, and BL+50%, respectively. Interestingly, the combined *η*_ohm_ + *η*_act_ component (green region) remains nearly constant for BL, BL+10%, and BL+20% (37, 45, and 45 mV), only rising sharply to 73 mV at BL+50%. Conversely, *η*_con_ (red region) grows steadily with 1200ET concentration—from 82 to 141 mV—indicating diffusion limitations as the dominant cause of increased polarization.

At moderate concentrations (≤20%), 1200ET appears to regulate mass transport without significantly impeding interfacial charge‐transfer kinetics, distinguishing it from more heavily fluorinated cosolvents like TTE, which often cripple SRR kinetics. However, at high content (50%), both *η*_con_ and *η*_act_ increase markedly, signifying a substantial slowdown in the SRR kinetics and mass transport processes due to the lower LPSs solubility and mobility as demonstrated in the earlier section. These findings emphasize the need for careful compositional tuning of weakly solvating cosolvents. Notably, the charging process remains largely unaffected, showing minimal differences in both thermodynamic curve and polarization voltages (Figure 2b; Figure S10a–d).

The maximum discharge polarization typically occurs near 20% DoD, corresponding to the liquid–liquid conversion between soluble LPSs species, where charge‐transfer kinetics are rate‐limiting. To further probe this process, electrochemical impedance spectroscopy (EIS) and distribution of relaxation time (DRT) analyses were conducted using Li_2_S_6_ symmetric cells. The DRT spectra at 25°C (room temperature, RT) exhibit three distinct peaks with relaxation times (τ) around 10^−5^, 10^−2^, and 10^1^ s, representing contact, reaction, and diffusion regions, respectively (Figure 2g) [26]. The charge‐transfer resistance (R_CT_), a key descriptor of interfacial energy barriers, increases at lower temperatures (0°C, Figure S11), confirming temperature‐sensitive kinetics.

EIS measurements across a range of temperatures (0–60°C, Figures S12–S15) were fitted using the Arrhenius relationship. The slope of ln(R_CT_) vs. 1/T increased slightly from 6.13 to 6.29 as 1200ET content rose from 0% to 50%, corresponding to activation energies of 50.96–52.30 kJ mol^−1^ (Figure 2h,i; Table S1) [17, 26, 44]. The relatively small variation in activation energy—particularly between BL+10% (51.71 kJ mol^−1^) and BL+20% (51.80 kJ mol^−1^)— indicates that moderate 1200ET addition has only a limited influence on intrinsic charge‐transfer kinetics. In contrast, diffusion‐related resistance increases more significantly at higher 1200ET concentrations, consistent with the GITT results. This trend confirms that mass transport limitations, rather than charge‐transfer processes, dominate the overall kinetic penalty. In line with the GITT analysis, although the inclusion of 1200ET increases the total polarization, its effect on activation polarization (LPSs conversion kinetics) remains minimal, while concentration polarization (LPSs diffusion) becomes the primary contributor, particularly when the 1200ET fraction exceeds 20%.

Therefore, cyclic voltammetry (CV) measurements at varying scan rates were performed on Li_2_S_6_ catholytes, followed by Randles‐Sevcik analysis. The CV curves reveal two cathodic peaks (R_1_ and R_2_), corresponding to the reduction of long‐ and short‐chain LPSs, respectively, while one anodic peak (O) represents LPS oxidation (Figure 2j–k; Figures S16a and S17a). Linear fitting of peak current vs. the square root of scan rate indicates a progressive decline in the diffusion‐related slope of peak R_2_ with increasing 1200ET content: 5.76, 5.35, 5.11, and 4.77 for BL, BL+10%, BL+20%, and BL+50%, respectively (Figure 2l–m, S16b, S17b). In contrast, the slopes of peaks R_1_ and O remain largely unchanged, consistent with their solid‐state conversion nature and the negligible polarization differences observed during charging process. Detailed fitting parameters and R^2^ values are summarized in Table 1.

**TABLE 1:** Summary of Randles–Sevcik fitting parameters derived from CV measurements at various scan rates for electrolytes with different 1200ET contents.

	BL	BL+10%	BL+20%	BL+50%
R_1_	−8.78657 ± 0.55445	−8.73701 ± 0.45078	−8.69333 ± 0.36389	−7.54172 ± 0.42458
R_2_	−5.75914 ± 0.09697	−5.34888 ± 0.12575	−5.11399 ± 0.08332	−4.7672 ± 0.12405
O	13.54482 ± 0.3705	13.68434 ± 0.37738	13.0124 ± 0.10007	14.20614 ± 0.36166
R‐Square (R_1_)	0.99058	0.99333	0.9882	0.99208
R‐Square (R_2_)	0.99797	0.9992	0.99834	0.99915
R‐Square (O)	0.99776	0.99772	0.99982	0.99806

Taken together, the combined GITT, EIS, and CV results reveal that the CF_3_‐light fluorinated cosolvent 1200ET exerts a much milder influence on SRR kinetics than typical CF_3_‐rich weakly solvating cosolvents. When maintained below 20%, 1200ET effectively regulate the LPSs solubility and diffusivity by altering the solvation structure without substantially hindering redox kinetics, as supported by Raman and ^7^Li NMR analyses. As the fraction increases to 50%, both GITT‐derived polarization and activation energy exhibit a discernible, though not substantial, increase, indicating that kinetic limitations become measurable but remain relatively moderate. Nevertheless, the accompanying reduction in LPS diffusivity leads to emerging mass transport constraints. This behavior highlights an inherent trade‐off between interfacial stabilization and reaction kinetics: while higher 1200ET content can further suppress polysulfide dissolution, it simultaneously introduces increasing kinetic and transport limitations. Accordingly, 20% 1200ET represents an optimized balance between these competing effects and is therefore adopted for subsequent full‐cell studies.

### 2.3 Lithium Stability Characterization

**FIGURE 3:**
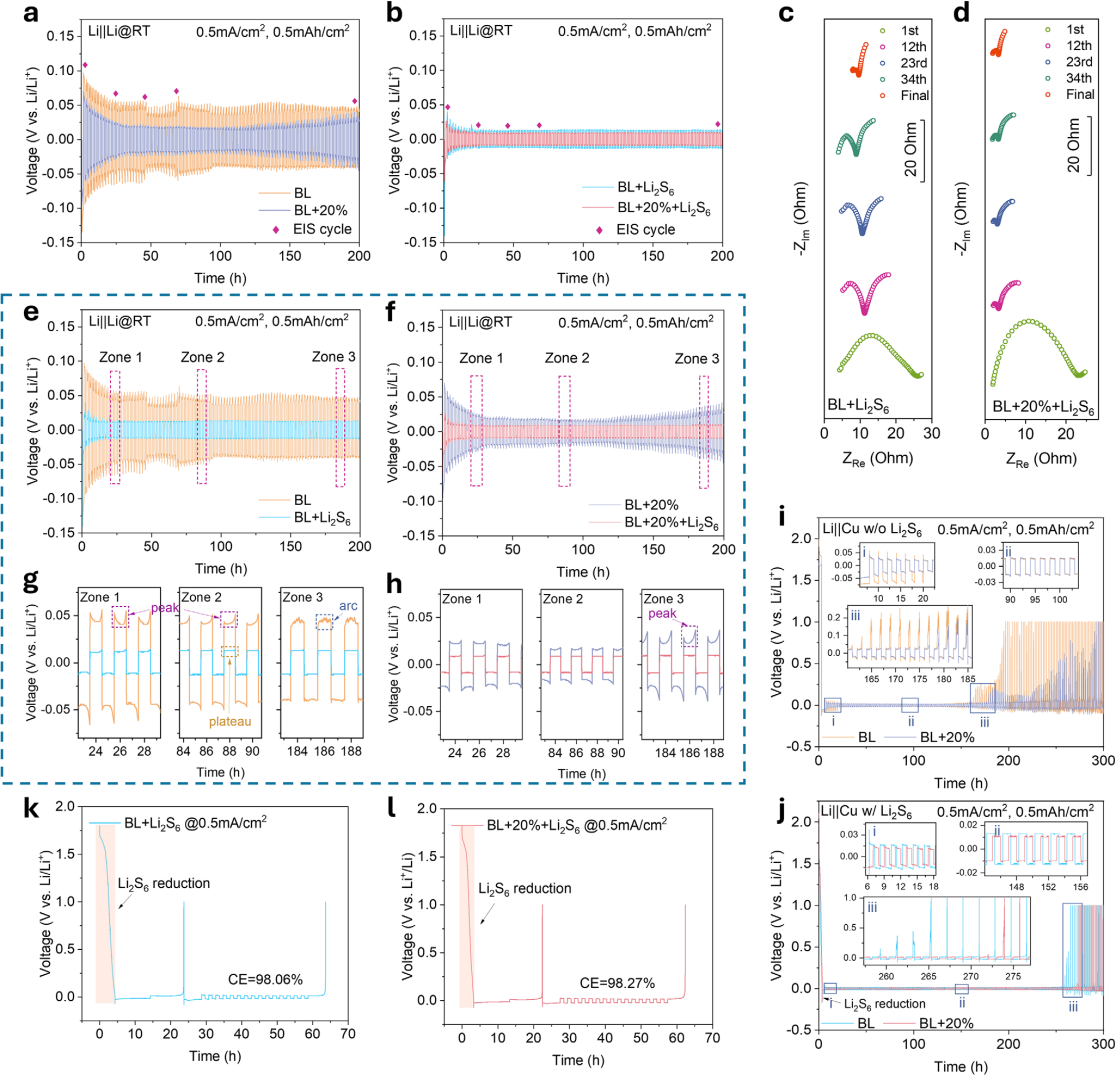
(a–b) Voltage profiles of Li || Li symmetric cells cycled at 0.5 mA cm^−2^ and 0.5 mAh cm^−2^ in electrolytes (a) without and (b) with Li_2_S_6_ for interfacial transport evaluation. (c–d) Corresponding EIS spectra of Li || Li symmetric cells for (c) BL and (d) BL+20%. (e–h) Comparative horizontal views and representative voltage zones for (e–f) BL and (g–h) BL+20%, respectively. (i–j) Voltage profiles of Li || Cu asymmetric cells between BL and BL+20%, cycled at 0.5 mA cm^−2^ and 0.5 mAh cm^−2^, with enlarged insets highlighting specific regions for (i) without Li_2_S_6_ and (j) with Li_2_S_6_. (k–l) Average CE measurements in the presence of Li_2_S_6_ using a modified testing protocol for (k) BL and (l) BL+20%.

To further examine how 1200ET mitigates parasitic reactions and enhances Li metal stability, Li || Li symmetric cells were assembled using both BL electrolyte and BL+20%. The cells were cycled at a current density of 0.5 mA cm^−2^ with an areal capacity of 0.5 mAh cm^−2^ (Figure 3a), and EIS measurements were conducted after every 11 cycles up to 100 cycles (Figure S18a,b). As shown in Figure 3a, BL exhibited a significantly higher voltage polarization compared to BL+20%. Moreover, the overpotential in BL fluctuated irregularly throughout cycling, particularly after the EIS interruptions (around 46 and 70 h), suggesting an inhomogeneity of Li reconstruction on the Li surface. In contrast, the BL+20% electrolyte maintained a much more stable voltage profile, showing minimal disturbance from EIS interruptions. However, a gradual increase in voltage polarization was observed over extended cycling, becoming apparent around 170 h in BL+20%. By the end of the test, the overpotentials of both cells converged to similar levels (46 mV for BL and 42 mV for BL+20%), implying that although 1200ET initially stabilizes the Li interface, the Li surface gradually deteriorates, and SEI degrades during prolonged cycling. Consistently, EIS results showed a comparable trend: both systems exhibited decreasing interfacial resistance (R_SEI_) over cycling, with BL+20% presenting a smaller R_SEI_ at 34 cycles, but nearly identical values at the final measurement (Figure S18a,b).

It is well established that long‐chain LPSs are detrimental to Li metal, inducing parasitic reactions and forming heterogeneous SEI layers [45, 46]. Therefore, it is crucial to evaluate Li stability in the presence of LPSs under weakly solvating cosolvent conditions. To this end, Li || Li symmetric cells containing Li_2_S_6_ were tested followed the same EIS protocol (Figure 3b,c,d). Interestingly, although a slightly higher voltage polarization was observed during the initial cycling stage (before 7 h) due to Li nucleation and SEI formation, both electrolytes exhibited much lower polarization afterward, with overpotentials of 13 mV for BL and 10 mV for BL+20% at the end of haft‐cycle. The corresponding EIS results revealed that BL+20% with Li_2_S_6_ consistently maintained a smaller R_SEI_ across all cycles. This trend persisted even under higher current density of 1 mAh cm^−2^ and areal capacity of 1 mAh cm^−2^, where the stable cycling region (10–108 h) showed overpotentials of 36 and 23 mV for BL and BL+20%, respectively, and 148 and 51 mV at the end of haft‐cycle (Figures S19 and S20).

To better understand this unconventional behavior, a direct comparison was made for each electrolyte system with and without Li_2_S_6_ (Figure 3e,f). Three representative regions (Zones 1–3) were selected to illustrate the voltage polarization evolution during the early, stable, and late cycling stages (Figure 3g,h). Specifically, BL without Li_2_S_6_ exhibited the characteristic galvanostatic voltage response of dynamic Li dendrite growth. During the early and stable cycling stages (Zones 1 and 2), pronounced “peak” voltage responses appeared at the beginning and end of each half‐cycle, reflecting fluctuating Li^+^ nucleation kinetics at the electrode/electrolyte interface and leading to the formation of mossy dendrites and pits. In the late stage (Zone 3), this “peak” response gradually transitioned into an “arc” shape, signifying the accumulation of “dead Li” and increasing isolation of active Li due to poor interfacial contact [14, 41, 45, 47]. Such accumulation results in more tortuous Li^+^ transport pathways and, consequently, significant mass transport limitations.

In contrast, BL containing Li_2_S_6_ displayed a distinctly different voltage profile (Figure 3e,g). During Zone 1, only a slight “peak” appeared at the end of each half‐cycle, accompanied by a markedly lower overpotential, indicating facilitated Li^+^ nucleation kinetics and a developing SEI layer. In Zones 2 and 3, both the “peak” and “arc” features disappeared, replaced by a steady “plateau” response that emerged almost instantaneously at each half‐cycle. This behavior implies that the Li^+^ concentration gradient across the interface had reached a quasi–steady state, reflecting the establishment of a mature SEI that minimized mass transport resistance and suppressed “dead Li” accumulation.

More intriguingly, the BL+20% without Li_2_S_6_ showed a gradually intensifying “peak” behavior with lower overpotential without exhibiting any “arc” response as observed in BL without Li_2_S_6_ (Figure 3f,h). This suggests that even without LPSs, the addition of 1200ET significantly improved Li surface preservation. The absence of severe “dead Li” buildup and reduced tortuosity in Li^+^ transport pathways likely originated from a LiF‐rich SEI formed through 1200ET decomposition. When Li_2_S_6_ is introduced, BL+20%+ Li_2_S_6_ shows a voltage response similar to BL+ Li_2_S_6_ but with an even lower overpotential (∼9–10 mV compared to ∼12–13 mV, Figure 3h), maintaining a stable plateau throughout cycling. It is important to note that this plateau (square‐wave‐like) response originates from stable Li plating/stripping under a well‐regulated interfacial condition, rather than a short‐circuit behavior. In contrast, short‐circuited cells typically exhibit a much lower and nearly constant voltage (∼2–3 mV), which is clearly distinct from the controlled and stable overpotential observed here. These results reveal an often‐overlooked phenomenon: in addition to the stabilizing effect of weakly solvating fluorinated cosolvents, LPSs such as Li_2_S_6_ can actively participate in SEI formation. Rather than merely triggering parasitic reactions, Li_2_S_6_ appears to contribute to the initial Li nucleation and forming a more homogeneous and robust SEI with enhanced Li^+^ transport properties.

Building on this observation, we propose that during the initial cycling stage, Li_2_S_6_ undergoes partial reduction to form a sulfur‐related SEI [46]. This reduction process is closely associated with the Li_2_S_6_ solvation structure—particularly when CIP / AGG dominate—where reactions between Li_2_S_6_ anions (LiS_6_^−^ / S_6_^2−^) and salt anions (TFSI^−^) become more favorable. To further validate this hypothesis, harsh experiments of Li || Cu cells were tested under identical conditions to assess the stability and resilience of SEI layers formed with and without Li_2_S_6_. As shown in Figure 3i, when without Li_2_S_6_, the BL‐based Li || Cu cells exhibited pronounced “peak” behaviors during Li plating (region i), indicating significant nucleation polarization penalty. In contrast, BL+20% showed only a slight “peak” response and much lower overpotential (21–37 mV vs. 61–65 mV for BL), implying reduced nucleation resistance and a more uniform Li deposition. Although both systems were dominated by Li^+^ kinetics during this stage, the smaller overpotential in BL+20% suggests that SEI formation was more effective and less resistive.

During region ii, corresponding to quasi–steady‐state cycling with a more mature SEI, both electrolytes exhibited symmetric “peak” patterns for Li plating and stripping, with nearly identical overpotentials (∼16 mV). This indicates that Li^+^ kinetics primarily governed interfacial processes in both systems. In region iii, however, the BL electrolyte displayed a sharp increase in voltage polarization during stripping (around 163 h), suggesting progressive “dead Li” accumulation and insufficient Li replenishment, leading to an impending drop in CE. In comparison, BL+20% maintained stable voltage behavior, with the sharp rise in polarization delayed until around 180 h.

When Li_2_S_6_ was introduced into both electrolytes, distinct voltage responses emerged (Figure 3j). In region i, both systems displayed only minor “peak” behaviors and lower overpotentials compared to their Li_2_S_6_‐free counterparts (16 mV for BL and 12 mV for BL+20%), indicating smoother Li nucleation on the Cu surface. In region ii, the voltage transitioned to a “plateau” response with slightly reduced overpotentials (13 mV for BL and 10 mV for BL+20%), signifying efficient Li^+^ transport through a well‐formed SEI. In region iii, BL+20% again showed a delayed onset of stripping polarization increase (273 h vs. 258 h for BL), both occurring later than in cells without Li_2_S_6_. This trend is further corroborated by CE evolution: without Li_2_S_6_, BL began to decline around the 95th cycle, while BL+20% showed a delayed drop near the 133rd cycle (Figure S21a). With Li_2_S_6_, the CE decline occurred around the 129th and 142nd cycles for BL and BL+20%, respectively (Figure S21b). Similar behavior was observed under higher current density and areal capacity (Figure S22).

A modified CE testing protocol was then employed to more accurately evaluate the average CE [48]. After the initial Li_2_S_6_ reduction, under 0.5 mA cm^−2^ and 0.5 mAh cm^−2^, the calculated average CE was 97.57% for BL and 97.90% for BL+20% without Li_2_S_6_ (Figure S23a,b). After Li_2_S_6_ addition, the average CE increased to 98.06% for BL and 98.27% for BL+20%, respectively (Figure 3k,l). The same trend persisted under higher current density and capacity (1 mA cm^−2^ and 1 mAh cm^−2^), yielding 97.40% for BL and 97.51% for BL+20% (Figure S24a,b). Together, the Li || Cu results and average CE analysis clearly demonstrate that the initial reduction of Li_2_S_6_ promotes more uniform Li nucleation and yields a mechanically robust SEI capable of enduring repeated plating/stripping cycles. This effect becomes even more pronounced when 20% 1200ET is introduced, as the modified solvation structure facilitates the preferential reduction of Li_2_S_6_ with other Li_2_S_6_ and salt anions. Consequently, a Li^+^ conductive and chemically stable SEI is established, effectively suppressing parasitic reactions on the Li surface, as further supported by shuttle current measurements (Figure S25).

### 2.4 Reaction Inhomogeneity in Li‐S Batteries

To further elucidate the Li_2_S_6_ reduction pathway, operando Raman spectroscopy was conducted under identical sulfur loading (4.2 mg cm^−2^) and E/S ratio (6 µL mg^−1^). To first clarify the spectral evolution during the SRR—particularly the interactions of long‐chain LPSs—the optical cell was potentiostatically held at 2.3 V for 5 h until the current decayed below 0.005 C (Figure S26) [49]. The Raman spectra exhibit a pronounced temporal evolution: the characteristic peaks of elemental sulfur (152, 220, and 473 cm^−1^, Figure S26b) rapidly disappear once the potentiostatic process begins. As the hold continues for approximately 75 min, a new peak emerges at 456 cm^−1^, corresponding to long‐chain LPSs (Li_2_S_x_, 6 ≤ x ≤ 8) [49]. With further reaction time, the response current gradually decays, accompanied by a decrease in the intensity of this 456 cm^−1^ peak as the long‐chain LPSs are reduced to shorter‐chain species and migrate toward the anode. This observation is consistent with both our previous data and reported literature [44, 49].

**FIGURE 4:**
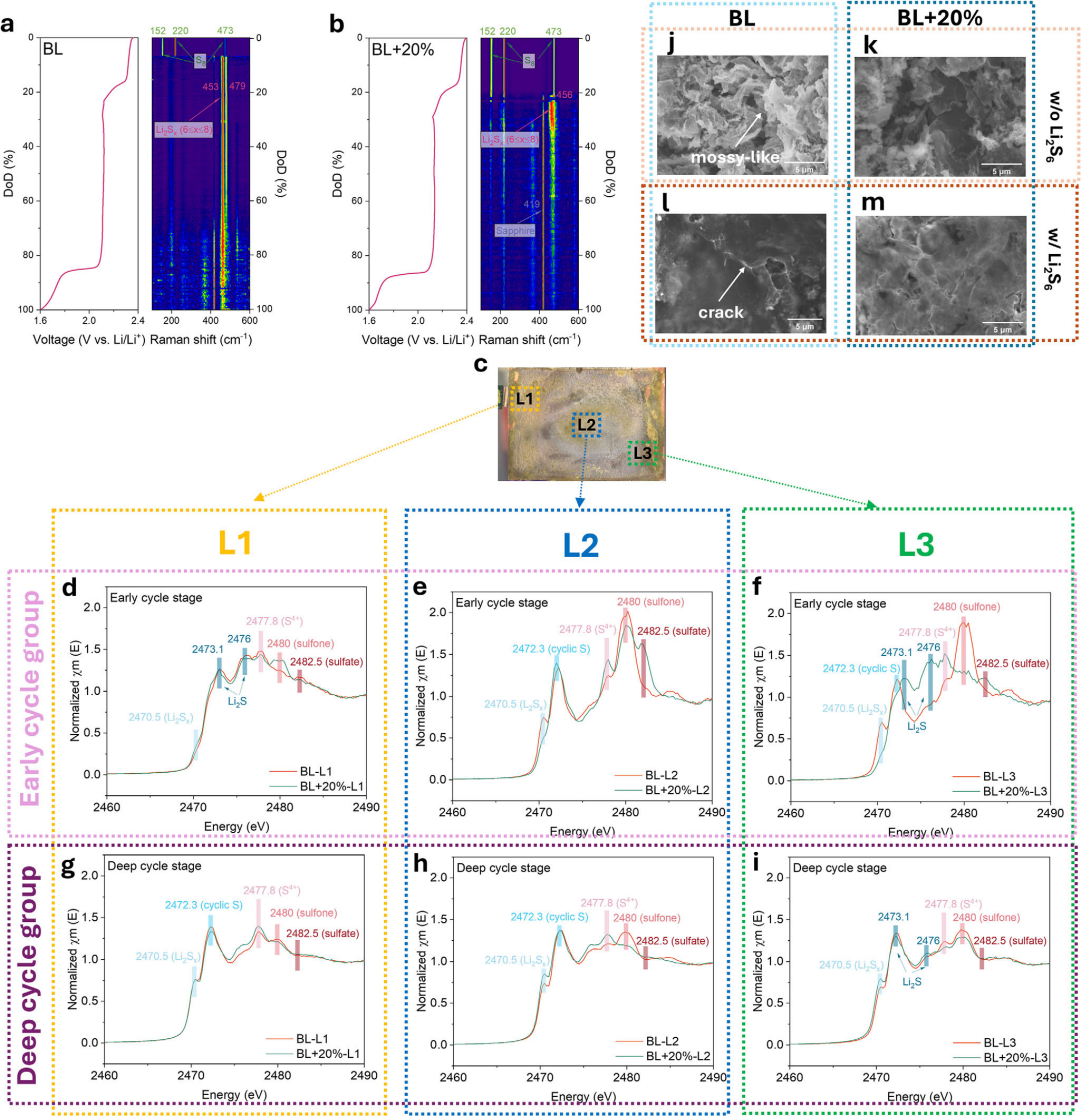
Panels are organized by characterization modality to highlight the mechanistic progression from operando spectroscopy to ex situ interfacial analysis. (a‐b) Operando Raman spectra of its corresponding intensity heatmap aligned with the voltage profiles for (a) BL and (b) BL+20%. (c) Photograph of Li metal harvested from a pouch cell, showing the selected regions L1‐L3, and (d‐i) XANES spectra at sulfur K‐edge for each region during (d‐f) early cycling stage and (g‐i) deep cycling stage, respectively. (j‐m) SEM images of Li surfaces without Li_2_S_6_ for (j) BL and (k) BL+20%, and with Li_2_S_6_ for (l) BL and (m) BL+20%.

The galvanostatic operando Raman experiments reveal a similar pattern. When the cell is discharged galvanostatically at C/20, the voltage profile displays the classical two‐plateau behavior of Li–S systems, representing the sequential conversion of long‐chain and short‐chain LPSs. In BL (Figure 4a; Figure S27a), the elemental sulfur peaks (152, 220, 473 cm^−1^) gradually diminish as discharge proceeds, while new peaks at 453 and 479 cm^−1^ appear around a DoD of ∼8%, signifying the formation of long‐chain LPSs. These peaks subsequently vanish near 70% DoD, aligning well with the voltage plateaus corresponding to the stepwise reduction from elemental sulfur to long‐chain LPSs, and further to shorter‐chain species. A substantial portion of the latter likely migrates toward the anode via the “sulfur shuttle” effect.

Notably, the long‐chain LPS peak splits into two distinct components (456 cm^−1^ vs. 453 and 479 cm^−1^). This splitting arises because, even at a relatively low C‐rate, the galvanostatic process continuously drives the system away from thermodynamic equilibrium, making the chemically sensitive LPS complex more reactive and dynamically evolving within such an environment. Unlike the potentiostatic condition, which maintains a fixed potential and allows the system to approach equilibrium, the galvanostatic mode sustains a fluctuating chemical environment that can become transiently dominated by specific LPS species, each exhibiting slightly different Raman shifts.

In contrast, BL+20% displays markedly different behavior (Figure 4b and Figure S26b). The elemental sulfur reduction proceeds more slowly, and the long‐chain LPSs peak (456 cm^−1^) emerges at around DoD of 21%—significantly later than in the BL electrolyte (8%)—indicating a weaker solvating environment and slower SRR kinetics induced by 1200ET. Moreover, this long‐chain LPSs peak persists only until ∼50% DoD before disappearing, further confirming the altered solubility of LPSs in the presence of a weakly solvating cosolvent. The reduced solubility likely facilitates the formation of a stable SEI, as corroborated by the Li || Li and Li || Cu experiments. It should also be noted that an additional peak at 419 cm^−1^ originates from the sapphire window of the optical cell, as confirmed in our previous study [44].

Unlike small coin cells equipped with thick Li anodes, reaction homogeneity in practical pouch‐cell configurations is considerably more complex because of the demanding conditions associated with high energy density. To assess the influence of 1200ET under such conditions—particularly on SEI formation at the Li anode—a series of single‐layer pouch (SLP) cells were assembled and cycled to distinct stages. Namely, cells stopped at 3 cycles were denoted as early cycling stage, cells stopped above 150 cycles were denoted as deep cycling stages. Cells stopped after 3 cycles were defined as the *early‐cycling stage*, while those cycled beyond 150 cycles represented the *deep‐cycling stage*. After cycling, the cathodes and anodes were harvested following a carefully controlled washing procedure (see “Washing Procedure” in the Supporting Information) inside an Ar‐filled glovebox. The anodes were subsequently characterized using X‐ray absorption near‐edge spectroscopy (XANES) at the sulfur K‐edge.

To probe spatial heterogeneity, each anode was divided into three regions: L1 (near the tab), L2 (center), and L3 (far end, away from the tab), as shown in Figure 4c. Because the cathode and anode tabs are located on the same side, it is reasonable to expect a heterogenous current distribution across the electrode surface—highest and most uniform in L1, moderate and slightly inhomogeneous in L2, and lowest and most heterogenous in L3.

The sulfur K‐edge XANES spectra exhibit several notable features. Comparison with standard references of various LPSs and LiTFSI (Figure S28a,b) reveals transitions at 2470.5 eV (pre‐edge peak), 2472.3 eV (main peak), 2473.1 eV (shifted main peak), and 2476 eV. The transitions at 2470.5 and 2472.3 eV correspond to low‐valence sulfur species associated with LPSs, while those at 2473.1 and 2476 eV are consistent with Li_2_S‐like sulfide environments [50‐–52]. Additional transitions at 2480 and 2482.5 eV may be assigned to oxidized sulfur species, including sulfone groups (residual or decomposed TFSI^−^) and S^6+^ (sulfate‐like species from TFSI^−^ oxidation) [4]. This indicates that, first, the Li anode surface retains residual LPSs or Li_2_S due to “sulfur shuttle” effect, and second, the LPSs and Li_2_S partially participate in SEI formation.

A particularly interesting observation is that BL+20% shows a higher intensity at 2477.8 eV compared to BL, especially at L2 and L3 during the early‐cycling stage (Figure 4e,f) and at L1 and L2 during the deep‐cycling stage (Figure 4g,h). This transitions may be assigned to S^4+^ species, which are often overlooked because research typically emphasizes fluorinated speicies from the cosolvent. However, under a weakly solvating environment, sulfur‐containing species play an equally critical role in SEI evolution. Interestingly, both systems show comparable intensities at L1, with BL+20% exhibiting higher relative‐S^4+^‐type sulfur contribution in the L2 and L3 regions during early cycling.

We interpret this observation as a transition between kinetic/mass‐transport control and thermodynamic/solvation control. According to the Butler–Volmer equation, when the local current density far exceeds the exchange current density (i ≥ i_0_), the overpotential (η) is primarily governed by

$$\eta \approx \frac{RT}{\alpha nF}\ln\left(\frac{i}{i_{0}}\right)$$

rendering the interfacial potential less sensitive to subtle variations in solvation or species activity. At high current densities, enhanced concentration polarization (as i approaches the local limiting current i_L_) depletes interfacial LPSs and anions, shifting the equilibrium toward more reduced sulfur states. In contrast, under low‐current or near‐equilibrium conditions, the Nernst control dominates, and the solvation‐dependent activity of LPSs is directly reflected in the local potential and corresponding XANES spectra, resulting in more pronounced spectral differences.

In the region of lowest current density and most nonuniform current distribution of early cycle group (Figure 4f), the XANES spectra of the two electrolytes diverge significantly. Under these conditions, sluggish interfacial kinetics and steep spatial potential gradients permit persistent local variations in solvation structure, ion transport, and redox intermediates. This heterogeneity amplifies the influence of electrolyte composition—particularly the interplay between LPSs and the fluorinated cosolvent—on the sulfur chemical environment and oxidation‐state distribution Consequently, the spectral divergence observed in these low‐current regions reflects a regime where thermodynamic and solvation effects prevail over kinetic control, exposing the intrinsic differences in sulfur speciation and interfacial coordination induced by the two electrolyte systems.

All of these phenomena are observed during the early‐cycling stage, when the SEI is still developing and remains relatively chemically immature. In contrast, during deep cycling, the XANES spectra across all regions exhibit minimal differences between the two electrolytes (Figure 4g–i), apart from a slightly higher intensity at 2477.8 eV for BL+20%, suggesting the more dominant presence of S^4+^ species within the SEI. This evolution implies that the SEI continues to grow and densify over extended cycling, while localized solvent depletion promotes the formation of a more uniform and chemically stable interphase. Such a matured SEI likely passivates the heterogeneous layers formed in early cycles, thereby mitigating local variations observed initially.

Additionally, during early cycling, the L1 region exhibits a higher Li_2_S content, evidenced by the shifted main peak at 2473.1 eV (Figure 4d). In L2 and L3, this feature persists only in BL+20%, whereas BL shows a pre‐edge peak at 2470.5 eV, indicating a higher concentration of unreacted LPSs (Figure 4e,f). These results confirm that higher current‐density regions (L1) dominate the interfacial sulfur‐redox process, while lower‐current regions (L2 and L3) exhibit greater heterogeneity, particularly in the BL electrolyte. Interestingly, in the deep‐cycling stage, all electrolytes exhibit a pre‐edge peak across L1–L3, attributed to accumulated LPSs from the continuing “sulfur shuttle” effect (Figure 4g–i). We also performed XANES measurements on the cathode side, unfortunately, the high sulfur loading caused significant self‐absorption of the sulfur K‐edge, complicating interpretation of the finer spectral features (Figure S29).

To further examine morphological evolution, scanning electron microscopy (SEM) was performed on the Cu current collector harvested from Li || Cu cells after extended cycling. Without Li_2_S_6_, the Li deposits in BL appear mossy and uneven (Figure 4j), whereas BL+20% exhibits a slightly more homogeneous yet still irregular morphology (Figure 4k). When Li_2_S_6_ is added, the Li morphology in BL becomes denser and smoother but displays visible surface cracks (Figure 4l). In contrast, BL+20% with Li_2_S_6_ shows the most intact and uniform morphology, indicative of a robust and coherent SEI (Figure 4m). Lower‐magnification SEM images (Figure S30) provide an overview of the Cu current collector morphology.

Collectively, the Raman, XANES, and SEM results reveal that introducing a weakly solvating cosolvent fundamentally alters the SEI formation pathway. A mechanically robust, LiF‐rich SEI—typical of fluorinated electrolytes—is accompanied by sulfur‐related species that further contribute to the interfacial structure. Under the weakly solvating environment, the presence of S^4+^ species enhances ionic transport across the SEI, facilitated by redox reactions between LPSs and salt anions arising from a higher CIP / AGG composition in the electrolyte.

### 2.5 Cycling Performance Evaluation in Practical Pouch Cells

**FIGURE 5:**
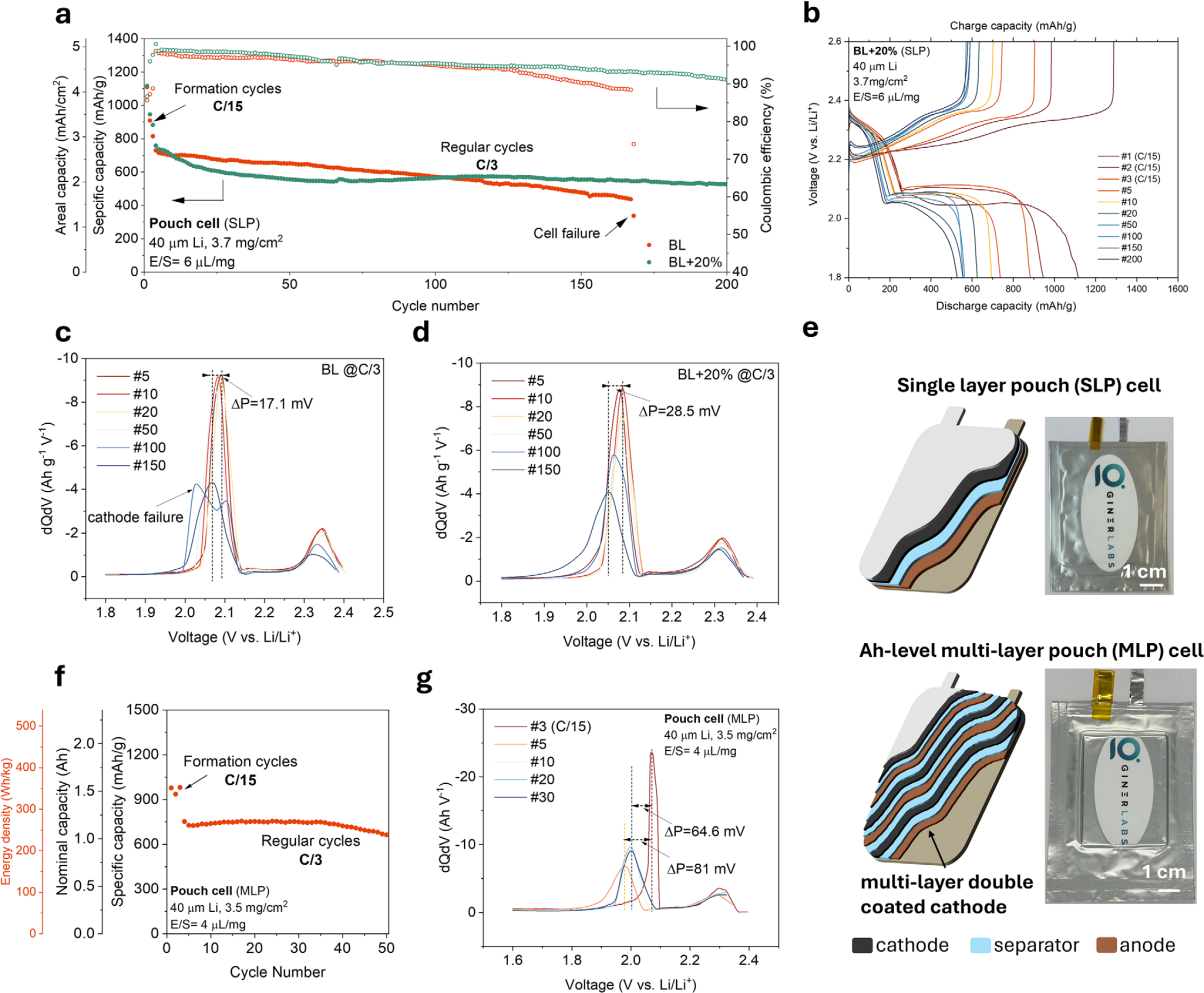
(a) Cycling performance of SLP between BL and BL+20%. (b) Representative voltage profiles of BL+20% in SLP. (c‐d) dQ/dV curves for (c) BL and (d) BL+20% at C/3. (e) Schematic illustrations and corresponding photographs of the SLP and MLP configurations. (f) Cycling performance of MLP comprising eight double‐layer–coated cathodes, and (g) corresponding dQ/dV curves for certain cycles with BL+20%.

To demonstrate the excellent Li stability and corresponding cycling performance under practical conditions, a series of pouch cells—both SLP and MLP configurations—were assembled and evaluated. The cycling behavior of the SLP cell is shown in Figure 5a, with representative voltage profiles at selected cycles in Figure 5b. Notably, without compromising the SRR kinetics, the BL+20% electrolyte sustained over 200 cycles at C/3, delivering an average CE of 95.41% and a final CE of 91.4% with an ultrathin 40 µm Li anode—significantly outperforming the BL cell, which failed after 168 cycles.

The electrochemical evolution in Figure 5a,b highlights the coupled interplay between capacity and interfacial overpotential under different electrolyte conditions. In the BL electrolyte (Figure S31), the cell exhibits a relatively high initial capacity with a small overpotential, followed by a gradual capacity decay accompanied by a continuous increase in overpotential, indicative of progressive interfacial degradation and accumulation of resistive species. In contrast, the BL+20% system displays a distinct non‐monotonic behavior. As shown in Figure 5b, a comparatively larger overpotential is observed during the initial cycles, which can be attributed to the formation of a nascent SEI and interfacial restructuring under the weakly solvating environment; correspondingly, the capacity in Figure 5a is initially lower than that of BL. As cycling proceeds, the overpotential decreases, reflecting the establishment of a more stable and ionically conductive SEI, accompanied by a gradual recovery and eventual surpassing of capacity relative to BL. Upon extended cycling, the overpotential increases again due to the inevitable buildup of interfacial resistance and emerging mass transport limitations, while the capacity remains comparatively stable. This non‐monotonic evolution underscores a key distinction from the BL system, suggesting that although 1200ET introduces a transient kinetic penalty during early‐stage interphase formation, it ultimately enables a more robust and stable interface that sustains improved long‐term electrochemical performance under high sulfur‐loading and lean‐electrolyte (low E/S) conditions.

This interpretation is consistent with the differential capacity (dQ/dV) comparison between BL (Figure 5c) and BL+20% (Figure 5d). At C/3, both electrolytes exhibit two well‐defined discharge peaks at ∼2.3 and ∼2.1 V corresponding to the stepwise reduction of S_8_ to long‐chain LPSs and then to Li_2_S_2_/Li_2_S. Before 150 cycles, BL displays a lower polarization voltage (17.1 mV) than BL+20% (28.5 mV), reflecting less restricted mass transport. However, BL also shows a clear signature of cathode degradation at the 100th cycle, manifested as split peaks at 2.05 and 2.12 V, indicating compromised sulfur utilization due to structural deterioration. Thus, although BL+20% exhibits slightly increased polarization, this trade‐off is more than compensated by its markedly improved long‐term integrity of both sulfur cathode and ultrathin Li anode.

This behavior is further evident in the voltage profiles (Figure S31 for BL and Figure 5b for BL+20%), where BL+20% exhibits a slightly higher discharge overpotential (Figure S32). Despite the increased polarization, the overall electrochemical stability and capacity retention remain superior, underscoring that the optimized solvation structure mitigates Li degradation more effectively than the BL electrolyte.

The rate performance was also assessed using a modified protocol defining 1 C = 1000 mAh g^−1^ (Figure S33∖) [42]. The BL cell exhibited a pronounced capacity loss below 500 mAh g^−1^ at C/2, whereas BL+20% maintained a specific capacity above 600 mAh g^−1^. Upon returning to C/5, BL+20% recovered to 766 mAh g^−1^, significantly higher than BL (652 mAh g^−1^), confirming the superior reversibility and robustness of the cosolvent‐containing electrolyte. Corresponding voltage profiles are provided in Figure S34.

To verify scalability, a more realistic Ah‐level MLP cell was assembled using an 8‐layer double‐coated cathode (schematic and photographs in Figure 5e), sulfur loading of 3.5 mg cm^−2^, E/S ratio of 4 µL mg^−1^, and a 40 µm Li anode. Key engineering parameters are summarized in Table 2. The MLP cell delivered an initial energy density of 358 Wh kg^−1^ (equivalent capacity of 1.53 Ah) and remained stable for more than 50 cycles at C/3. Its regular operating capacity averaged 732 mAh g^−1^ (ending at 662.8 mAh g^−1^), corresponding to an average nominal capacity of 1.15 Ah and average energy density of 262 Wh kg^−1^ (Figure 5f). For comparison, the cycling performance of the BL MLP cell is provided in Figure S35, which shows a noticeably faster capacity decay after formation, further highlighting the advantage of the 1200ET system. The dQ/dV analysis further shows that after transitioning from C/15 to C/3 operation, the initial polarization of 81 mV decreased to 64.6 mV, indicating preserved structural stability and acceptable cathode kinetics even under the mechanically demanding multilayer pouch‐cell architecture. A representative voltage profile is shown in Figure S36.

**TABLE 2:** Summary of key engineering parameters for the MLP (4.45 × 5.35 cm^2^) comprising eight double‐layer–coated cathodes.

Parameters	Value
Cathode weight (including CC and tab)	3.51 g
Coating weight	2.46 g
Sulfur weight	1.574 g
Electrolyte amount	6.3 mL
Anode weight (including CC and tab)	4.42 g
Nominal capacity	1.53 Ah

Overall, the enhanced pouch‐cell performance arises from the synergistic role of the 1200ET in modulating LPSs solvation and interfacial chemistry. During cycling, the LPSs species formed through the SRR inevitably migrate to the anode via the “sulfur shuttle” effect, where they interact with salt anions and participate in SEI formation. The resulting interface adopts the mechanical robustness of the fluorinated cosolvent–derived LiF‐rich SEI, while the concurrent redox activity of LPSs introduces sulfur‐containing species that enhance ionic transport. Consequently, a composite SEI is established that combines mechanical resilience and high ionic conductivity, thereby extending the lifespan of Li–S batteries even under stringent pouch‐cell conditions.

## 3 Conclusions

This study establishes a comprehensive mechanistic framework connecting solvation thermodynamics, sulfur redox heterogeneity, and interfacial chemistry in practical Li–S batteries. By introducing a fluorinated weakly solvating cosolvent (1200ET), we achieved deliberate modulation of the Li^+^ solvation sheath and polysulfide activity, thereby stabilizing both the cathodic and anodic interfaces. Spectroscopic and electrochemical analyses reveal that 1200ET induces a transition from SSIP to CIP / AGG ion pairs, suppressing polysulfide solubility and promoting SEI formation enriched in LiF and S^4+^ species. This composite SEI exhibits enhanced ionic transport and mechanical robustness, effectively mitigating dendrite growth and parasitic reactions even under lean‐electrolyte and high‐loading conditions. Synchrotron‐based XANES of large‐format pouch cells further uncovered spatially resolved reaction inhomogeneities arising from current‐distribution gradients—an intrinsic feature of practical cell architectures. The weakly solvating electrolyte mitigates these effects, enabling a chemically homogeneous and stable SEI across the Li surface. The scalability of this design was validated in Ah‐level multilayer pouch cells, confirming its industrial feasibility.

Overall, this work reframes LPSs not as deleterious intermediates but as dynamic agents in constructing a conductive and resilient SEI when governed by optimized solvation chemistry. The insights presented herein bridge the long‐standing divide between laboratory and practical Li–S systems, providing a rational strategy for electrolyte design that harmonizes solvation strength, interfacial stability, and long‐term durability in next‐generation high‐energy batteries.

## Author Contributions

HD: Conceptualization, Visualization, Methodology, Investigation, Formal analysis, Data curation, and Writing – original draft. PG: Synthesis, Electrochemical Experiments, Investigation, and Writing – review & editing. SB: Synthesis, Electrochemical Experiments, Writing – review & editing. LG: Materials Characterizations, Experiments, formal analysis, Writing – review & editing. TJ: Formal analysis, Writing – review & editing. DW and YD: Synchrotron‐based Experiments, Formal Analysis, and Writing – review & editing. NP: Fluorinated cosolvent synthesis, Writing – review & editing. SM: Supervision, Resources, Project Administration, and Writing – review & editing. GPP: Conceptualization, Visualization, Investigation, Supervision, Resources, Project administration, Funding acquisition, and Writing – review & editing.

## Conflicts of Interest

The authors declare that the fluorinated ether LIB 1200ET used in this study is a proprietary material of Halocarbon, LLC and is protected under the company's intellectual property portfolio (U.S. Patent No. US 12381258 B2), in which its molecular structure is disclosed. For this reason, the full chemical structure is not reproduced in this publication, and readers are referred to the cited patent for detailed structural information. One of the co‐authors, Neville Pavri, is an inventor associated with the aforementioned patent and is affiliated with Halocarbon, LLC. The remaining authors declare no competing financial interests.

## Supporting information




**Supporting File 1**: advs75640‐sup‐0001‐SuppMat.pdf.

**Supporting File 2**: advs75640‐sup‐0002‐VideoS1.mp4.

**Supporting File 3**: advs75640‐sup‐0003‐VideoS2.mp4.

## Data Availability

The data that support the findings of this study are available from the corresponding author upon reasonable request.
