# Supplementary material for: Deciphering the Dynamic Balance Between Solvation Strength and Polysulfides Reaction Heterogeneity in Practical Lithium‐Sulfur Batteries
Source: Adv Sci (Weinh). 2026 May 8;13(44):e75640. doi: 10.1002/advs.75640 (PMC13336008; doi:10.1002/advs.75640)
Supplement: Supplementary file 1 [file ADVS-13-e75640-s002.pdf]

Supporting Information For

Deciphering the Dynamic Balance Between Solvation Strength and Polysulfides Reaction Heterogeneity in Practical Lithium-Sulfur Batteries

Huidong Dai[1,2,], Pranathi Garlapati[1], Srinidi Badhrinathan[1], Luisa Gomes[2], Tongtai Ji[3], Dominik Wierzbicki[4], Yonghua Du[4], Neville Pavri[5], Sanjeev Mukerjee[2*], Gaind P. Pandey[1*]*

[1]Giner Inc., 89 Rumford Avenue, Newton, Massachusetts, 02466, United States

[2]Department of Chemistry and Chemical Biology, Northeastern University, 360 Huntington Avenue, Boston, Massachusetts, 02115, United States

[3]Department of Mechanical Engineering, Northeastern University, 360 Huntington Avenue, Boston, Massachusetts, 02115, United States

[4]National Synchrotron Light Source II, Brookhaven National Laboratory, Upton, New York, 11973, United States

[5]Halocarbon, LLC, 1100 Dittman Ct, North Augusta, South Carolina, 29841, United States

[*]Corresponding authors: h.dai@northeastern.edu (H. Dai); s.mukerjee@northeastern.edu (S. Mukerjee); gpp.ssi@gmail.com (G.P. Pandey)

Figures S1 to S36, Table S1, Ref. S1 to S7

1. Experimental Section

1.1. Materials

Elemental sulfur (S_8), lithium sulfide (Li_2S), and N-methylpyrrolidone (NMP) were purchased from Sigma Aldrich. Lithium nitrate ($LiNO_3$), 1,3-dioxolane (DOL), 1,2-dimethoxyethane (DME) were purchased from Fisher Scientific. Lithium bis(trifluoromethane)sulfonimide (LiTFSI) was purchased from Solvionic. Ketjenblack (EC 600JD) was supplied by Fuel Cell Store, Super P was purchased from MES Supplies, and Poly (acrylic acid) solution (Mw ~250,000) was obtained from Sigma-Aldrich. Polypropylene (PP) separators (Celgard 2400) were purchased from Celgard and carbon-coated aluminum foil were supplied by Armor Battery Films. Copper foil and lithium metal foil were purchased from MTI Corp. The fluorinated cosolvent LIB 1200ET (hereafter denoted as 1200ET) was obtained as a commercially available fluorinated ether from Halocarbon, LLC (North Augusta, SC, USA); its molecular structure is disclosed in the corresponding Halocarbon patent cited in this work. All materials were used as received without further purification.

1.2. Preparation of electrolytes compositions

The 1200ET cosolvent was first blended with a DOL/DME (v/v = 1:1) solvent mixture at volume ratios of 0, 5, 10, 20, and 50%. Each mixed solvent was then used to dissolve 1 M LiTFSI and 0.2 M $LiNO_3$, producing the electrolytes designated as BL, BL+5%, BL+10%, BL+20%, and BL+50%, respectively. These formulations were subsequently used for ionic conductivity measurements, preparation of Li_2S_6 solutions, Li || Li symmetric cells, Li || Cu asymmetric cells, and all kinetics- and diffusion-related studies.

For shuttle current measurements, the electrolytes were prepared using the same procedure but without the addition of 0.2 M $LiNO_3$.

1.3. Preparation of lithium polysulfide solutions

Li_2S_6 solutions were prepared by dissolving elemental sulfur (S_8) and Li_2S in BL and the mixed electrolytes at a molar ratio of 5 : 8. The electrolyte was prepared following the procedure described in Section 1.2. The mixtures were stirred at 300 rpm on an 80 °C hotplate inside an Ar-filled glovebox overnight. The resulting stock solution was then prepared to obtain 0.25 M Li_2S_6 solution, which was used for ^{7}Li NMR and UV–vis analyses, Li || Li symmetric cells, Li || Cu

asymmetric cells, Li || Li_2S_6 cell, Li_2S_6 symmetric cells, and all kinetics- and diffusion-related experiments. In addition, a more concentrated Li_2S_6 solution (0.5 M) was prepared specifically for ^{7}Li NMR measurements to probe concentration-dependent solvation behavior.

1.4. Preparation of carbon/sulfur cathodes

The carbon/sulfur (C/S) composite was prepared by mixing elemental sulfur (S_8) and Ketjenblack at a 4 : 1 weight ratio, followed by ball milling for 48 hrs. The milled mixture was subsequently melt-infused at 155 °C for 15 hours. To fabricate the cathode slurry, the C/S composite (80 wt.%), Super P (10 wt.%), and LiPAA binder (10 wt.%) were dispersed in DI water to achieve a solids content of approximately 15%. LiPAA was prepared by lithiating PAA using LiOH by neutralization reaction. The slurry was homogenized using a DAC 150.1 FVZ-K mixer (FlackTek Inc.) and cast onto a 15 μm carbon-coated aluminum foil, yielding a sulfur areal loading of approximately 4 mg cm^{-2}. The electrodes were dried at 80 °C for 4 hours before being transferred into an Ar-filled glovebox for coin cell assembly. Also, electrodes were punched in-size for single-layer pouch cells (SLPs) and multi-layer pouch cells (MLPs)

1.5. Coin cell assembly procedure

Coin cells were assembled using stainless steel 316 CR2032 cases (MTI Corp.) and sealed with a digital pressure-controlled crimping system (MSK-160E, MTI Corp.). Cathodes were punched into 12.7 mm disks and paired with 15.8 mm lithium metal disks (600 μm thick) as the anode. A single layer of 19.05 mm PP separator, along with a spacer and wavy spring, was included in each cell stack. All assembly steps were carried out inside an Ar-filled glovebox (Vigor; H_2O and O_2 levels < 1 ppm). After assembly, all coin cells were rested for 12 hours prior to electrochemical testing.

1.6. Pouch cell assembly procedure

The pouch cells employed double-sided Li metal sheets (40 μm Li on each side) laminated onto Cu current collectors as the anode and were obtained from Albemarle Corporation (Kings Mountain, NC, USA). For single-layer pouch (SLP) cells, both cathode and anode sheets were cut to 3.65 × 5.35 cm^2, while multilayer pouch (MLP) cells used electrodes cut to 4.45 × 6.45 cm^2. The SLP configuration consisted of one cathode–anode pair separated by a PP separator, whereas

the MLP design incorporated eight double-layer stacks. Electrical connection of the electrode stacks was achieved using Al (cathode) and Ni (anode) tab terminals. The sulfur loading was 3.7 mg cm^{-2} with an electrolyte-to-sulfur (E/S) ratio of 6 µL mg^{-1} for SLP cells, whereas for MLP cells the sulfur loading was 3.5 mg cm^{-2} with an E/S ratio of 4 µL mg^{-1}.

1.7. Assembly of Li || Li symmetric cells, Li || Cu asymmetric cells, Li || Li_2S_6 cells, and Li_2S_6 symmetric cells

The Li || Li symmetric cells were assembled using two identical 11.13 mm lithium metal disks with carbon-coated aluminum foil current collectors separated by a 19.05 mm PP separator. Each cell contained 20 µL electrolyte (with or without Li_2S_6) on both electrode sides for cycling evaluation.

The Li || Cu asymmetric cells were constructed using an 11.13 mm lithium disk paired with a 12.7 mm copper foil separated by a 19.05 mm PP separator. Each cell contained 20 µL electrolyte (with or without Li_2S_6) on both electrode sides for cycling evaluation.

The Li || Li_2S_6 cells were assembled using 12.7 mm carbon paper loaded with 40 µL Li_2S_6 catholyte as the cathode, paired with a 15.8 mm lithium disk as the anode and separated by a 19.05 mm PP separator. An additional 5 µL of Li_2S_6-free electrolyte was added on the anode side to ensure proper wetting.

Li_2S_6 symmetric cells were constructed using two 12.7 mm carbon paper electrodes, each loaded with 20 µL Li_2S_6 solution and separated by a 19.05 mm PP separator.

All cells described above were assembled using standard CR2032 coin cell configuration.

1.8. Spectroscopic characterization

1.8.1. Ex-situ Raman and operando Raman spectroscopy

Ex-situ Raman measurements were performed using a confocal Raman microscope (XploRA Plus, Horiba Scientific) equipped with a 50× objective lens. A 638 nm excitation laser at 0.3 mW was selected to enhance sulfur vibrational sensitivity while minimizing sample heating.

Operando Raman experiments were conducted using a commercial spectroscopy cell (ECC-Opto-10, El-Cell®), following procedures established in our previous work.[1] The cathodes used for operando Raman and operando XAS measurements were prepared using the same slurry formulation as for coin cells, except that the slurry was cast onto Celgard 2400 separator (20 μm thickness). The coated Celgard was punched into 8 mm disks and paired with a 10 mm glass fiber separator and a 9 mm lithium disk as the anode. Finally, to remove fluorescence background, raw Raman spectra were corrected using a fourth-order polynomial baseline subtraction. A fifth-order polynomial smoothing algorithm was then applied to improve signal-to-noise ratio while retaining spectral features, using the LabSpec 6 software suite (Horiba Scientific).

1.8.2. Nuclear Magnetic Resonance (NMR) Spectroscopy

NMR measurements were carried out on a Bruker Avance Neo 500 MHz spectrometer equipped with a broadband BBFO probe and a 60-position SampleCase Plus autosampler. The ^{7}Li spectra were acquired over a chemical shift range from −50 to 50 ppm using 16 scans for signal averaging. Samples were prepared in a coaxial configuration in which the inner insert contained 50 μL of a 1 M LiCl reference solution in D_2O (Fisher Scientific), and the outer tube was filled with 500 μL of the electrolyte or Li_2S_6 sample.

1.8.3. Ex Situ X-ray absorption spectroscopy (XAS)

1.8.3.1. Washing procedure

Because XAS is highly sensitive to elemental composition, the presence of residual LiTFSI on harvested electrodes can obscure sulfur-specific spectral features. Therefore, a controlled washing procedure was implemented to remove surface salt prior to XANES analysis. Electrodes retrieved from SLP cells were sequentially rinsed in three vials, each containing 20 mL DME. To minimize the dissolution of lithium polysulfides (LPSs), each rinse was limited to 10 seconds. This method, previously validated in the literature, has been shown to effectively reduce surface salt contamination without altering the underlying electrode chemistry.[1a, 2]

1.8.3.2. XANES spectroscopy

Standard reference samples, including Li_2S_8, Li_2S_6, Li_2S_4 solutions, and Li_2S powder,were prepared for sulfur K-edge calibration. Li_2S_2 was intentionally excluded due to its susceptibility to oxidation, even under trace oxygen conditions.

XANES measurements for both reference samples and harvested electrodes were conducted at beamline 8-BM (Tender Energy X-ray Absorption Spectroscopy, TES) at the National Synchrotron Light Source II (NSLS-II), Brookhaven National Laboratory. Spectra were collected in a He environment with an energy step size of 1 eV min^{-1} over the range 2427–2462 eV, 0.25 eV min^{-1} for the range 2462–2502 eV, and 1 eV min^{-1} for the range 2502–2592 eV, respectively. Data processing was performed using ATHENA and ARTEMIS.[3]

The pre-edge feature arises primarily from terminal sulfur species, and its intensity varies with the proportion of internal sulfur present in the polysulfide chain. Elemental sulfur (S_8), which lacks terminal groups, shows no pre-edge peak at 2470.5 eV and exhibits a single main peak at 2472.3 eV consistent with its cyclic structure. Long- and mid-chain polysulfides (Li_2S_8–Li_2S_4) share this main peak due to partial retention of internal sulfur motifs. Conversely, Li_2S displays no pre-edge peak and exhibits a main-edge shift from 2472.3 to 2473.1 eV, attributed to increased electron density at the terminal sulfur, which lowers the 1s core-level binding energy and produces a red-shift in the absorption onset.[4] Additional peaks at approximately 2480 and 2482.5 eV correspond to sulfone species (residual $TFSI^-$) and S^{6+} species (sulfate from $TFSI^-$ oxidation).[2]

1.8.4. Ultra-violet visible (UV-vis) Spectroscopy

UV–vis measurements were performed using a Thermo Scientific Evolution 350 spectrophotometer. Calibration solutions of Li_2S_6 were prepared by dilution from 1 mM to 0.1 mM in accordance with the Beer–Lambert law. Time-resolved UV–vis spectra were collected immediately after preparing fully saturated Li_2S_6 solutions in BL and BL+20%. Spectra were acquired every 2 minutes for a total duration of 3 hours in absorbance mode, using a wavelength range of 700–300 nm and a spectral bandwidth of 1 nm.

1.9. Electrochemical characterization

1.9.1. Linear sweep voltammetry (LSV) measurements

The LSV measurements were performed by linear sweep voltammetry, scanning the cells consisting of BL and BL+20% electrolytes soaked in a glass fiber separator and sandwiched between lithium (as the reference electrode) and stainless steel (as the counter and working electrode) electrodes. The potential was swept from the open-circuit voltage (OCV) up to 4.8 V at a scan rate of 0.1 mV s^{-1}.

1.9.2. Ionic conductivity measurement

The ionic conductivities (σ) of the electrolytes were measured using a Metrohm 912 Conductometer with two platinum electrodes. Ionic conductivity was then calculated using:

$\sigma = Condusctance\ (G)\ X\ cell\ constant\ (c)$ c (=L/A) is the cell constant of the conductometer.

1.9.3. Galvanostatic Intermittent Titration Technique (GITT)

GITT measurements were conducted using a Maccor Series 4000M battery testing system. Cells were discharged or charged for 15 minutes at a C/20 rate, followed by a 6 hour rest period to ensure that the system reached thermodynamic equilibrium. The voltage window was set between 1.6 and 2.8 V for all experiments.

1.9.4. Liquid-liquid sulfur reduction reaction (SRR) kinetics evaluation

The kinetics of the liquid–liquid sulfur reduction reaction (SRR) were evaluated using temperature-dependent EIS and distribution of relaxation time (DRT) analysis on Li_2S_6 symmetric cells.

EIS measurements were performed using a potentiostat (VersaStat4, Ametek Inc.) in a frequency range of 0.01 to 10^5 Hz. Temperature control was provided by an environmental chamber (Espec BTZ-133, Advanced Test Equipment Corp.), with measurements collected at 0, 10, 20, 25, 30, 40, 50 and 60 °C. Each temperature step included a 10-minute equilibration period prior to data acquisition.

DRT analysis was carried out using the MATLAB GUI-based DRT tools developed by Ciucci's group.[5] Gaussian discretization was applied with a regularization parameter of 10^{-3} and a FWHM constraint of 0.5. Equivalent circuit models derived from the DRT profiles were used to extract

electrochemical parameters, including the contact resistance (R_{con}) and charge-transfer resistance (R_{CT}). The activation energy associated with the charge-transfer process was determined from the Arrhenius relationship:

$$R_{CT} = ke^{-\frac{E}{RT}}$$

Where R_{CT} represents the charge transfer resistance, k is the pre-exponential factor, R is the molar gas constant (8.314 J mol^{-1} K^{-1}), T is the applied temperature in the EIS measurements, and E is the activation energy.

1.9.5. Lithium polysulfides (LPSs) diffusion evaluation

Cyclic voltammetry (CV) measurements were conducted on Li || Li_2S_6 cells using a VersaStat4 electrochemical workstation (Ametek, Inc.) at a scanning rate of 0.1 mV s^{-1}. The resulting voltammograms, which include two reduction peaks (R_1 and R_2) and one oxidation peak (O), were analyzed using the Randles–Sevcik equation:

$$D^{\frac{1}{2}} = i_P \ / \ (2.69 \times 10^5) n (\alpha n)^{\frac{1}{2}} A C v^{\frac{1}{2}}$$

Where i_p is the peak current (A), A is the electrode area (cm^2), C is the active species concentration (mol cm^{-3}), ν is the scan rate (V s^{-1}), D is the diffusion coefficient (cm^2 s^{-1}), n is the number of the transferred electrons (4 for process R1, 12 for process R2, and 16 for process O according to the apparent reaction pathways),[6] and α is the transfer coefficient (assumed to be 0.5 for reversible processes).

1.9.6. Lithium plating and stripping test

The Li || Li symmetrical cells were cycled at an areal current density of 0.5 mA cm^{-2} and an areal capacity of 0.5 mAh cm^{-2}. After the 1st cycle, EIS were taken for every 11 cycles until the cell finished 100 cycles, followed by a final EIS measurement. A higher current condition of 1 mA cm^{-2} and 1 mAh cm^{-2} was evaluated using the same protocol.

The Li || Cu asymmetrical cells were first discharged at 0.5 mA cm^{-2} for 10 hrs to establish the initial lithium deposition. Subsequent cycling was performed at the same current density and an

areal capacity of 0.5 mAh cm^{-2} for 150 cycles. Higher current conditions of 1 mA cm^{-2} and 1 mAh cm^{-2} was tested under the same protocol. Cells containing Li_2S_6 were initially discharged at 0.1 mA cm^{-2} for LPSs reduction, followed by the same plating–stripping procedure.

Average CE measurements in Li || Cu cells began with an initial plating and stripping process at 0.1 mA cm^{-2} for 5 hrs each. This was followed by a 5-hour plating step at 0.5 or 1 mA cm^{-2}, and then 15 cycles of alternating 1-hour plating and 1-hour stripping. The test concluded with a final stripping step at the same current density until reaching a cut-off voltage of 1 V. Cells containing Li_2S_6 underwent a preliminary discharge at 0.1 mA cm^{-2} for LPSs reduction, followed by the same CE testing protocol.

1.9.7. Shuttle current test

Shuttle current measurements were performed using standard coin cells. Cells were first discharged galvanostatically to 2.10 V, followed by a sequence of potentiostatic charging steps. The voltage was incrementally increased from 2.15 V to 2.40 V in 0.01–0.02 V steps (2.15, 2.20, 2.21, 2.22, 2.23, 2.24, 2.25, 2.26, 2.27, 2.28, 2.29, 2.30, 2.31, 2.32, 2.33, 2.34, 2.35, 2.36, 2.37, 2.38, 2.39, and 2.40 V).

At each applied voltage, the shuttle current was determined from the steady-state current reached under potentiostatic control.

2. Supplementary figures

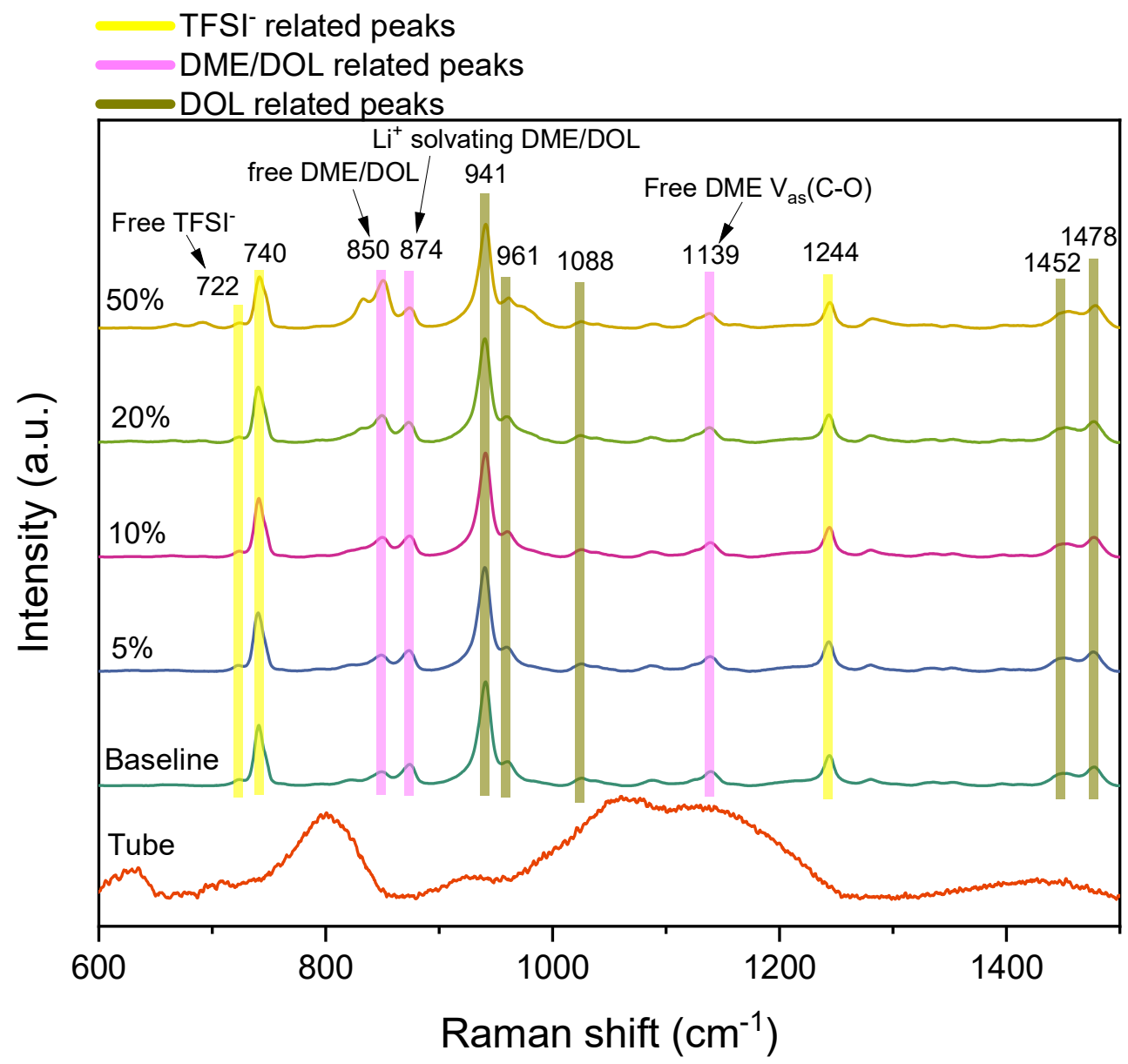

Figure S1 Full-range Raman spectra of 1200ET across an expanded set of concentration gradients, with the background spectrum of the empty tube included for reference.

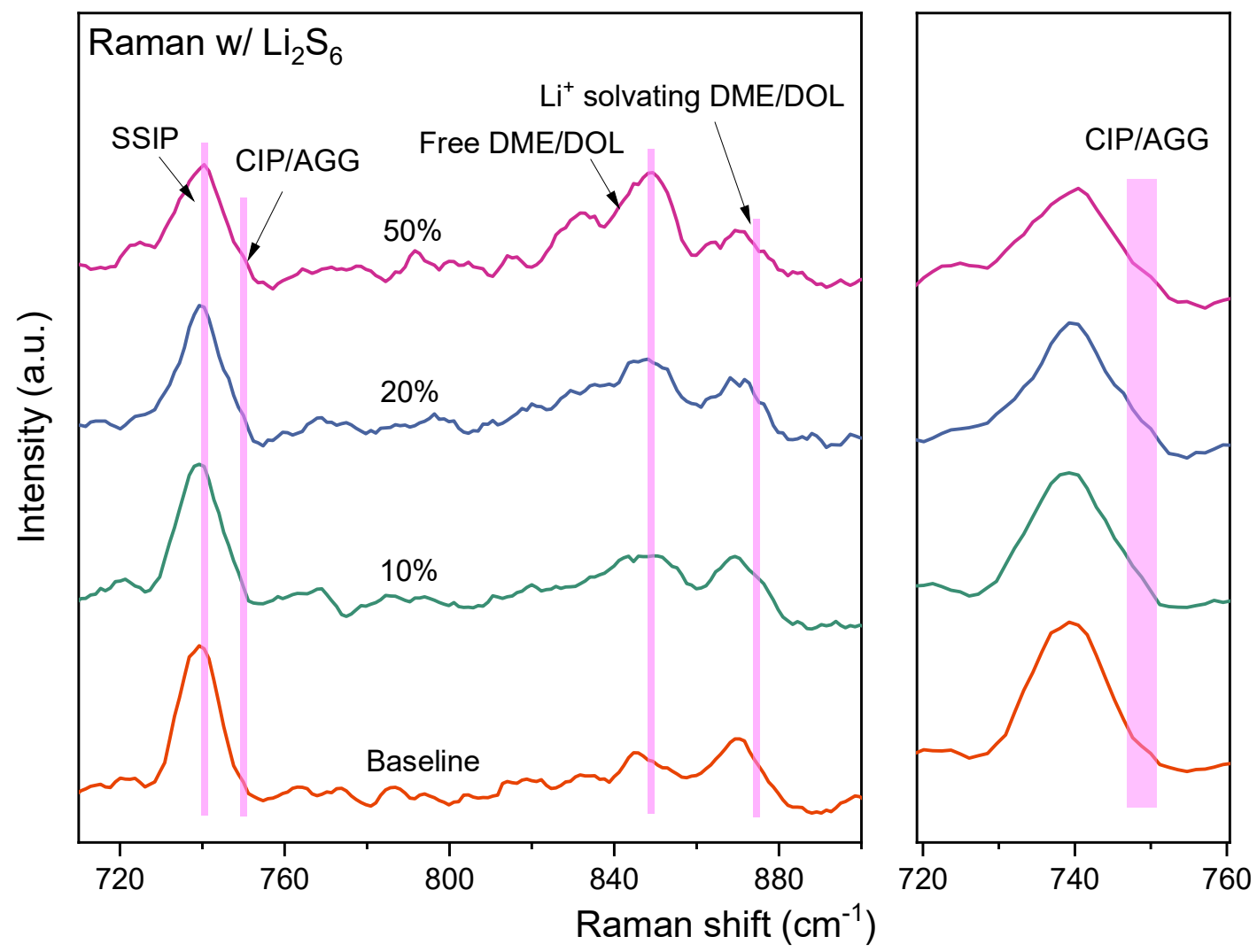

Figure S2 Narrow-range Raman spectra of Li_2S_6 highlighting the characteristic features associated with the CIP / AGG composition

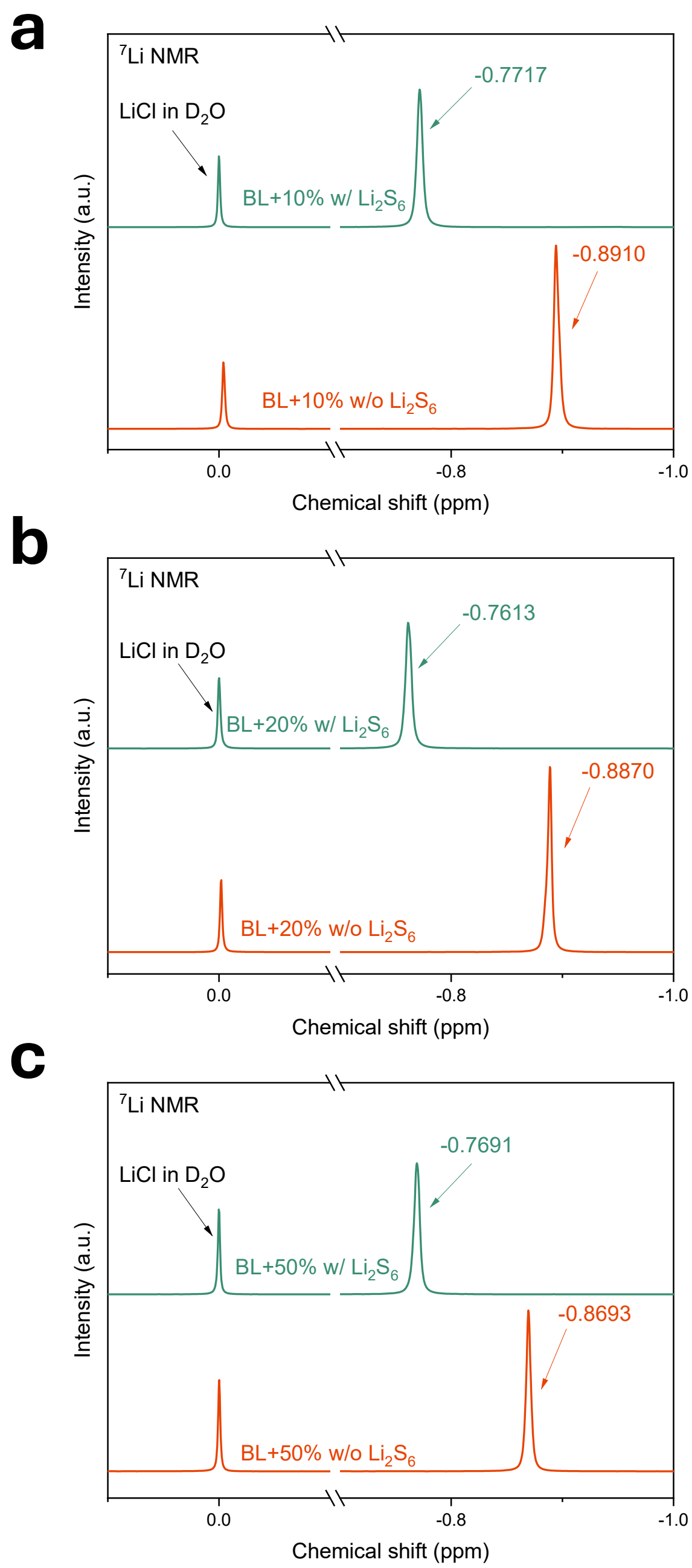

Figure S3 ^{7}Li NMR spectra comparing samples with and without Li_2S_6 for (a) BL+10%, (b) BL+20%, and (c) BL+50%.

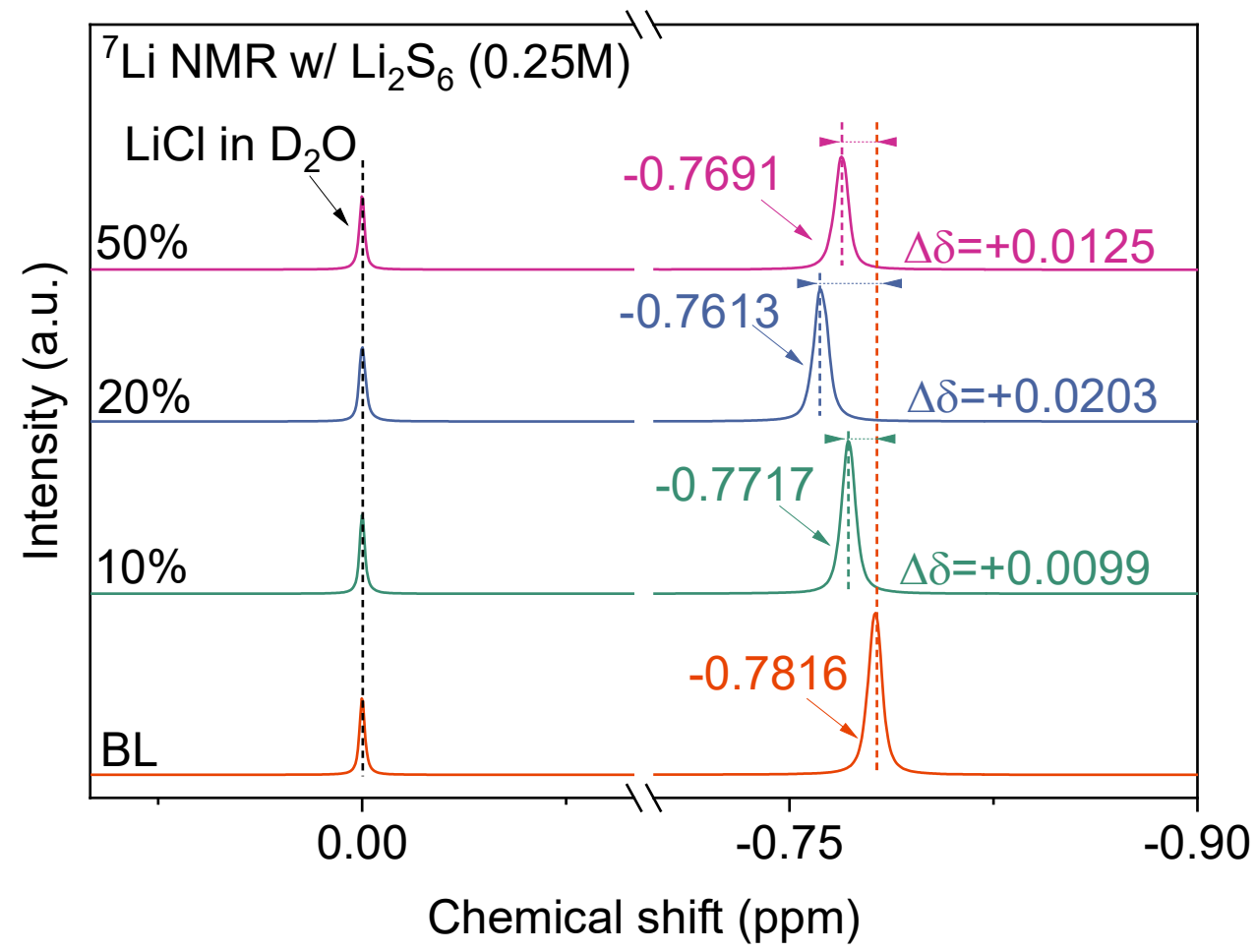

Figure S4 ^{7}Li NMR of BL containing different amounts of 1200ET with Li_2S_6.

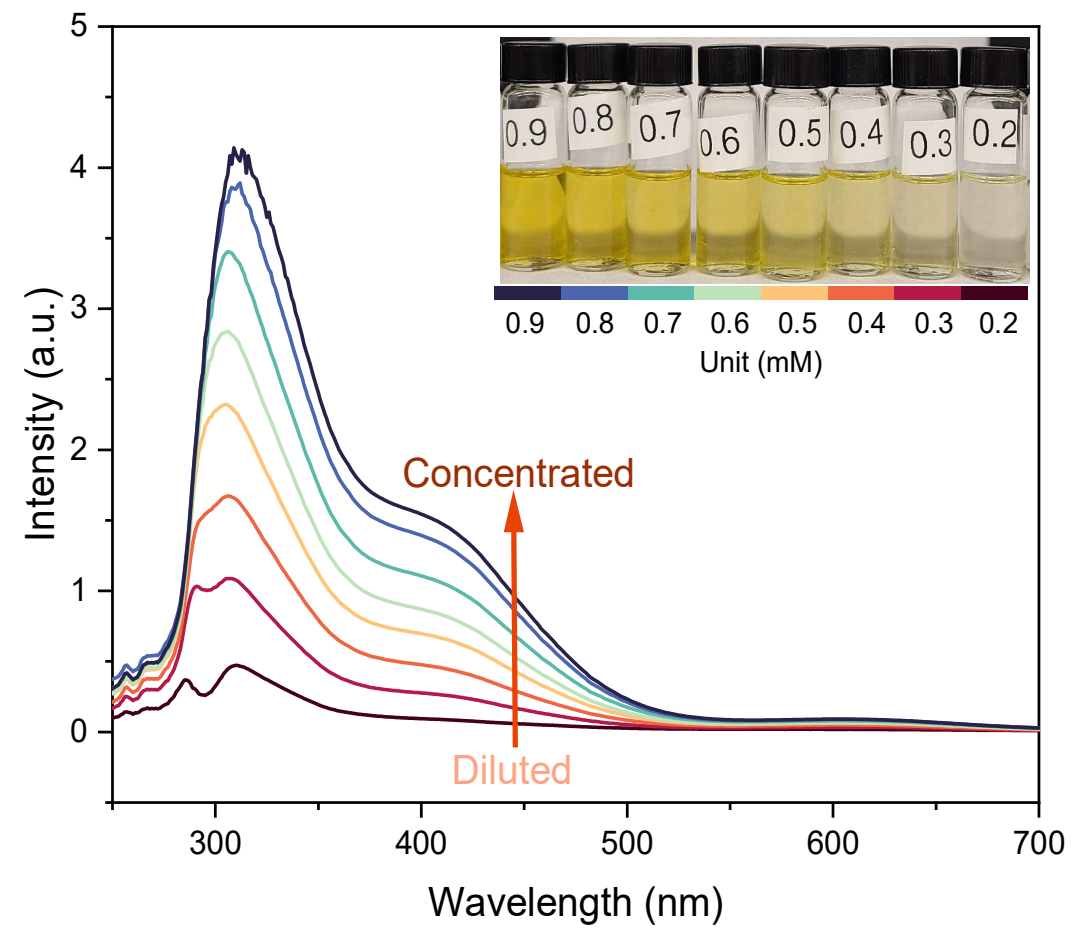

Figure S5 UV–vis calibration spectra of Li_2S_6 solutions spanning diluted to concentrated compositions.

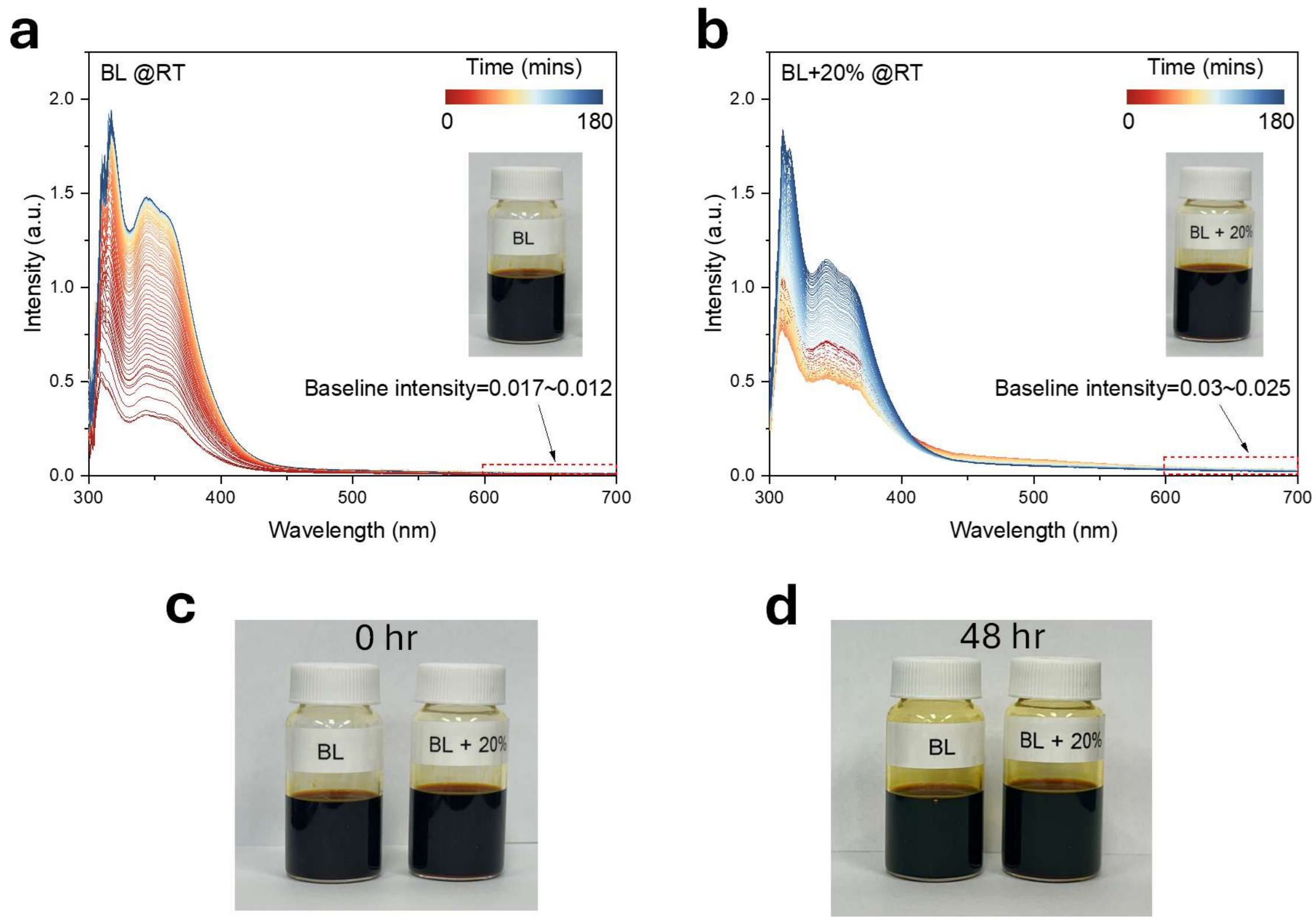

Figure S6 (a-b) Time-dependent UV–vis spectra of saturated Li_2S_6 solutions over 3 hours for (a) BL and (b) BL+20%. (c-d) Photographic records of the color change in BL and BL+20% from (c) 0 hr to (d) 48 hrs.

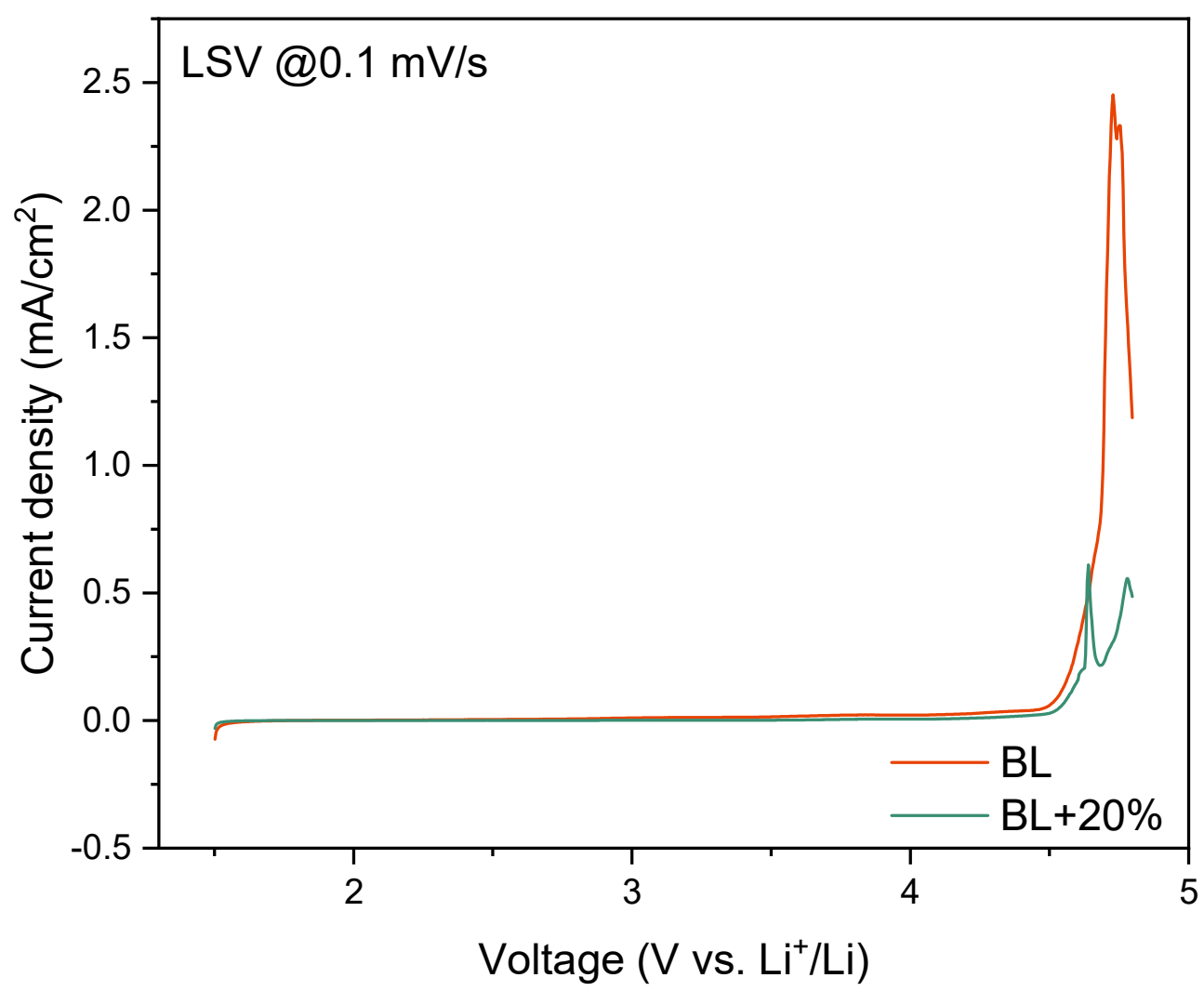

Figure S7 Linear sweep voltammetry (LSV) curves of BL and BL+20% recorded at 0.1 mV s^{-1} up to 4.8 V.

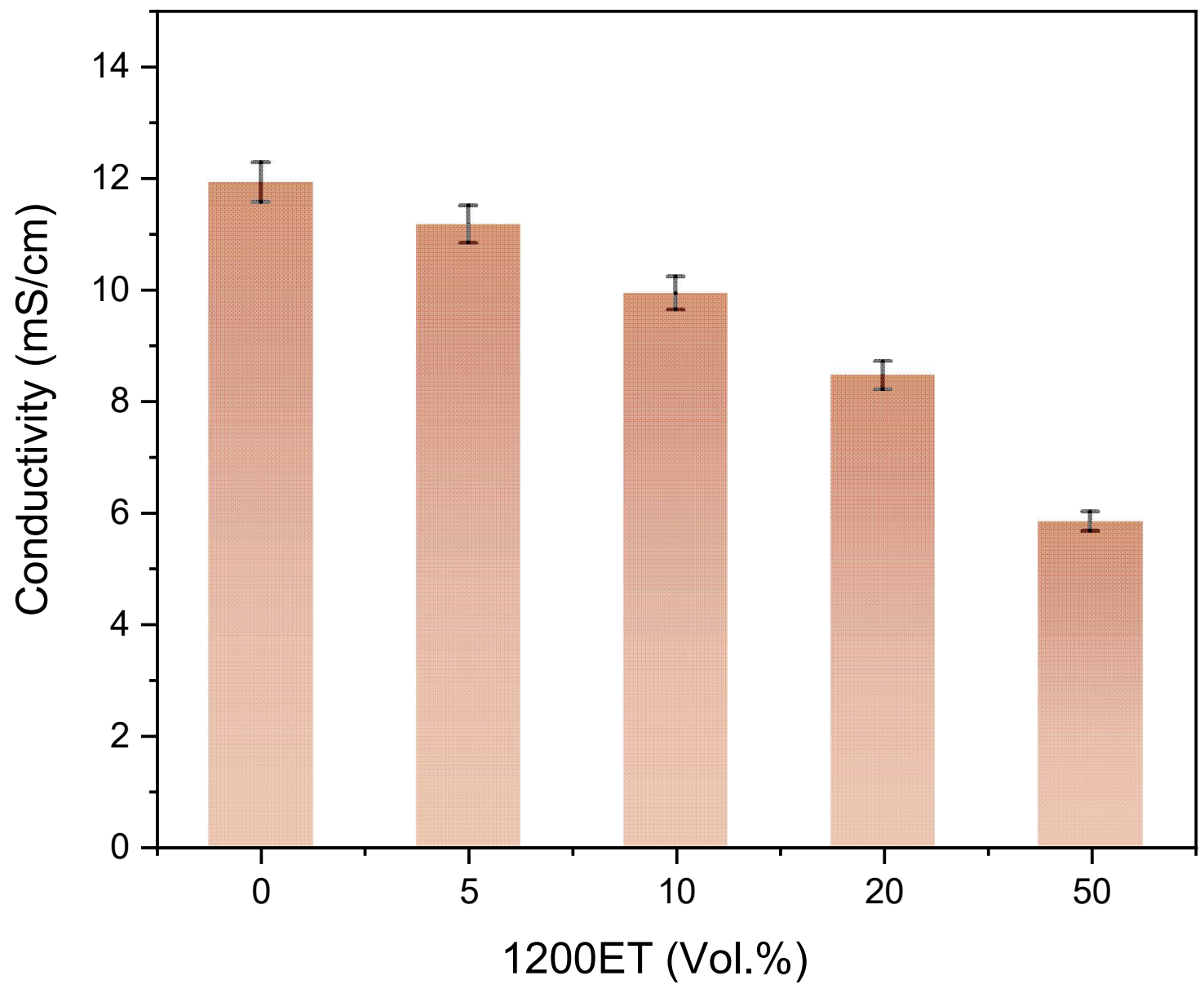

Figure S8 Ionic conductivity of electrolytes containing different volume ratios of 1200ET.

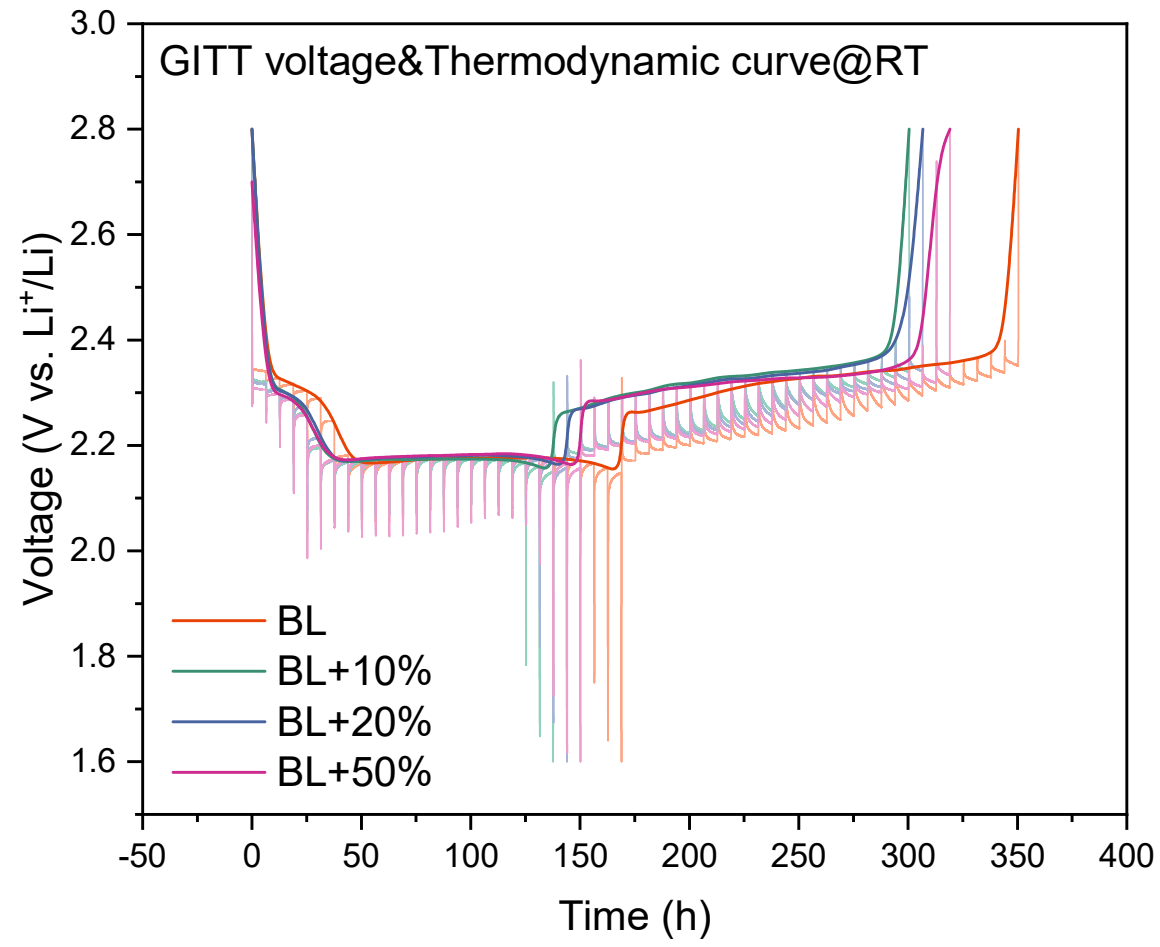

Figure S9 GITT-derived thermodynamic voltage profiles during the full discharge and charge processes for the four electrolytes.

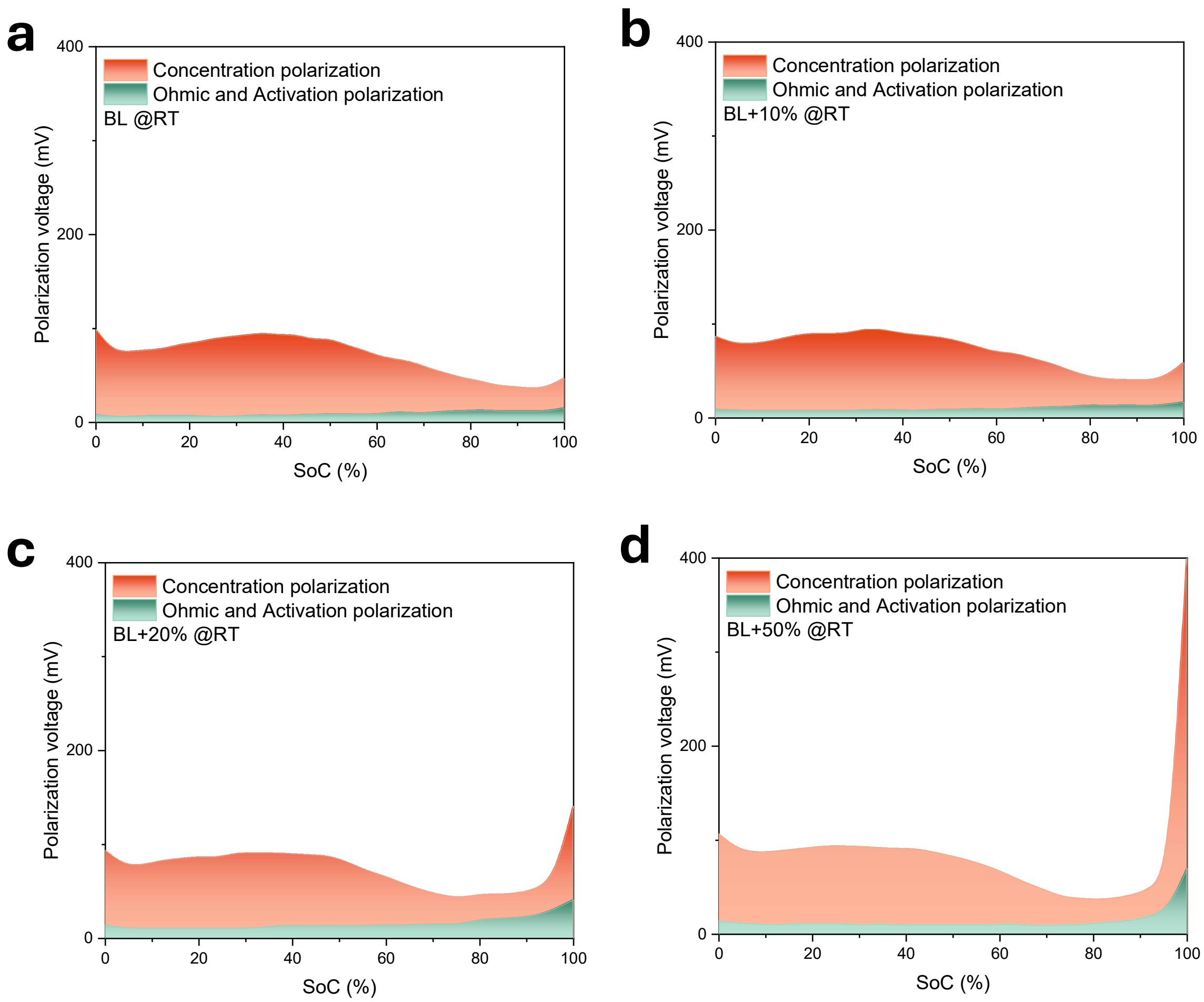

Figure S10 (a-d) Specific polarization voltage components plotted against SoC during the charge process (a) BL, (b) BL+10%, (c) BL+20%, and (d) BL+50%.

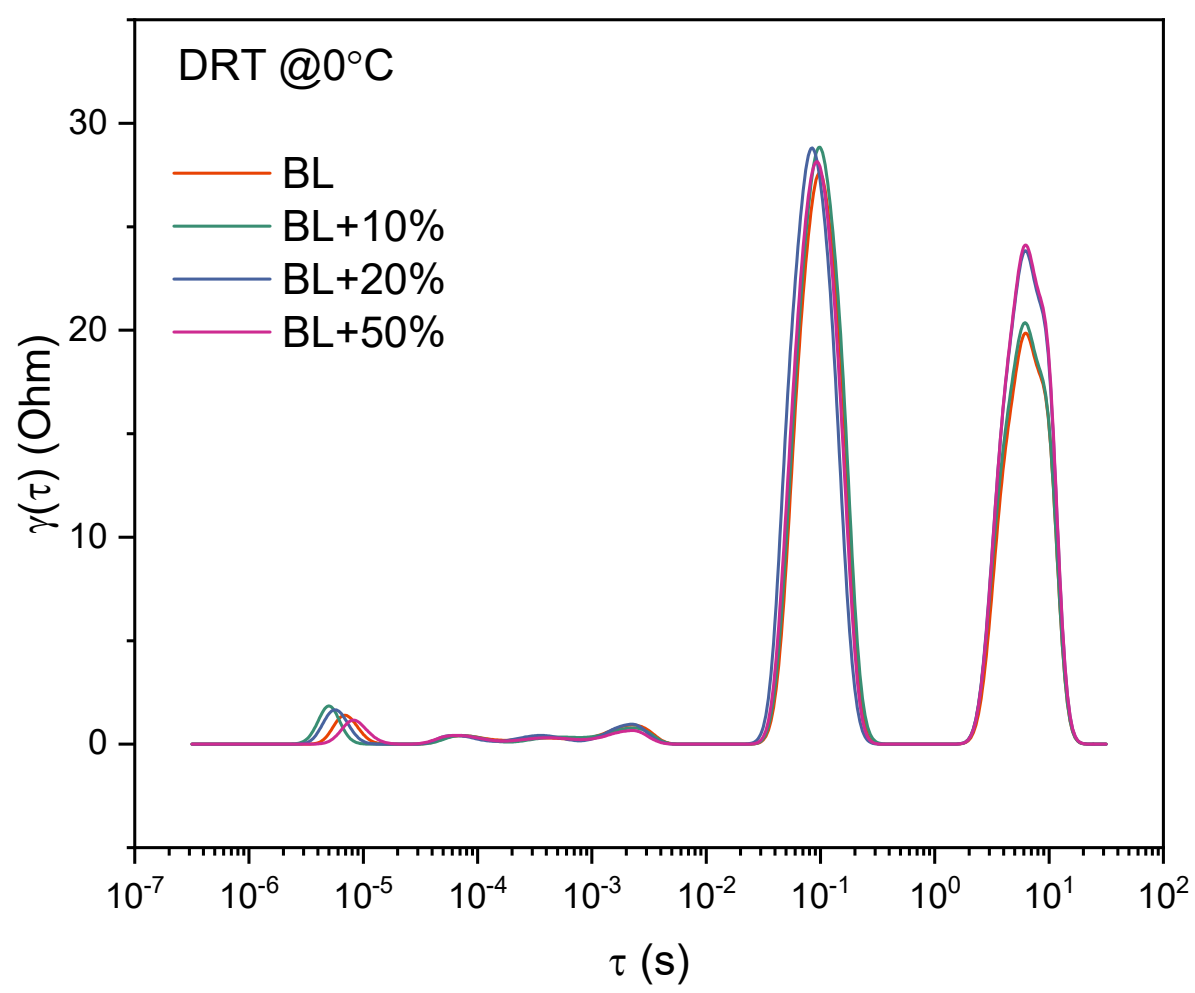

Figure S11 DRT spectra of Li_2S_6 symmetric cell at 0 °C for four different electrolytes.

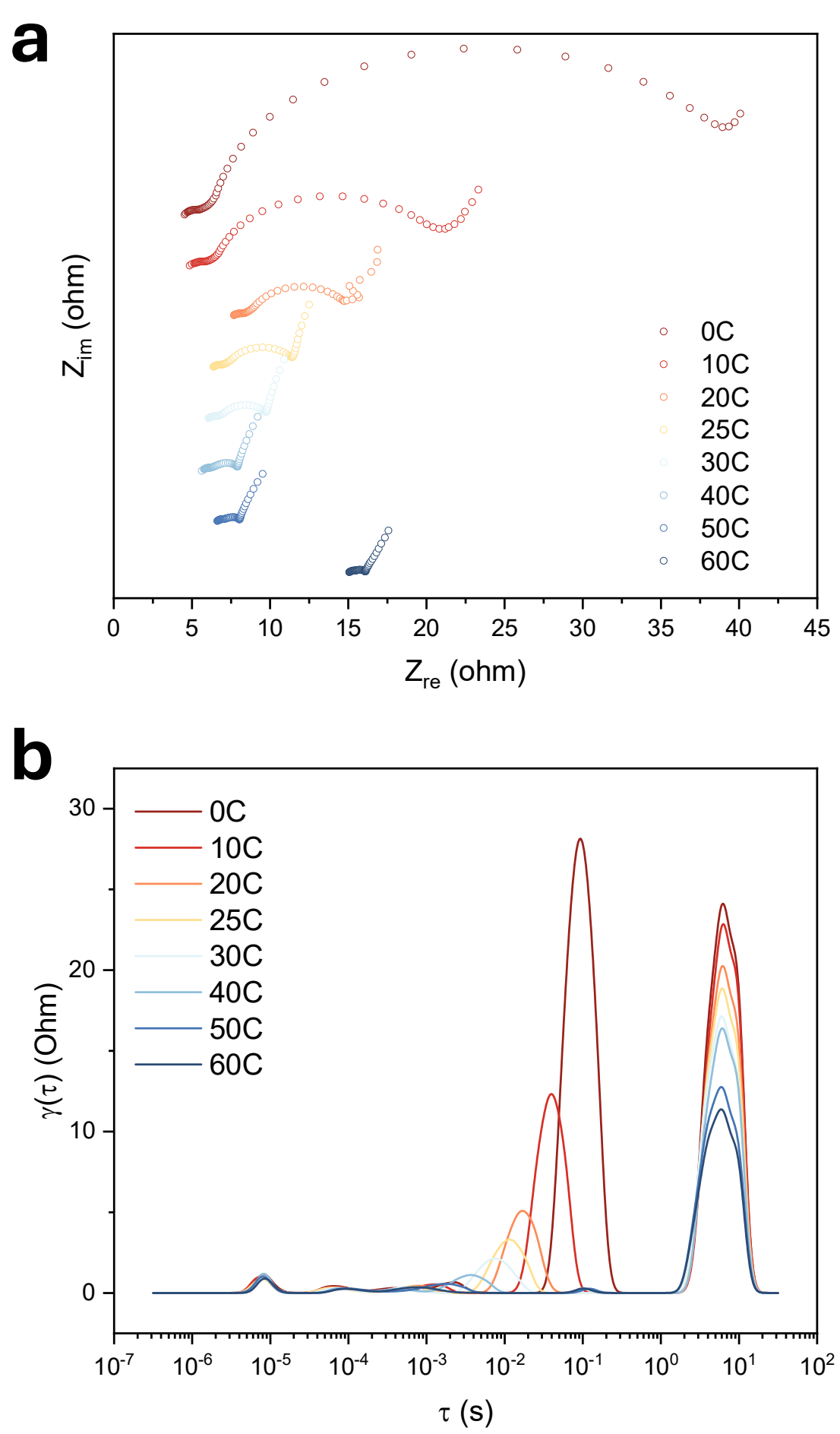

Figure S12 (a) EIS measurements of BL at temperatures ranging from 0 to 60 °C and (b) the corresponding DRT spectra obtained from Li_2S_6 symmetric cells.

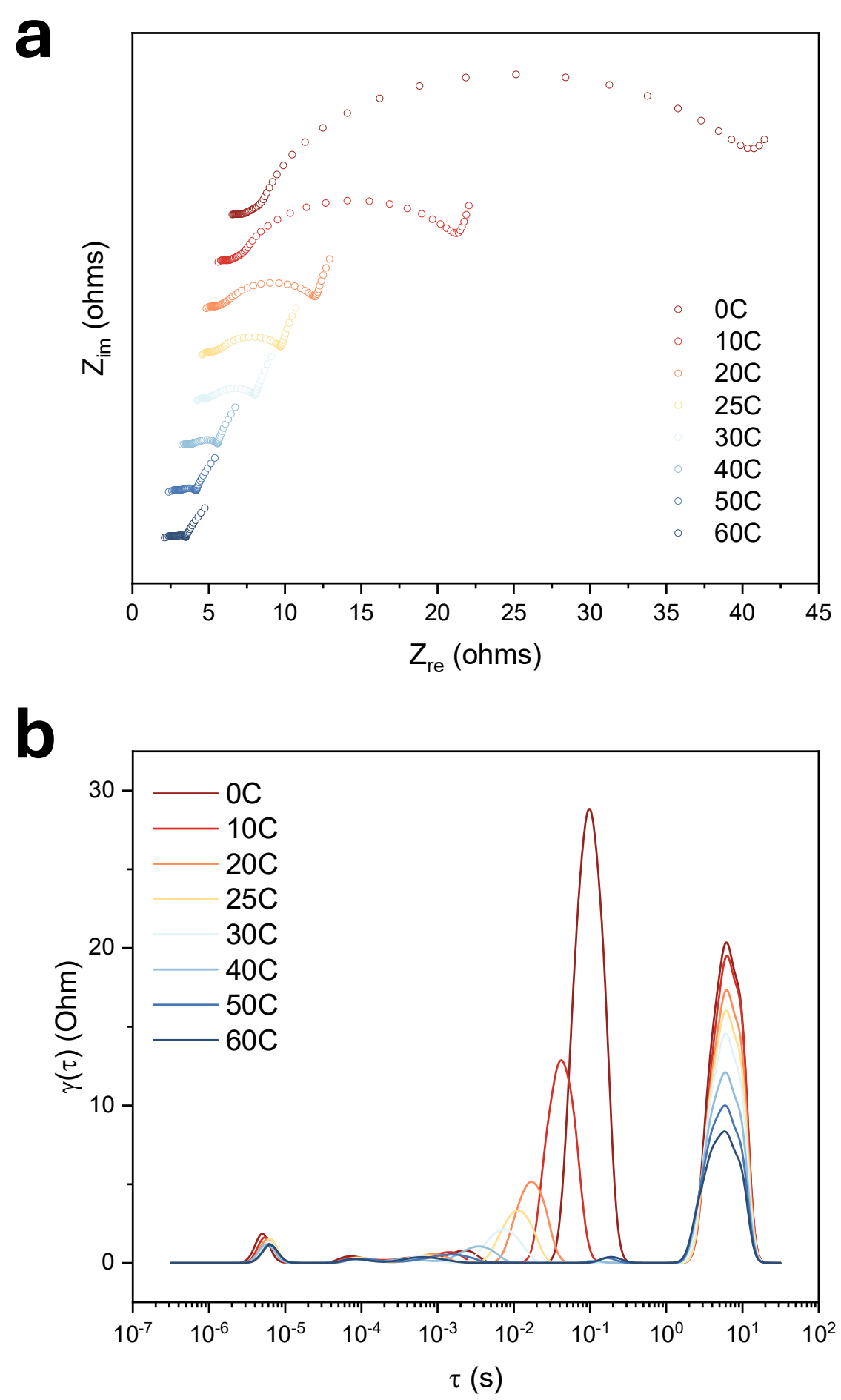

Figure S13 (a) EIS measurements of BL+10% at temperatures ranging from 0 to 60 °C and (b) the corresponding DRT spectra obtained from Li_2S_6 symmetric cells.

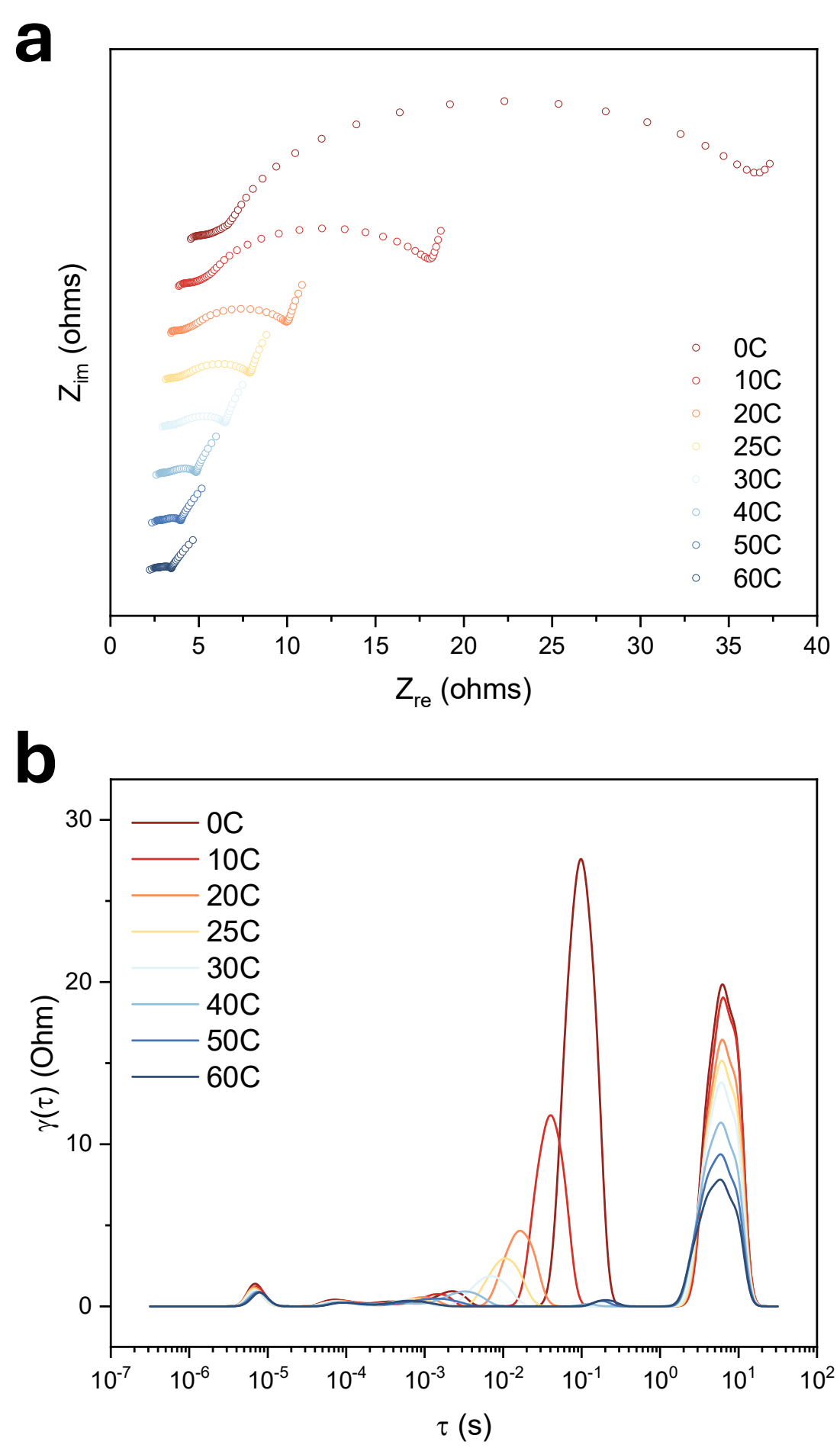

Figure S14 (a) EIS measurements of BL+20% at temperatures ranging from 0 to 60 ℃ and (b) the corresponding DRT spectra obtained from Li_2S_6 symmetric cells.

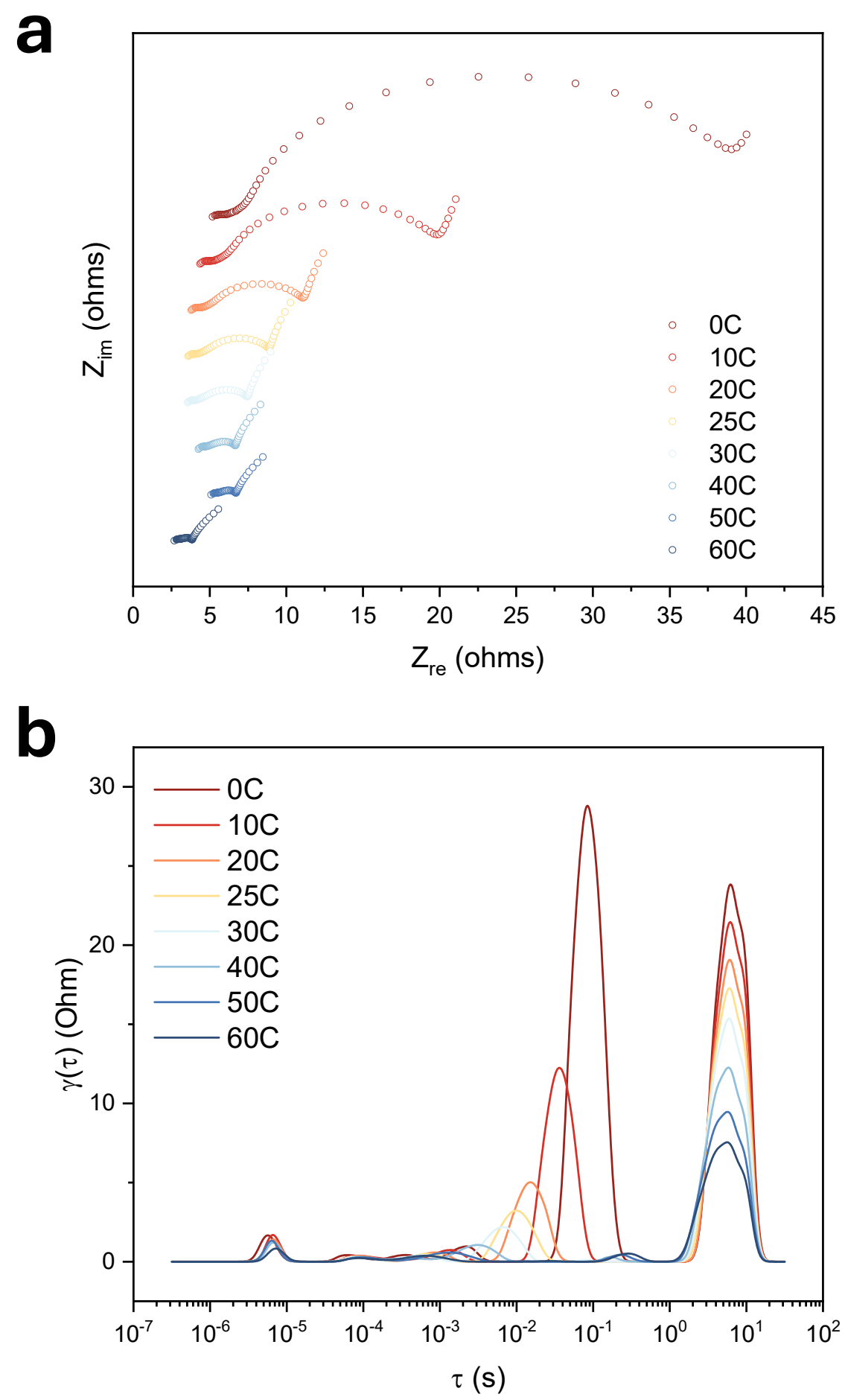

Figure S15 (a) EIS measurements of BL+50% at temperatures ranging from 0 to 60 °C and (b) the corresponding DRT spectra obtained from Li_2S_6 symmetric cells.

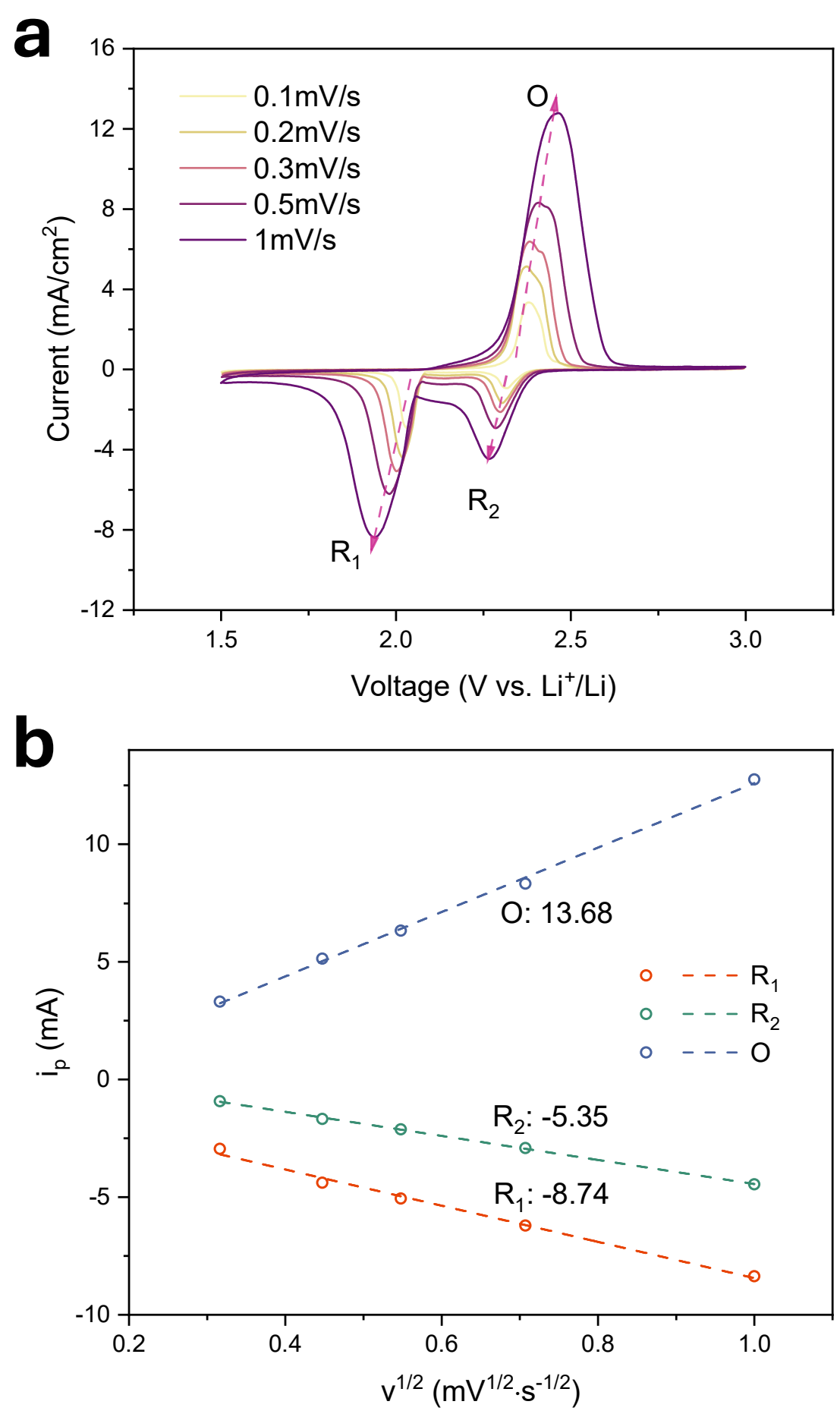

Figure S16 (a) CV curves at various scan rates showing two reduction peaks (R_1 and R_2) and one oxidation peak (O), and (b) the corresponding Randles–Sevcik analysis of peaks R_1, R_2, and O for BL+10%.

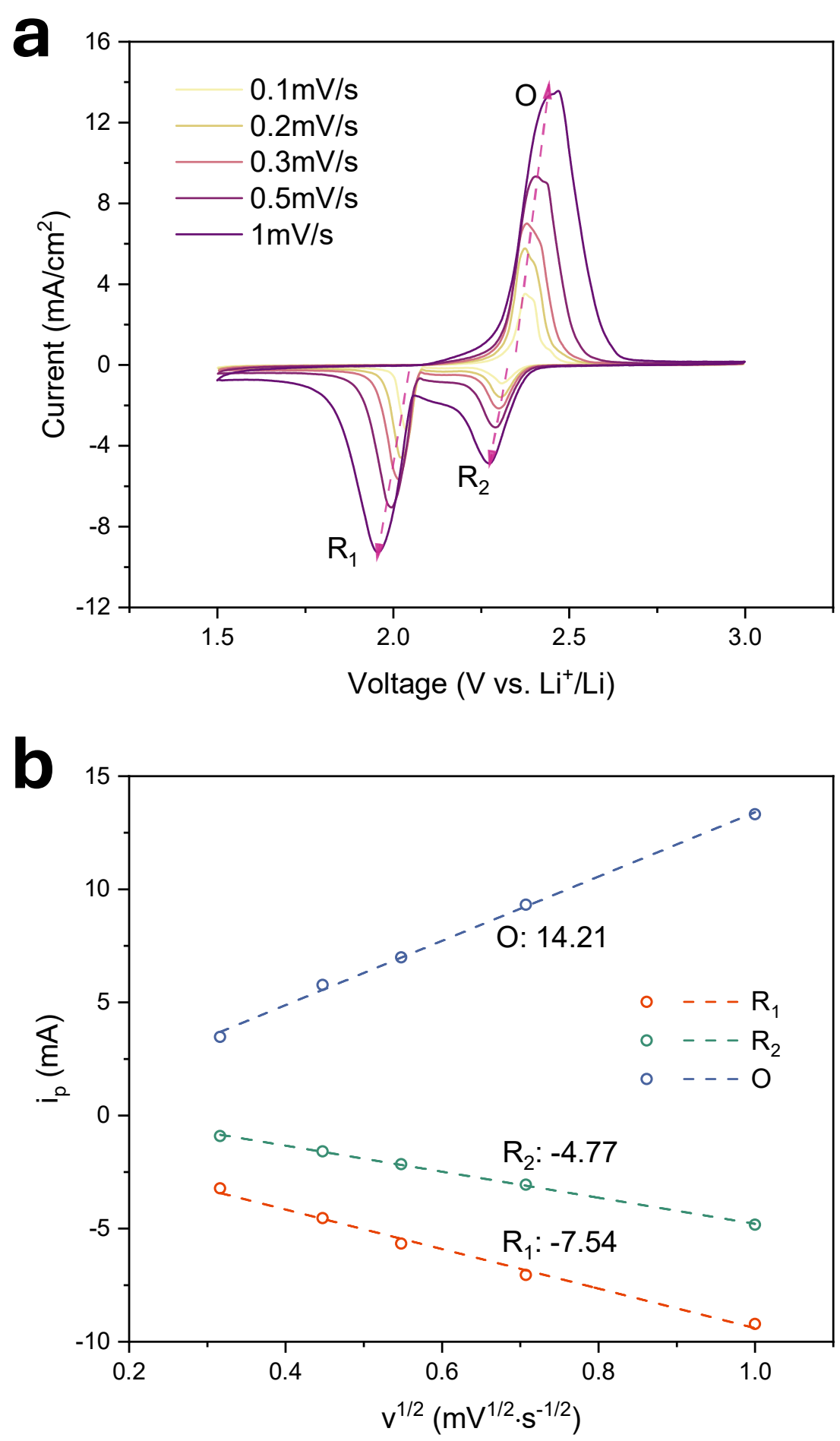

Figure S17 (a) CV curves at various scan rates showing two reduction peaks (R_1 and R_2) and one oxidation peak (O), and (b) the corresponding Randles–Sevcik analysis of peaks R_1, R_2, and O for BL+50%.

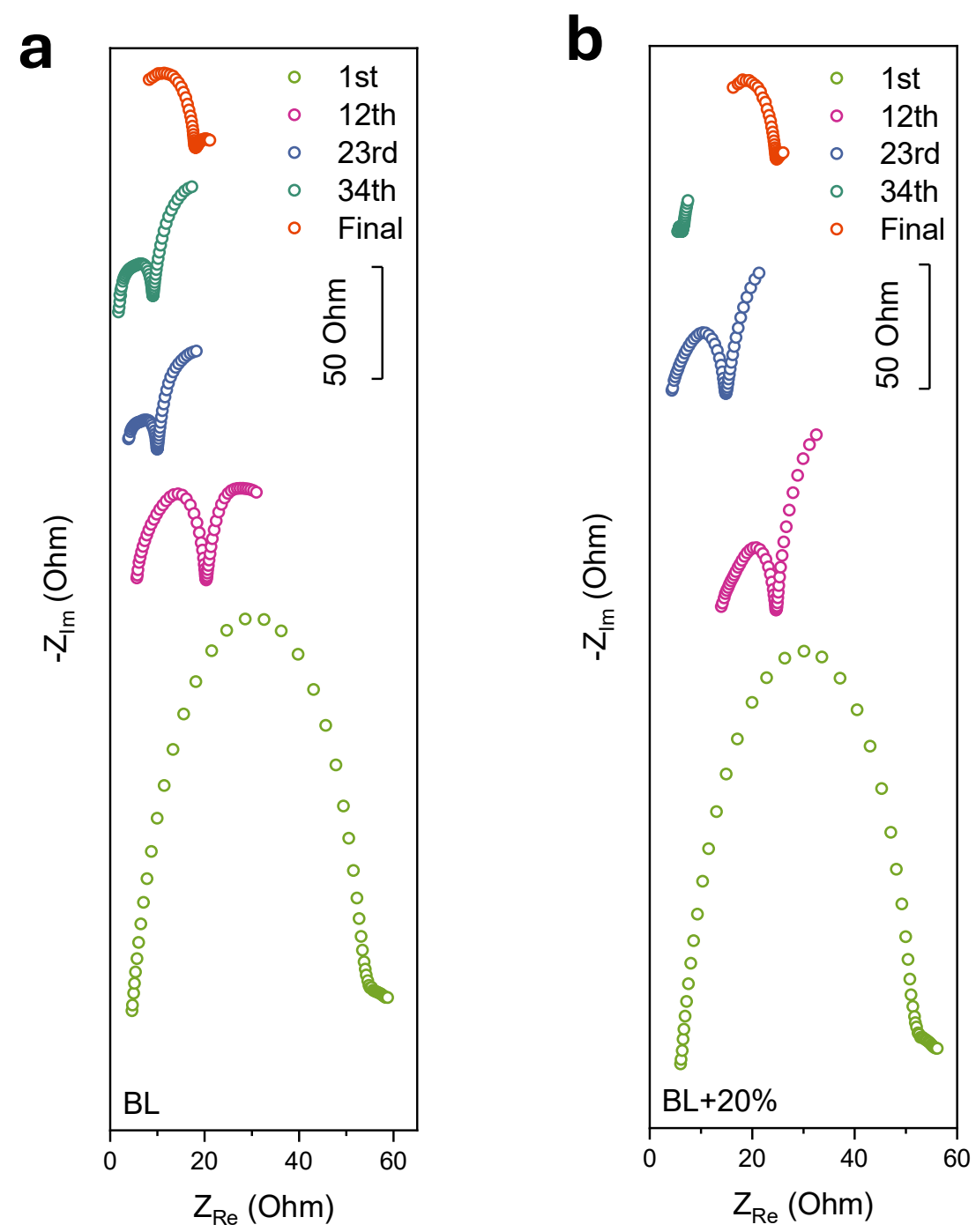

Figure S18 EIS spectra of Li || Li symmetric cells without Li_2S_6, cycled at 0.5 mA cm^{-2} and 0.5 mAh cm^{-2} from cycle 1 to the final cycle for (a) BL, and (b) BL+20%.

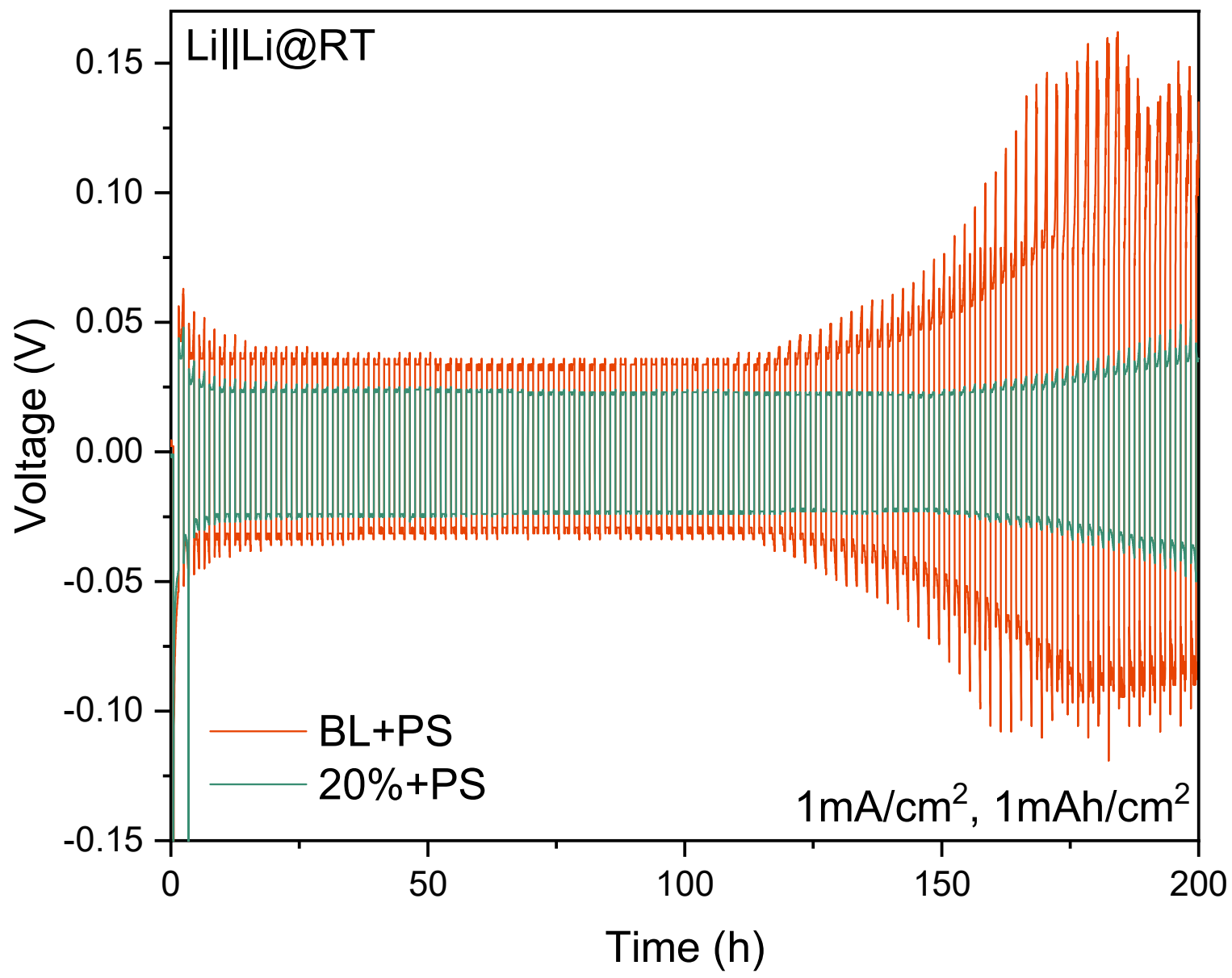

Figure S19 Voltage profile of Li || Li symmetric cells without Li_2S_6, cycled at 1 mA cm^{-2} and 1 mAh cm^{-2}.

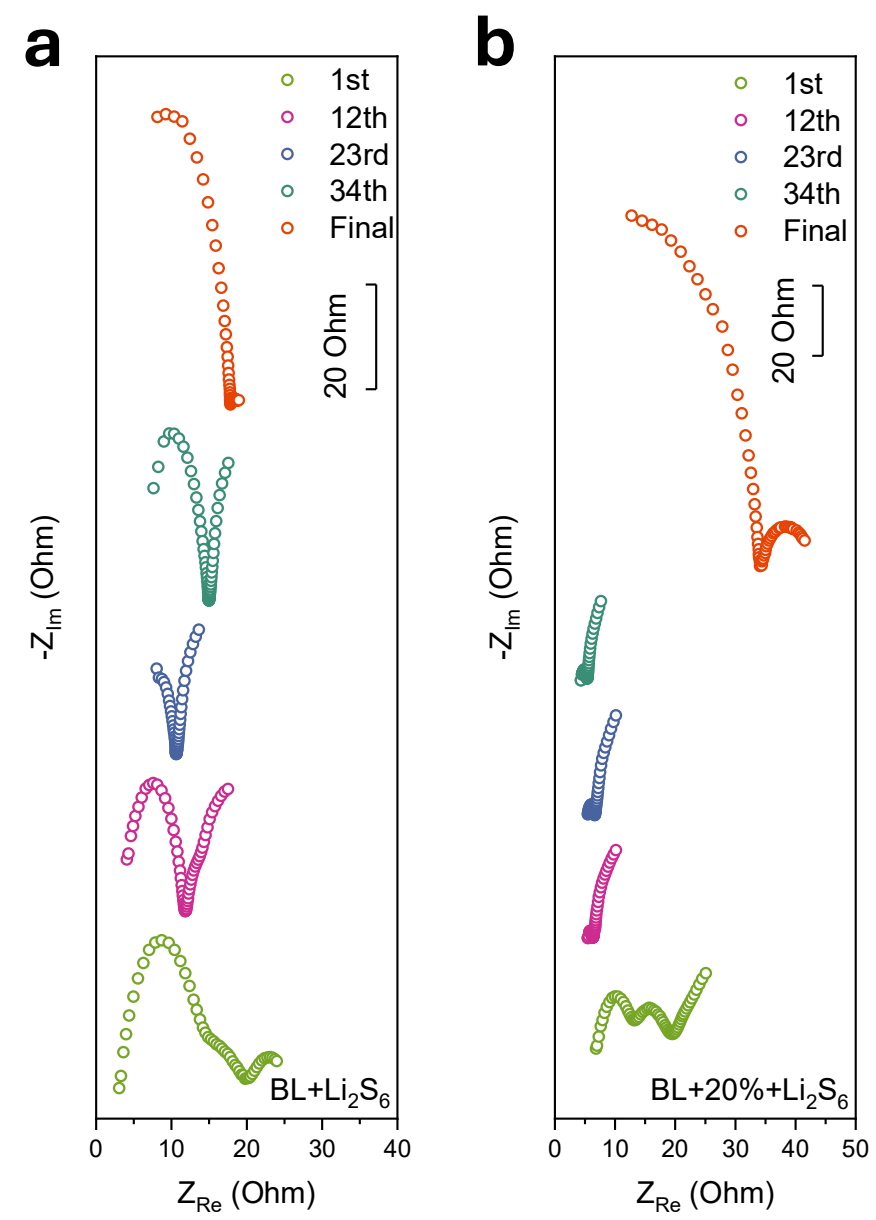

Figure S20 EIS spectra of Li || Li symmetric cells with Li_2S_6, cycled at 1 mA cm^{-2} and 1 mAh cm^{-2} from cycle 1 to the final cycle for (a) BL, and (b) BL+20%.

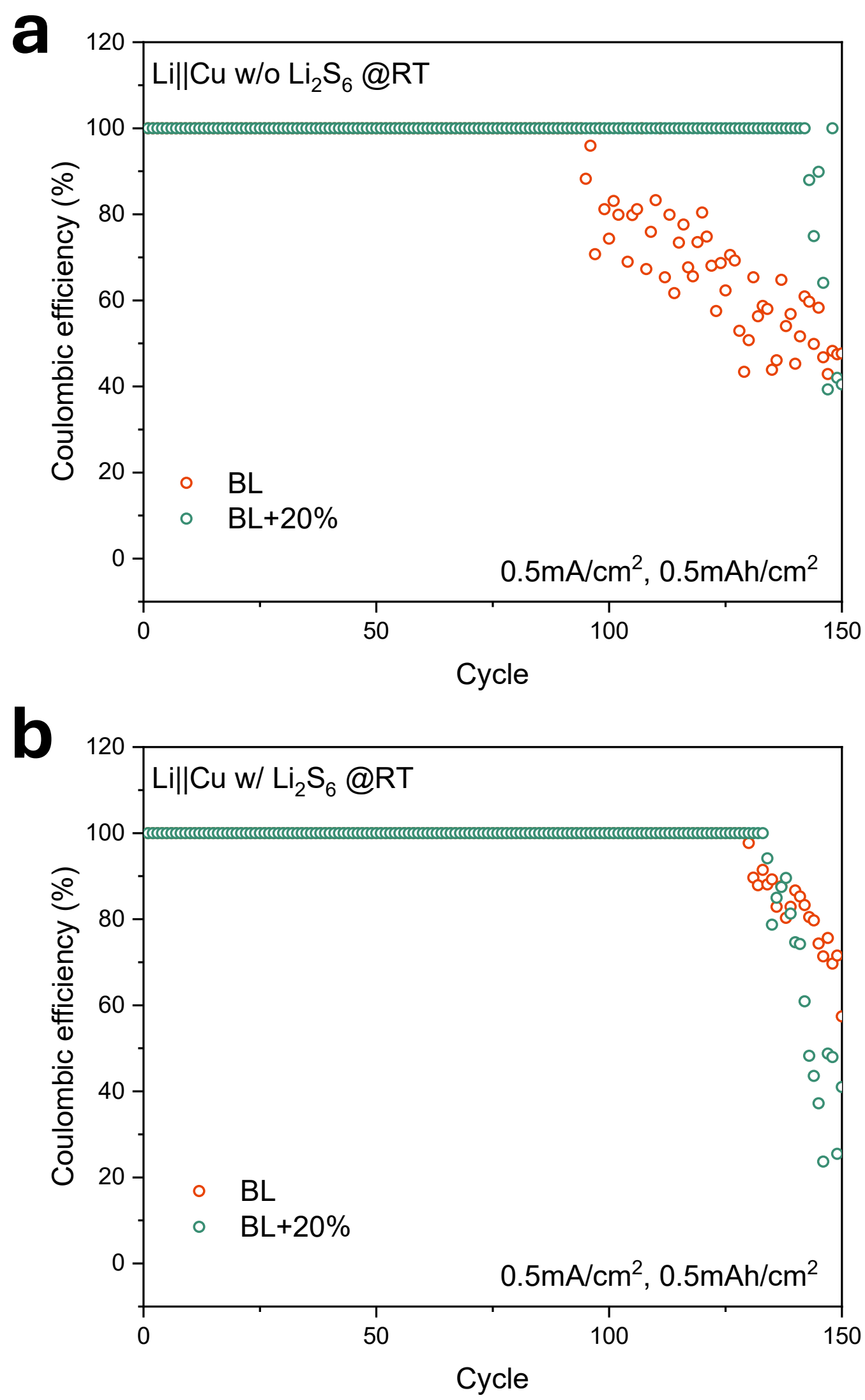

Figure S21 (a-b) Coulombic efficiency evaluation in Li || Cu cells cycled at 1 mA cm^{-2} and 1 mAh cm^{-2} for BL and BL+20% under (a) without Li_2S_6 and (b) with Li_2S_6.

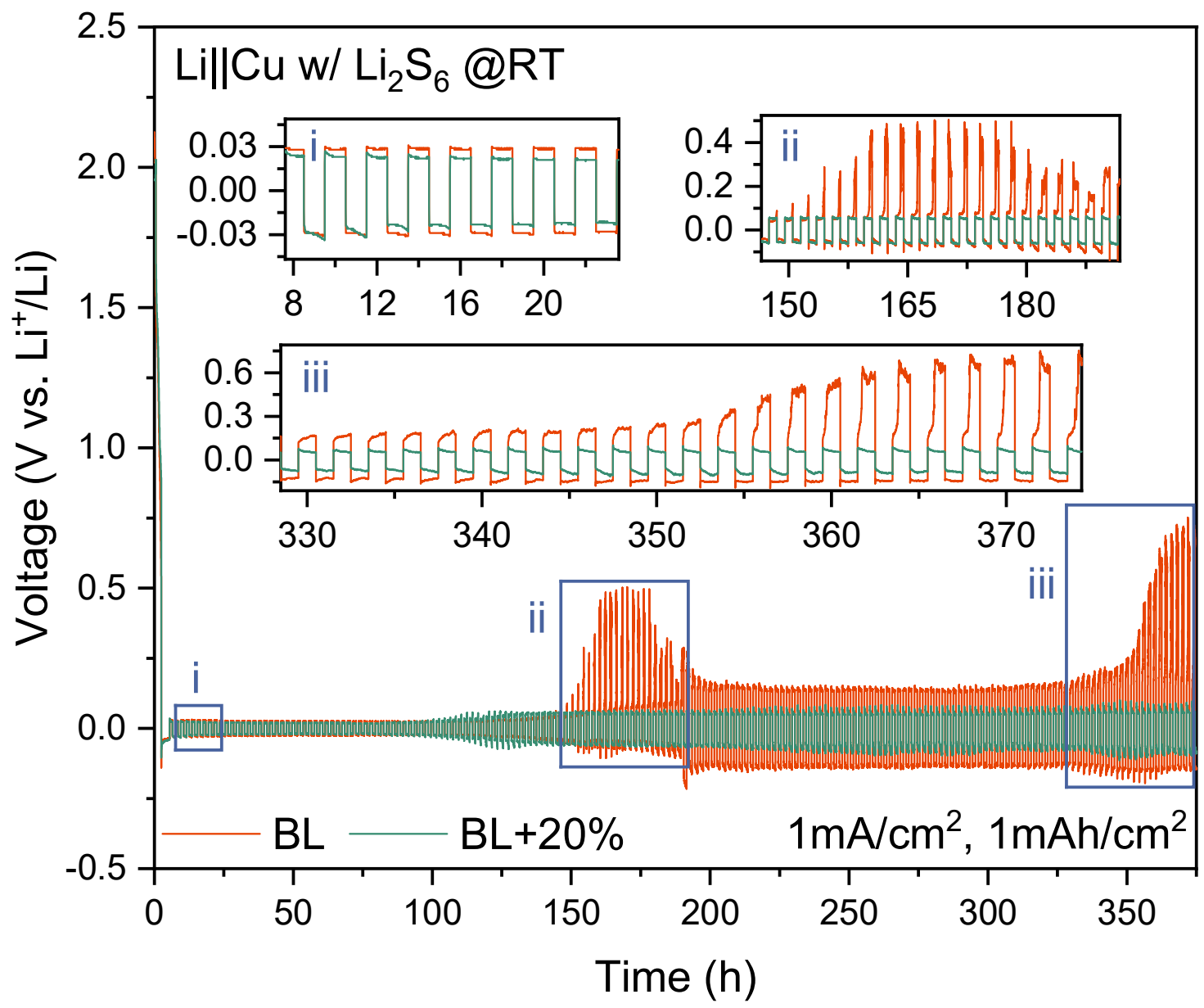

Figure S22 Voltage profiles of Li || Cu asymmetric cells in BL and BL+20% with Li_2S_6, cycled at 1 mA cm^{-2} and 1 mAh cm^{-2}, with enlarged insets highlighting selected regions.

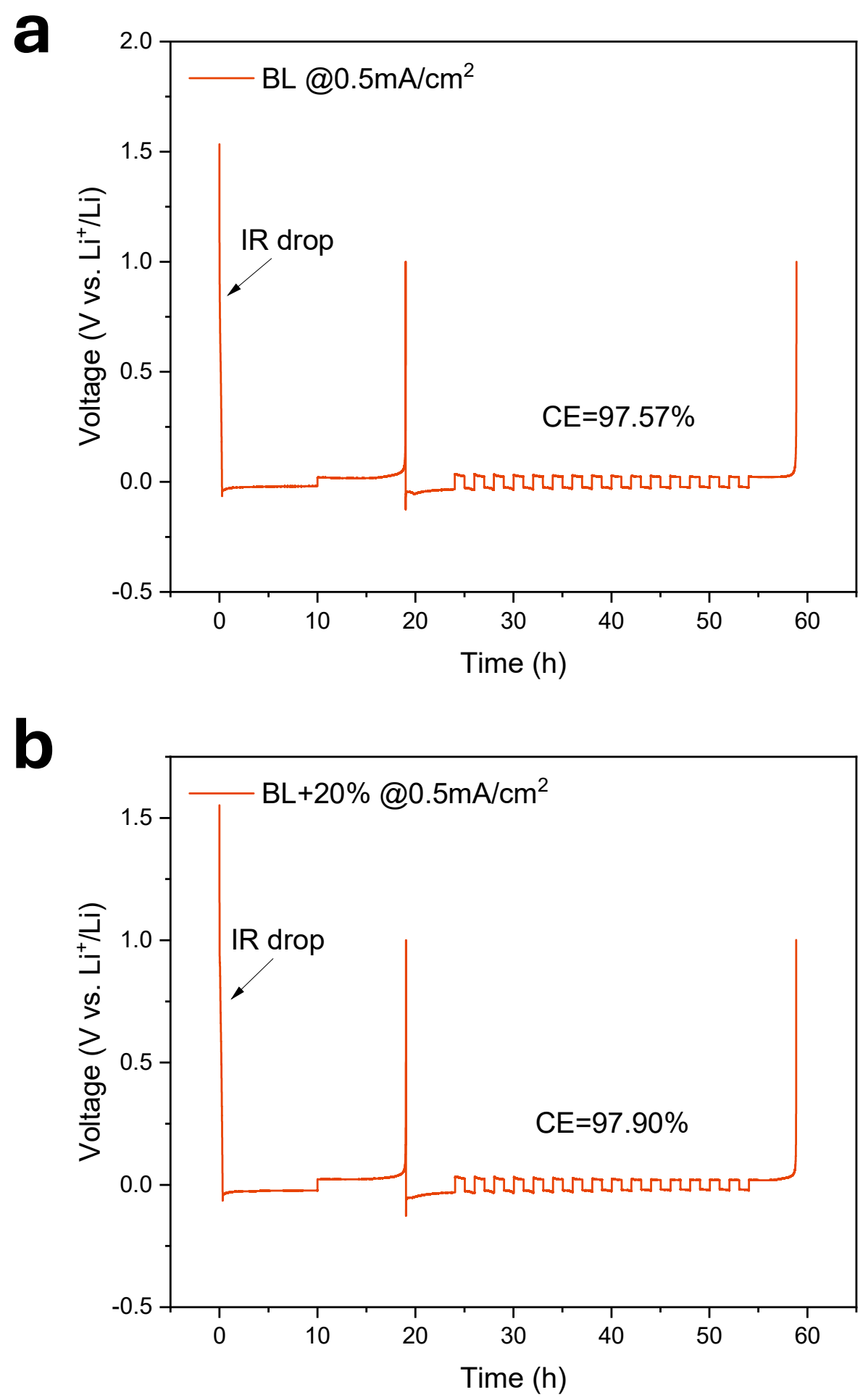

Figure S23 (a-b) Average CE measurements without Li_2S_6 at 0.5 mA cm^{-2} using a modified testing protocol for (a) BL and (b) BL+20%.

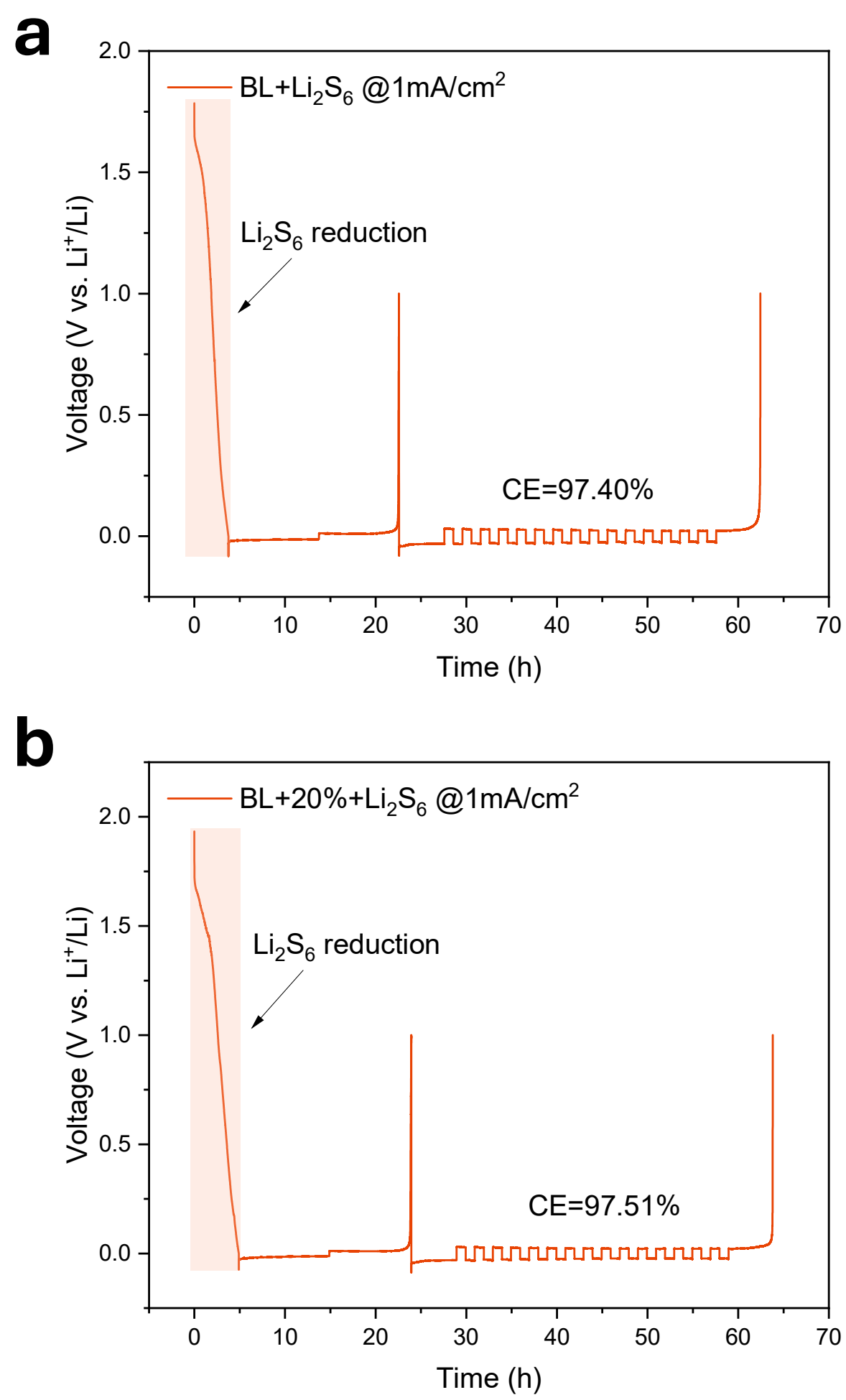

Figure S24 (a-b) Average CE measurements with Li_2S_6 at 1 mA cm^{-2} using a modified testing protocol for (a) BL and (b) BL+20%.

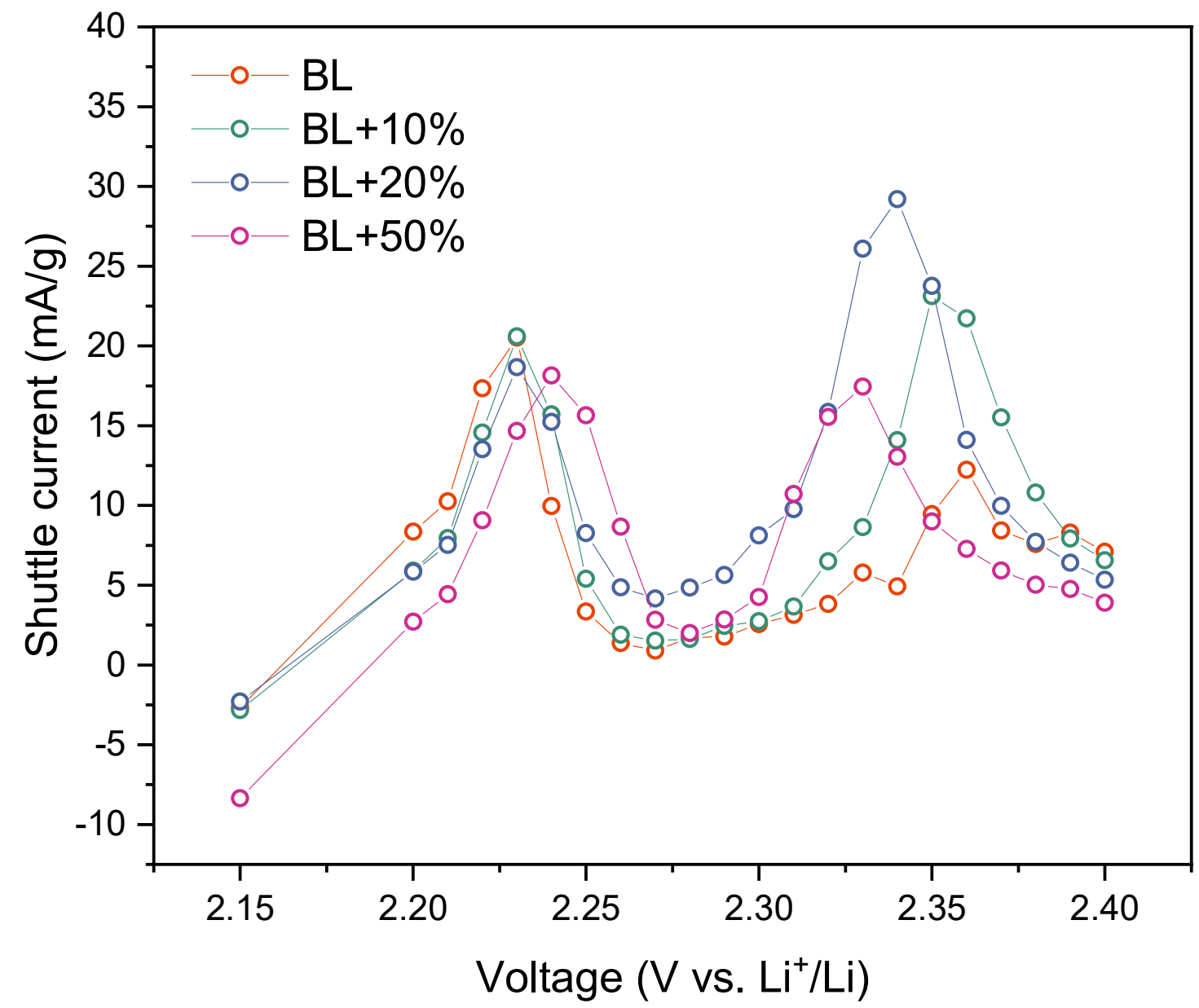

Figure S25 Shuttle current measurements of the four electrolytes under steady-state conditions at each applied potentiostatic voltage.

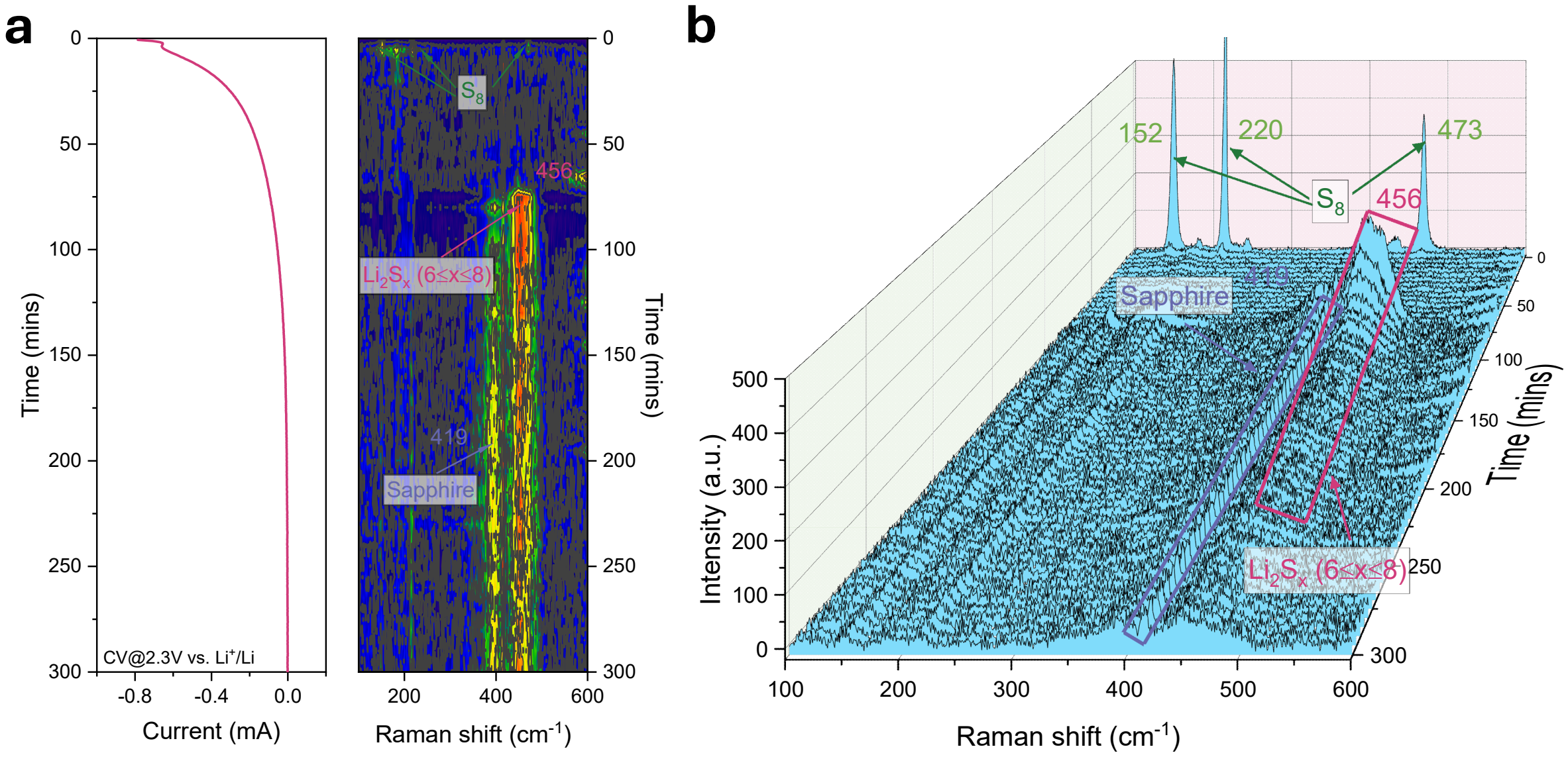

Figure S26 (a) Operando Raman spectra during the potentiostatic hold at 2.3 V, shown together with the corresponding intensity heatmap and voltage profile, and (b) the specific Raman spectra extracted from the operando dataset.

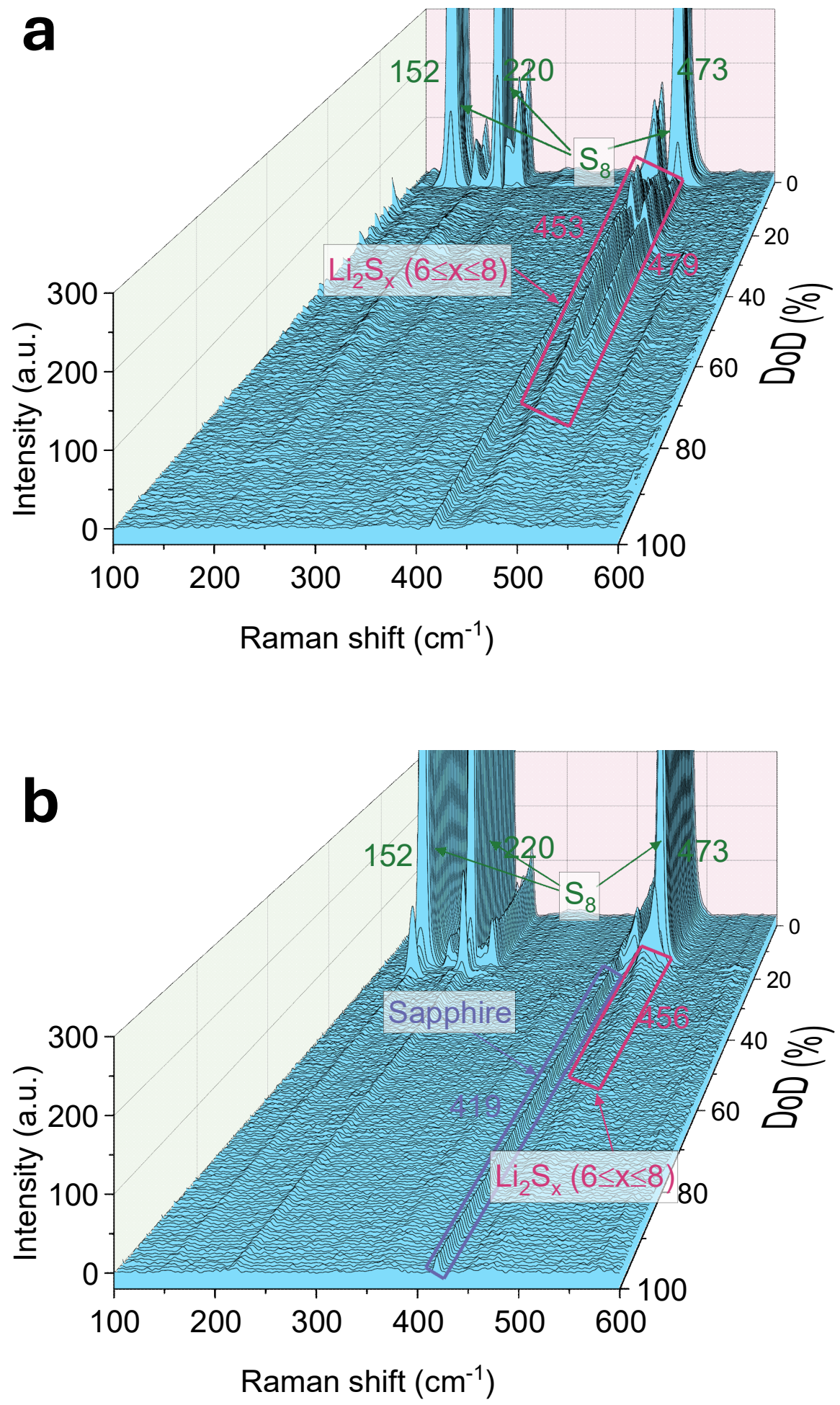

Figure S27 (a-b) Operando Raman spectra at a galvanostatic discharge of C/20 for (a) BL and (b) BL+20%.

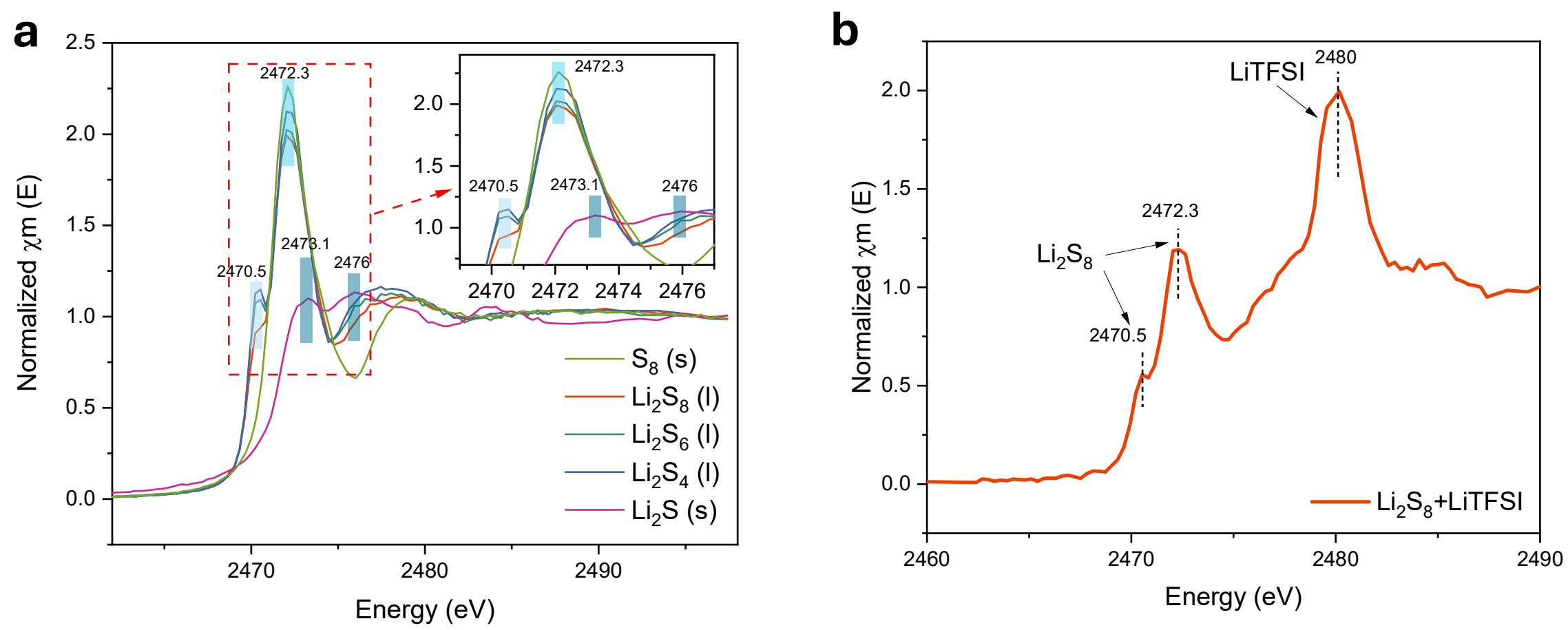

Figure S28 XANES spectra at the sulfur K-edge for (a) standard reference compounds including S_8, Li_2S_8, Li_2S_6, Li_2S_4, and Li_2S, and (b) a Li_2S_8 solution containing LiTFSI.

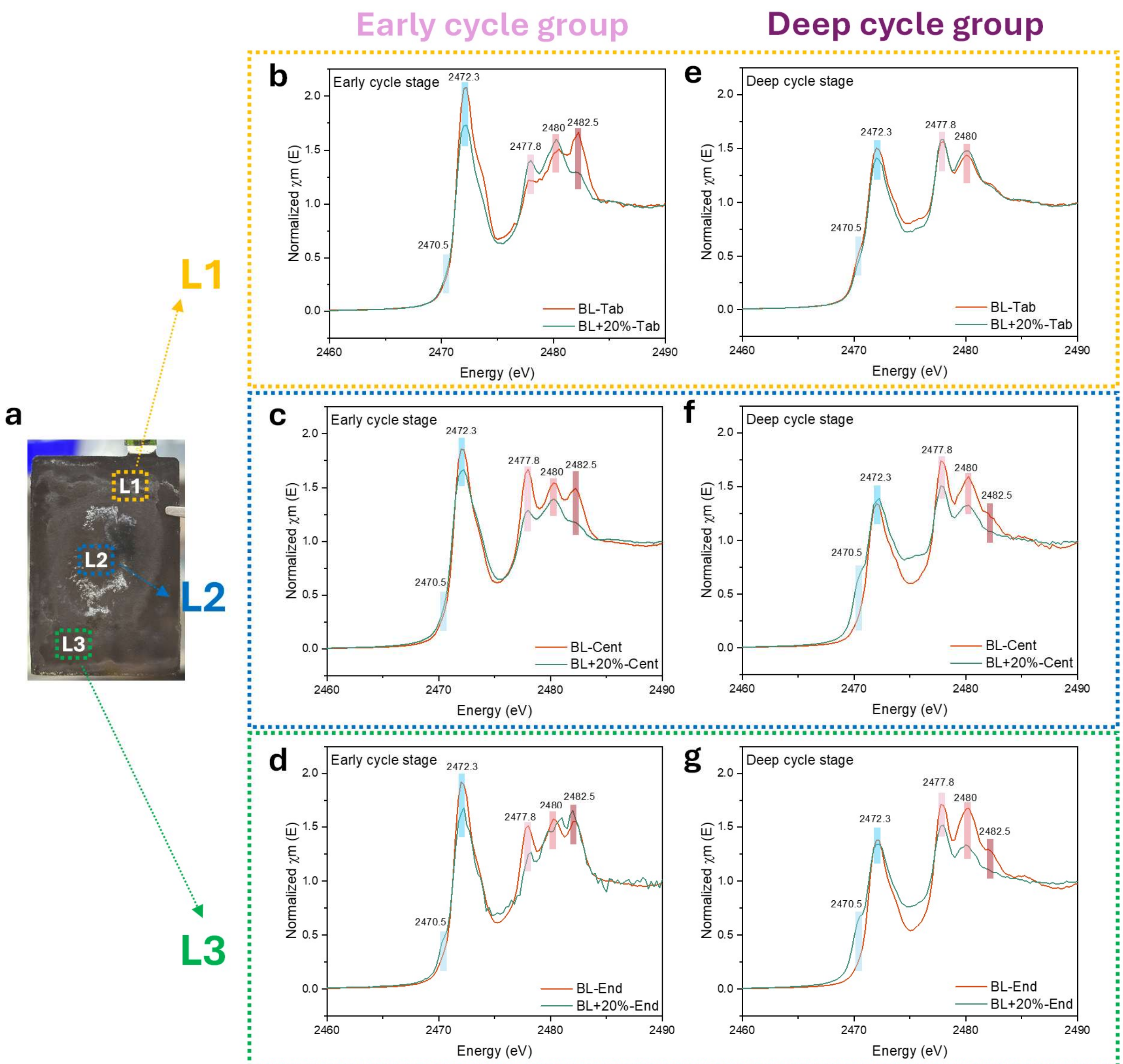

Figure S29 (a) Photograph of the sulfur cathode harvested from a pouch cell, showing the selected regions L1–L3, and (b–g) XANES spectra at the sulfur K-edge for each region during (b–d) the early cycling stage and (e–g) the deep cycling stage.

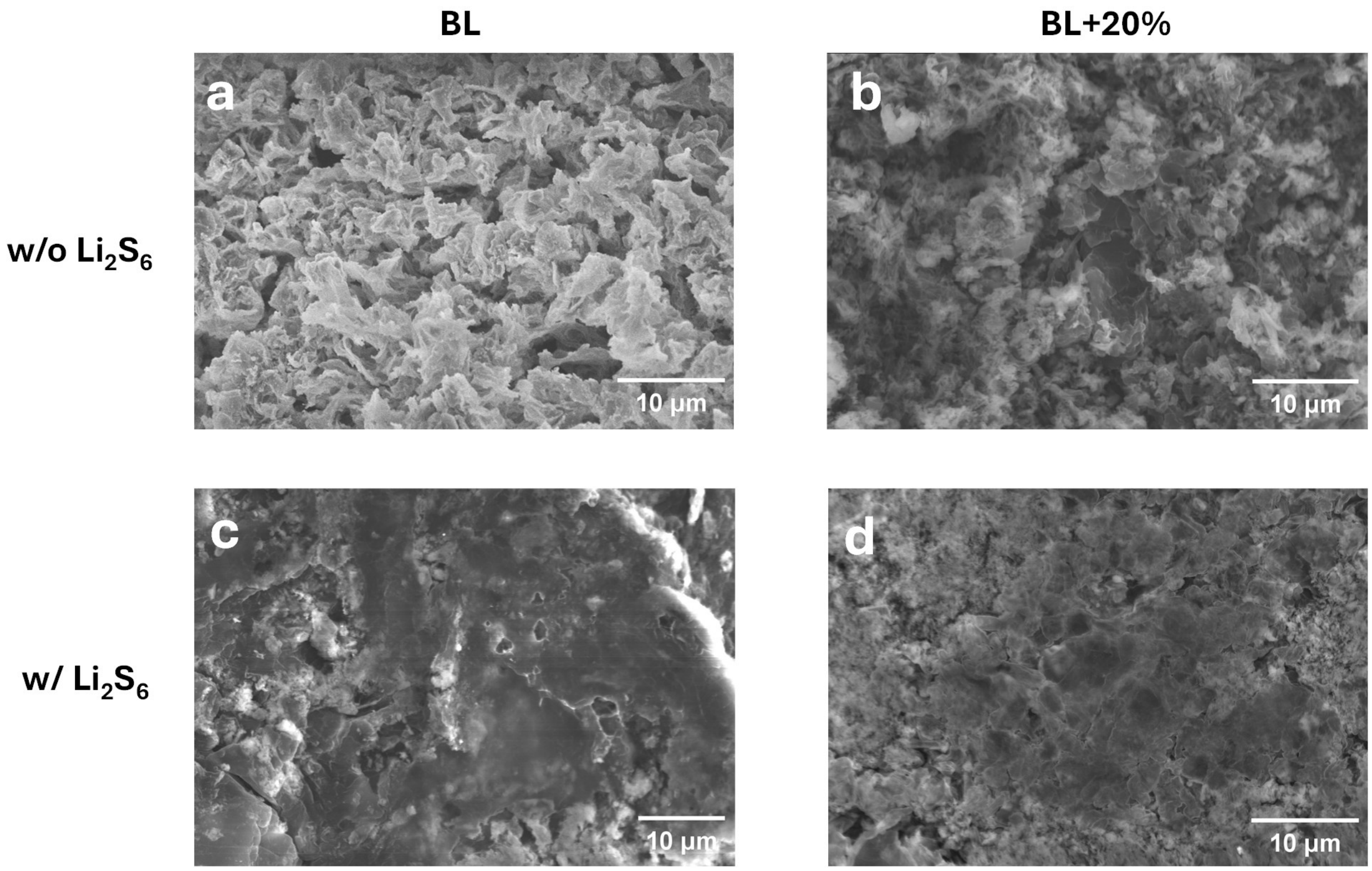

Figure S30 Lower-magnification SEM images without Li_2S_6 for (l) BL and (m) BL+20%, and with Li_2S_6 for (n) BL and (o) BL+20%.

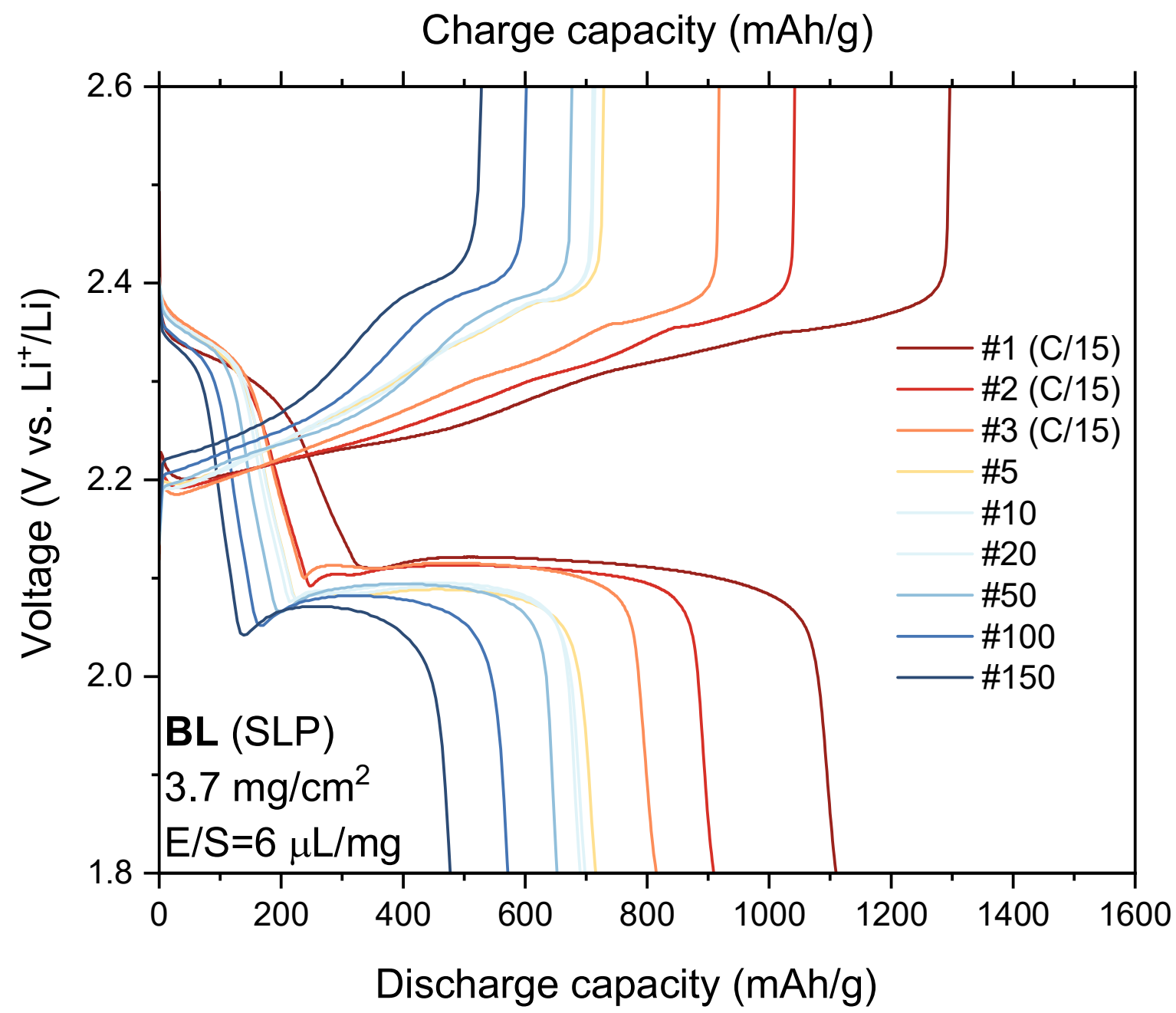

Figure S31 Specific voltage profiles of the SLP using BL.

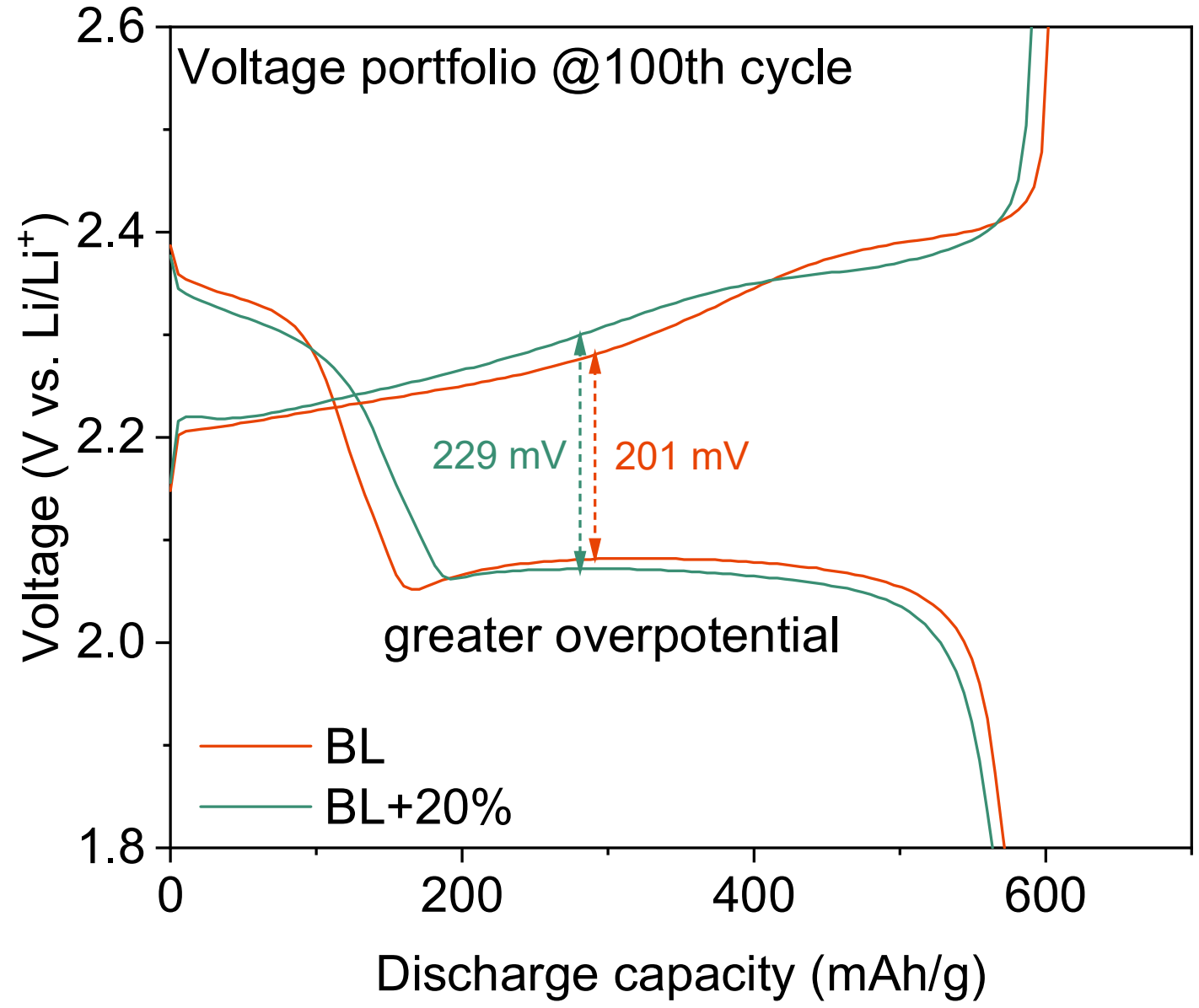

Figure S32 Discharge profile comparison at 100th cycle in Bl and BL+20%.

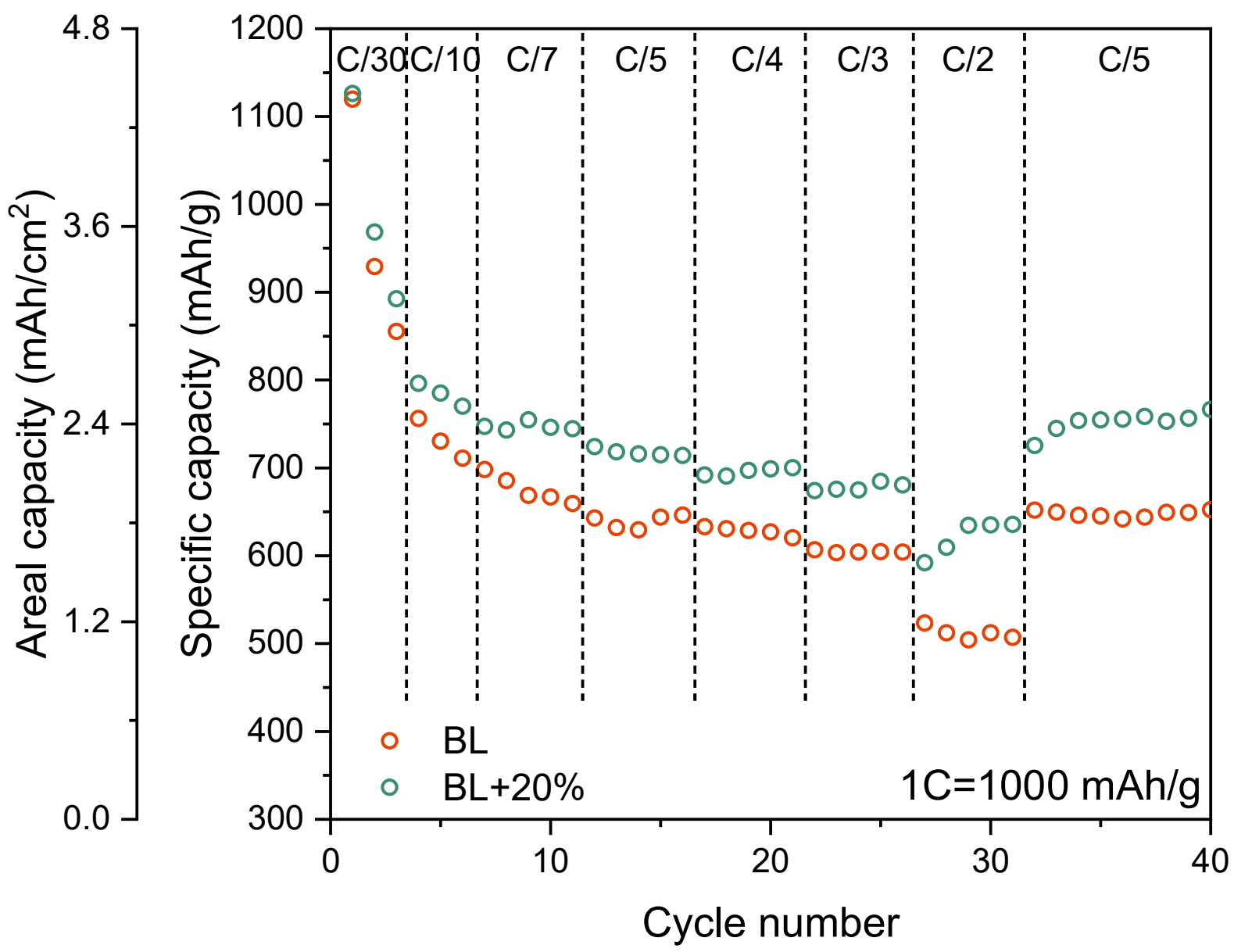

Figure S33 Rate capability performance of BL and BL+20% using the modified cycling protocol proposed by Liao and Manthiram, with 1 C defined as 1000 mAh g^{-1}.[7]

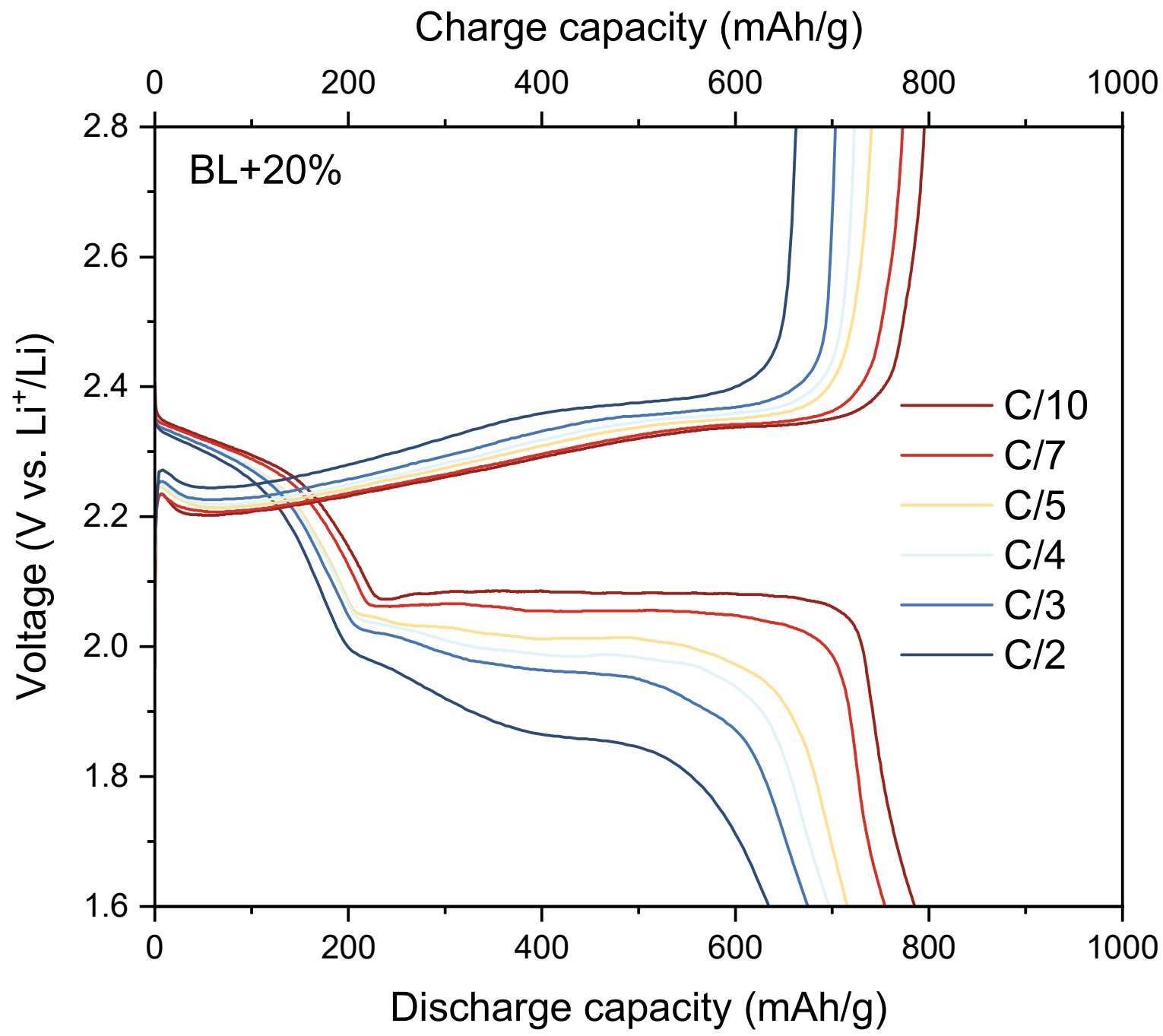

Figure S34 Specific voltage profiles of the MLP using BL+20%.

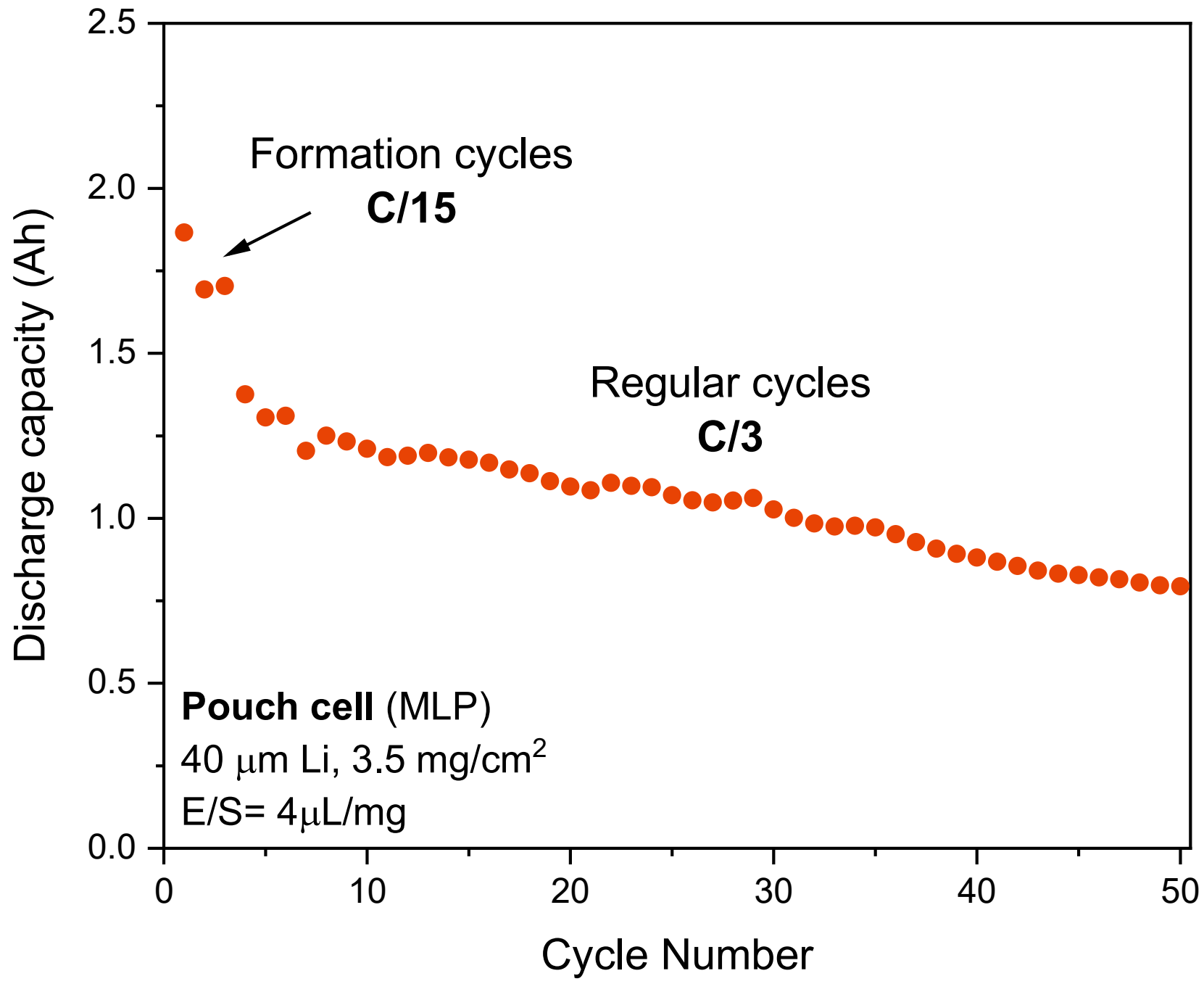

Figure S35 Cycling performance of MLP comprising eight double-layer–coated cathodes with BL.

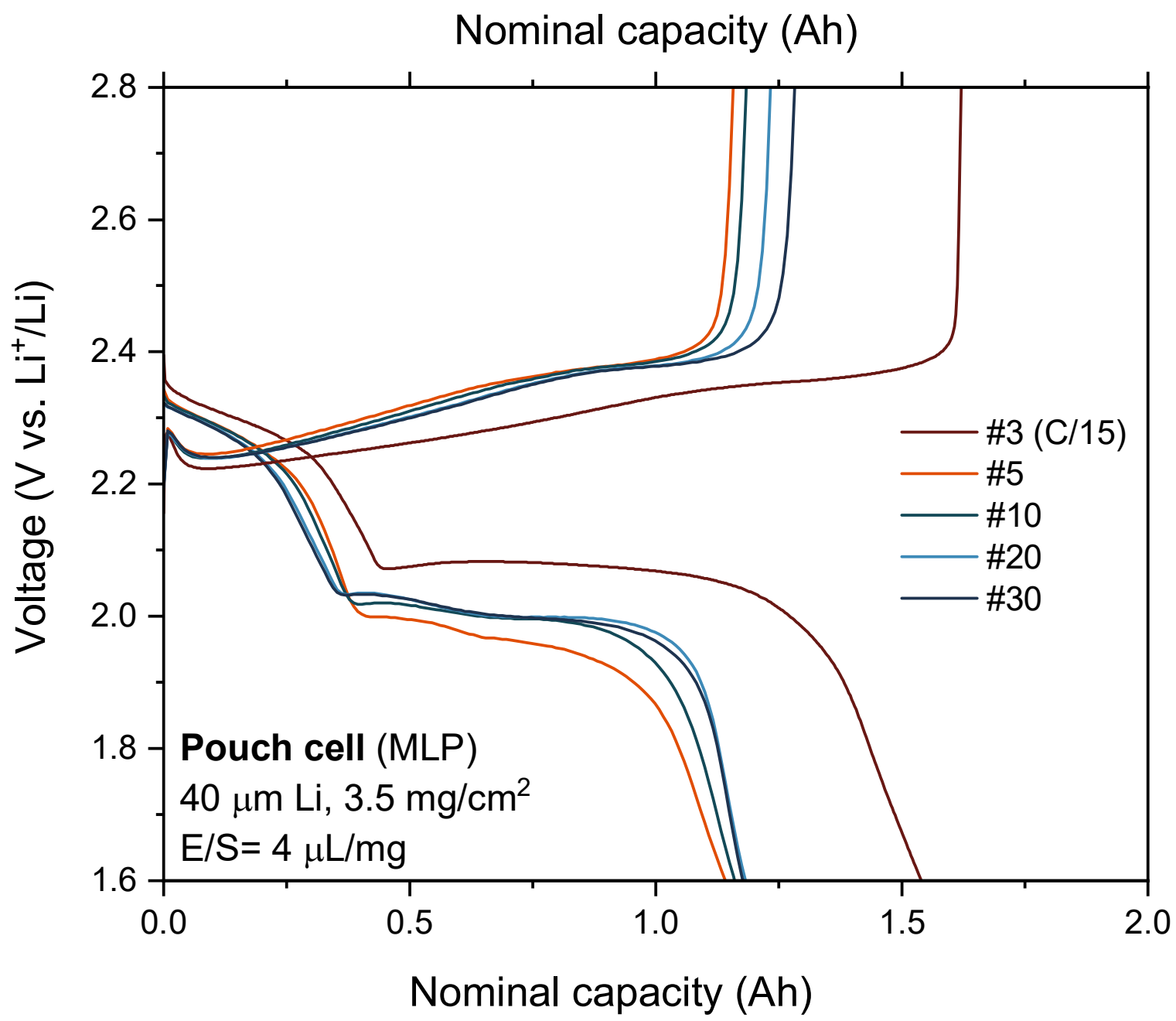

Figure S36 Representative voltage profiles of MLP at certain cycles.

3. Supplementary tables

Table S1 Summary of Arrhenius fitting results of the four electrolytes from Li_2S_6 symmetric cells ranging from 0 to 60 ℃.

	**BL**	**BL+10%**	**BL+20%**	**BL+50%**
**Intercept**	-19.86092 ± 0.86911	-19.78028 ± 1.12415	-19.58564 ± 0.85435	-19.32341 ± 0.75166
**Slope**	6.13223 ± 0.26141	6.21931 ± 0.33813	6.23181 ± 0.25697	6.29262 ± 0.22609
**R-Square**	0.98977	0.9829	0.98984	0.99194

4. Supplementary references

[1] a)H. Dai, C. Gallagher, S.-M. Bak, L. Gomes, K. Yang, R. Dong, S. Badhrinathan, Q. Zhao, Y. Du, G. P. Pandey, *Energy Storage Materials* **2025**, *76*, 104123; b)H. Dai, L. Gomes, D. Maxwell, S. Zamani, K. Yang, D. Atienza, N. Dale, S. Mukerjee, *ACS Applied Materials & Interfaces* **2024**, *16*, 8639–8654.

[2] L. Shi, S. M. Bak, Z. Shadike, C. Wang, C. Niu, P. Northrup, H. Lee, A. Y. Baranovskiy, C. S. Anderson, J. Qin, S. Feng, X. Ren, D. Liu, X. Q. Yang, F. Gao, D. Lu, J. Xiao, J. Liu, *Energy & Environmental Science* **2020**, *13*, 3620-3632. https://doi.org/10.1039/d0ee02088e.

[3] B. Ravel, M. Newville, *Journal of synchrotron radiation* **2005**, *12*, 537-541.

[4] a)M. Cuisinier, P. E. Cabelguen, S. Evers, G. He, M. Kolbeck, A. Garsuch, T. Bolin, M. Balasubramanian, L. F. Nazar, *The Journal of Physical Chemistry Letters* **2013**, *4*, 3227-3232. https://doi.org/10.1021/jz401763d; b)T. A. Pascal, K. H. Wujcik, J. Velasco-Velez, C. Wu, A. A. Teran, M. Kapilashrami, J. Cabana, J. Guo, M. Salmeron, N. Balsara, D. Prendergast, *The Journal of Physical Chemistry Letters* **2014**, *5*, 1547-1551. https://doi.org/10.1021/jz500260s; c)Y. Gorlin, M. U. M. Patel, A. Freiberg, Q. He, M. Piana, M. Tromp, H. A. Gasteiger, *Journal of The Electrochemical Society* **2016**, *163*, A930-A939. https://doi.org/10.1149/2.0631606jes; d)X. Yang, X. Gao, Q. Sun, S. P. Jand, Y. Yu, Y. Zhao, X. Li, K. Adair, L. Y. Kuo, J. Rohrer, J. Liang, X. Lin, M. N. Banis, Y. Hu, H. Zhang, X. Li, R. Li, H. Zhang, P. Kaghazchi, T. K. Sham, X. Sun, *Advanced materials* **2019**, *31*. https://doi.org/10.1002/adma.201901220; e)D. Cao, X. Sun, F. Li, S.-M. Bak, T. Ji, M. Geiwitz, K. S. Burch, Y. Du, G. Yang, H. Zhu, *Angewandte Chemie* **2023**. https://doi.org/10.1002/anie.202302363.

[5] T. H. Wan, M. Saccoccio, C. Chen, F. Ciucci, *Electrochimica Acta* **2015**, *184*, 483-499.

[6] M. Zhao, H. J. Peng, J. Y. Wei, J. Q. Huang, B. Q. Li, H. Yuan, Q. Zhang, *Small Methods* **2020**, *4*, 1900344.

[7] K. Liao, A. Manthiram, *Advanced Energy Materials* **2025**, 2502062.
